# A review of authorship in herpes simplex virus type-2 (HSV-2) research conducted in low-income and middle-income countries between 2000 and 2020

**DOI:** 10.1136/bmjgh-2023-012719

**Published:** 2024-07-04

**Authors:** Belinder Nahal, Ela Mair Owen, Muna Jama, Angela Obasi, Emily Clarke

**Affiliations:** 1 University of Liverpool, Liverpool, UK; 2 London School of Hygiene & Tropical Medicine, London, UK; 3 Liverpool School of Tropical Medicine, Liverpool, UK; 4 International Rescue Committee, Mogadishu, Somalia; 5 Department of International Public Health, Liverpool School of Tropical Medicine, Liverpool, UK; 6 AXESS Sexual Health, Liverpool University Hospitals NHS Foundation Trust, Liverpool, UK

**Keywords:** Review, Other study design, Public Health, Infections, diseases, disorders, injuries, Accountability

## Abstract

**Introduction:**

Equitable inclusion of low-income and middle-income country (LMIC) researchers and women in research authorship is a priority. A review of progress in addressing WHO-identified priorities provided an opportunity to examine the geographical and gender distribution of authorship in herpes simplex virus type-2 (HSV-2) research.

**Methods:**

Publications addressing five areas prioritised in a WHO workshop and published between 2000 and 2020 were identified. Data on author country, gender, authorship position and research funding source were collected by manuscript review and internet searches and analysed using IBM SPSS V.26.

**Results:**

Of, 297 eligible papers identified, (n=294) had multiple authors. Of these, 241 (82%) included at least one LMIC author and 143 (49%) and 122 (41%) had LMIC first and last authors, respectively. LMICs funded studies were more than twice as likely to include an LMIC first or last author as high-income country-funded studies (relative risk 2.36, 95% CI 1.93 to 2.89). Respectively, 129 (46%) and 106 (36%) studies had female first and last authors. LMIC first and last authorship varied widely by HSV-2 research area and increased over time to 65% and 59% by 2015–2020.

**Conclusion:**

Despite location of the research itself in LMIC settings, over the 20-year period, LMIC researchers held only a minority of first and last authorship positions. While LMIC representation in these positions improved over time, important inequities remain in key research areas and for women. Addressing current and historical power disparities in global health research, research infrastructure and how it is funded may be key addressing to addressing these issues.

**WHAT IS ALREADY KNOWN ON THIS TOPIC**

- Multiple studies have documented significant inequities in authorship attribution in health research conducted in low-income and middle-income countries (LMICs), particularly in first and last author positions. The equity of authorship of research on herpes simplex virus type-2 (HSV-2) has not yet been explored.

**WHAT THIS STUDY ADDS**

- This study shows that LMIC researchers have been under-represented in high-value authorship positions in HSV-2 research conducted in LMICs between 2000 and 2020, especially when this research is funded by high-income countries. However, while the representation of LMIC authors has improved over time, important gaps remain in some research areas.

**HOW THIS STUDY MIGHT AFFECT RESEARCH, PRACTICE OR POLICY**

- Inequity in research authorship may result from the persisting effects of colonialism on global health. Identifying, acknowledging and describing these inequities and their associations is a necessary first step in developing appropriate strategies to address them. We urge funders, researchers, journal editors and other stakeholders to consider such issues when commissioning, conducting and publishing research undertaken in LMICs.

## Introduction

### Value of authorship positions

Authorship of publications is a commodity of great value within the research ecosystem.^1^ ^2^ Authorship positions have different currency, with first, last and corresponding author being the most highly valued.^3^ Typically, the first author contributes the most to the work, including the writing of the manuscript^4^; the last author provides senior oversight of the research.^5^

### Authorship inequities

Author distribution for research conducted in low-income and middle-income countries (LMICs) is currently inequitable both in terms of LMIC author inclusion and representation in high-value positions.^2 6–8^ For example, Mbaye *et al* found that only 49.8% of studies included at least one African author in their analysis of infectious disease research in Africa.^9^ This contravenes The International Committee of Medical Journal Editors (IJCME) recommendation that one coinvestigator be from the study country, owing to the invaluable insight that local researchers provide.^10^ Ravi *et al* found that over two-thirds of first and last authors were affiliated with at least one high-income country (HIC) institution in their analysis of authorship in global surgery.^11^

Women are also adversely affected by authorship inequities and are particularly under-represented in high-impact journals.^12^ For example, a recent analysis of articles published in the Lancet Global Health found that women represented only 25.4% and 29.7% of first authors in publications from low-income countries (LICs) and middle-income countries (MICs), respectively.^13^

While calls have been made to address authorship inequities,^2^ it is important to continue to document their nature and extent if we are to optimise, target and monitor interventions to address them.

### Herpes simplex virus type-2 research in LMICs

A recent analysis of progress in herpes simplex virus type-2 (HSV-2) research conducted in LMICs over a 20-year period provided an opportunity to evaluate the distribution of authorship in publications addressing research priorities in this area.

Because of the public health burden of HSV-2, and its association with HIV, an international technical workshop was held by the WHO in February 2001 to review existing knowledge regarding the epidemiology and control of HSV-2 in LMICs and its interactions with HIV.^14^ The objectives of the workshop were to review existing knowledge, identify important knowledge gaps and establish priorities for future research and control programmes. Research priorities were identified in five areas: epidemiology and natural history of HSV-2, interactions between HSV-2 and HIV, HSV-2 control measures for developing countries, mathematical modelling and HSV-2 diagnosis.^15^

### Aim

The current study aimed to describe the geographical and gender distribution of authorship among LMIC-based HSV-2 research in the key priority areas identified in the WHO 2001 workshop.^14^ It intended to document the distribution of key authorship positions by geography (HIC vs LMIC) research area, time period and funding source (HIV vs LMIC) between the years 2000 and 2020. The outcome measures included first/last authorship position, (HIC vs LMIC) author affiliation, area of study and author gender.

## Methods

The current study was nested within two complementary studies that assessed progress in HSV-2 research conducted in LMICs in the areas prioritised by WHO workshop.^15 16^

### Search strategy

The detailed methods are presented in the complementary reviews.^15 16^ However, in brief, a database search of CINAHL Complete, MEDLINE Complete, Global Health and The Cochrane Library was undertaken using specific search terms. Articles were eligible for inclusion if they were published in English and addressed one of the five WHO-identified research priorities for HSV-2 in LMICs between 2000 and 2020. LMICs were defined using the United Nations Country Classification system.^17^ Studies written in languages other than English, published outside of the specified time frame or studies describing aspects of HSV-2 other than the WHO-specified research priority areas were excluded. The articles identified by the database searches were manually assessed by two independent researchers, and conflicts were resolved through a discussion between all researchers. All articles finally included in the complementary reviews were included in the current study.

### Data extraction and methods for identifying authorship

All papers included in the literature reviews^15 16^ were examined and data manually extracted by BN, EMO and MJ including the names of the first author, last author and other authors. Joint first or last authors were identified by manually scanning the author contribution and author affiliation sections. If one or more of the joint authors were from an LMIC, the article was coded as including an LMIC first/last author for the main analysis, and breakdown of the individual author institutions was reported separately. The same approach was applied to the presence of a female first/last author. Corresponding authors were not considered as a separate category because the importance of this authorship position has developed in very recent years and has not been of consistent significance throughout 2000–2020.^18 19^

Data were also extracted on author’s country affiliation, the geographical location of study participants, year of publication and country of funding source. Country affiliation of authors was assigned based on their current institution listed in the paper, and where LMIC and HIC institutions were listed for the same author, they were assigned to the LMIC institution. Countries were further categorised into low, lower middle and higher middle income according to the World Bank classification.^20^ Gender of authors was determined using the author’s first name and using freely available identifiers such as names and photographs. Where the gender of a named author was ambiguous, this was discussed among data extractors and with AO and EC. If still unresolved, a general internet search for the author’s profile was conducted. If the profile was not found the gender was classified as unknown. Data were imported into a Microsoft Excel spreadsheet and analysed using IBM SPSS V.26, using simple summary statistical analysis. Data were further stratified by time period, geographical area, study type and funding country. Pearson’s χ^2^ test for association was used to compare the number of LMIC first authors in different time periods and country income groups.

### Patient and public involvement

Patient and public involvement was not appropriate for this study as no new patient data were collected.

## Results

### Search Results

**Figure 1:**
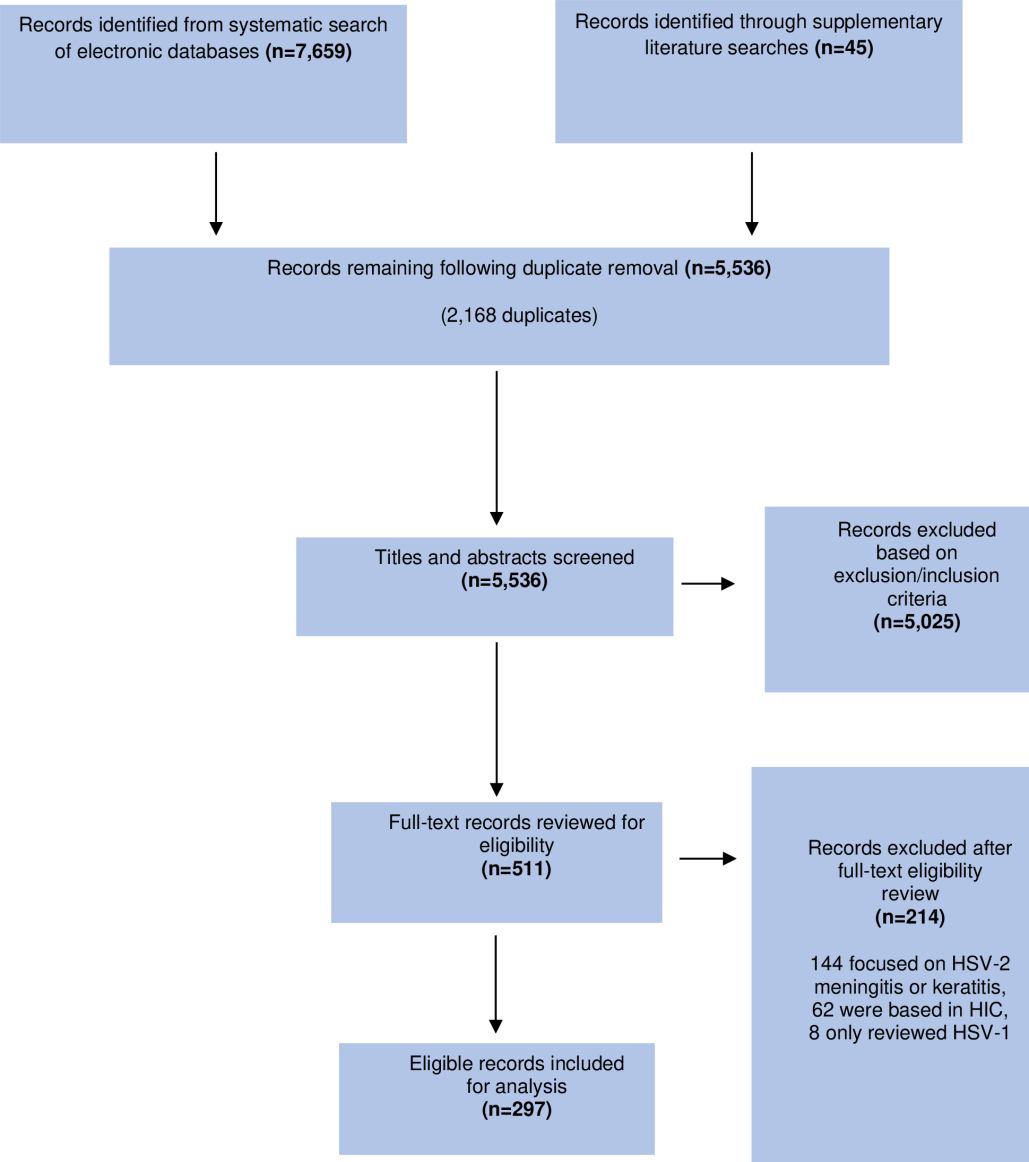
Flow chart showing study selection process. The total number of records included for analysis is less than the total of included studies for Paper A and B because of duplicate papers between each research area.

The search strategy yielded a total of 7704 records, with 2168 being duplicates. The titles and abstracts of the remaining 5536 records were screened for eligibility using the criteria described above. A total of 5025 studies were subsequently excluded to leave 511 studies. The full-text versions of these records were further reviewed for inclusion resulting in the final number of 297 included records. The included studies were conducted across 49 different LMICs in 5 continents.^15 16^ The process is summarised in figure 1. Details of included studies are presented in online supplemental file 1.

10.1136/bmjgh-2023-012719.supp1 Supplementary data

### LMIC authorship

Of the 297 eligible studies, 294 (99%) included more than 1 author and 3 (1%) had a sole author. Of the studies that included more than 1 author, 241 (82%) included at least 1 author from an LMIC in any position. 165 studies (56%) included a researcher from an LMIC in either 1 or both the first and last author position(s); 129 studies (44%) did not have first or last authors from LMICs. The first author position was held solely or jointly by an LMIC researcher in 143 (49%) studies; 122 (41%) had sole/joint LMIC last authors. An LMIC researcher occupied both first and last author position in 103 (35%) studies (see table 1). Only nine papers (3%) included joint first authors, two (<1%) joint last authors and two (<1%) joint first and last authors. Of the nine occurrences of joint first authorship, four (44%) included HIC authors only, three (33%) included a combination of HIC and LMIC authors, and two (22%) included LMIC authors only. Of the four occurrences of joint last authorship, two included HIC authors only and two included a combination of HIC and LMIC authors.

**Table 1:** Summary and comparison of authorship characteristics for the five HSV-2 research priority areas

	Epidemiology	Diagnostics	Control measures	Interaction with HIV	Mathematical modelling	All studies
Number of included articles (duplicates removed)	94	63	44	79	17	297
Number of different LMICs from which studies were carried out	37	23	13	21	8	49
Percentage of studies with ≥1 author from LMIC (%)	89	76	66	94	35	82
Percentage of studies where first or last author is from LMIC (%)	75	52	41	53	18	56
Percentage of studies where first author is from LMIC (%)	67	48	32	41	18	49
Percentage of studies where last author is from LMIC (%)	59	42	27	36	19	41
Percentage of studies where first and last authors are from LMIC (%)	52	37	18	23	19	35
Percentage of studies where funding source is acknowledged (%)	86	86	80	86	89	85
Percentage of studies with LMIC funding source (of those that were funded) (%)	45	33	6	16	13	27
Crude relative risk of LMIC first or last author when funding is from LMIC compared with HIC	–	–	–	–	–	2.36 (95% CI 1.93 to 2.89)
Gender of first authors (%)	Male: 43 Female: 47	Male: 43 Female: 48	Male: 43 Female: 55	Male: 52 Female: 44	Male: 47 Female: 53	Male: 48 Female: 46
Gender last authors (%)	Male: 61 Female: 29	Male: 54 Female: 35	Male: 55 Female: 43	Male: 56 Female: 39	Male: 65 Female: 29	Male: 58 Female: 36
Gender of first authors among studies where first author is from LMIC (%)	Male: 46 Female: 43	Male: 43 Female: 43	Male: 43 Female: 50	Male: 59 Female: 31	Male: 67 Female: 33	Male: 50 Female: 41
Gender of first authors among studies where first author is from HIC (%)	Male: 37 Female: 57	Male: 42 Female: 52	Male: 43 Female: 57	Male: 47 Female: 53	Male: 43 Female: 57	Male: 43 Female: 55
Gender of last authors among studies where first author is from LMIC (%)	Male: 65 Female: 19	Male: 38 Female: 42	Male: 58 Female: 33	Male: 54 Female: 39	Male: 67 Female: 33	Male: 59 Female: 35
Gender of last authors among studies where first author is from HIC (%)	Male: 56 Female: 44	Male: 67 Female: 31	Male: 53 Female: 47	Male: 58 Female: 40	Male: 69 Female: 31	Male:.58 Female: 41

The crude relative risk of LMIC first or last author when funding is from an LMIC compared with an HIC does not include studies with joint first/last authors.

HIC, high-income country; HSV-2, herpes simplex virus type-2; LMIC, low-income and middle-income country.

The three sole author papers were primary research papers. Two were by male LIC authors, and one by a female MIC author.^21–23^

### LMIC authorship by HSV-2 research area

LMIC authorship inclusion varied widely by research area. Epidemiological studies displayed the greatest degree of LMIC author participation. Of the 94 epidemiological studies included, 84 (89%) included at least 1 LMIC author and 70 (75%) included an LMIC author in at least 1 of the first or last author positions. Specifically, 62 (67%) had an LMIC first author, 55 (59%) had an LMIC last author and 48 (52%) had LMIC first and last authors. Publications were least likely to include an LMIC author in the area of HSV-2 mathematical modelling (35%). Author distributions across the five prioritised areas are shown in table 1.

### Gender of authorship

**Figure 2:**
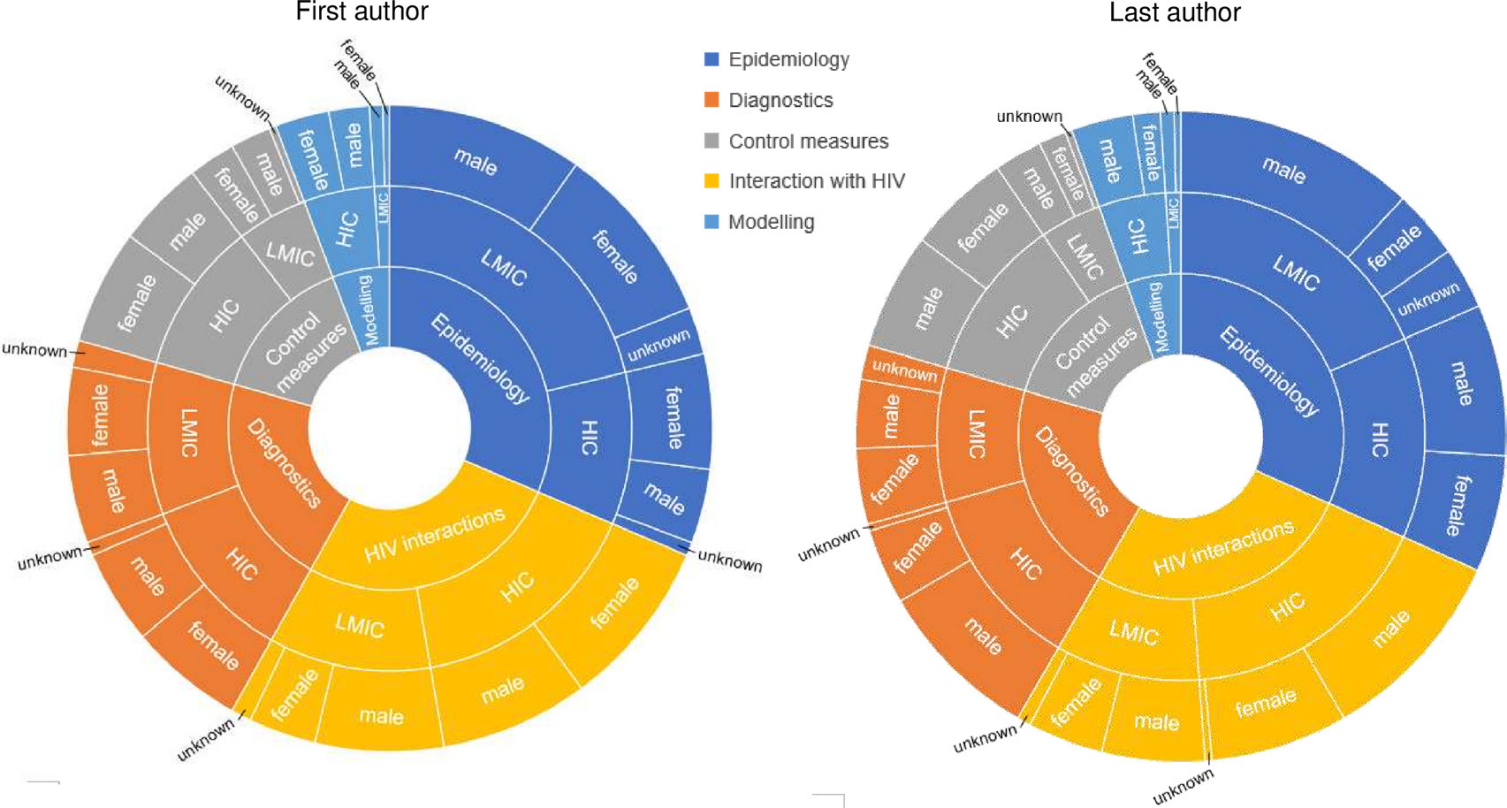
Sunburst diagrams displaying the relative proportions of geographical affiliation and gender of first and last authors. The innermost layer illustrates the proportions of included studies from each research area; the middle layer illustrates the proportions of first (left hand side) and last (right hand side) authors from LMIC and HIC for each research area; and the outermost layer illustrates the gender proportions for each respective group of authors.

Of first authors, 135 (46%) were female, 141 (48%) were male and the gender of 18 (6%) first authors was unidentifiable. Among last authors, 106 (36%) were female, 170 (58%) were male and the gender of 18 (6%) was unidentifiable. In studies where the first author was from an LMIC, 58 (41%) were female, 71 (50%) male and 14 (10%) unidentifiable; whereas where the first author was from an HIC, 83 (55%) were female, 65 (43%) male and 3 (2%) unidentifiable. In studies where the last author was from an LMIC, the proportion of female and male last authorship was 43 (35%) and 72 (59%), respectively, with 7 (6%) unidentifiable; whereas in studies where the last author was from an HIC, the rate of female and male last authorship was 70 (41%) and 99 (58%) respectively, with 2 (1%) unidentifiable. Figure 2 summarises the gender distribution by first and last authorship and by the research area.

### Funding

The funding source was acknowledged in 85% (n=250) of the studies. Of these, 73% (n=183) were funded by HIC organisations, and 27% (n=67) by LMIC sources. LMIC sources of funding were most prevalent in epidemiological studies (45%, n=42), and least prevalent in clinical studies addressing HSV-2 control measures (6%, n=3) and interaction with HIV (16%, n=13). Studies that were funded by an LMIC source were more than twice as likely to include a first or last author from LMIC compared with those funded by an HIC (relative risk=2.36, 95% CI 1.93 to 2.89) (3.7% (n=11) of studies included joint first or last authors—these were not included in the relative risk calculation).

### Specific income groups

**Figure 3:**
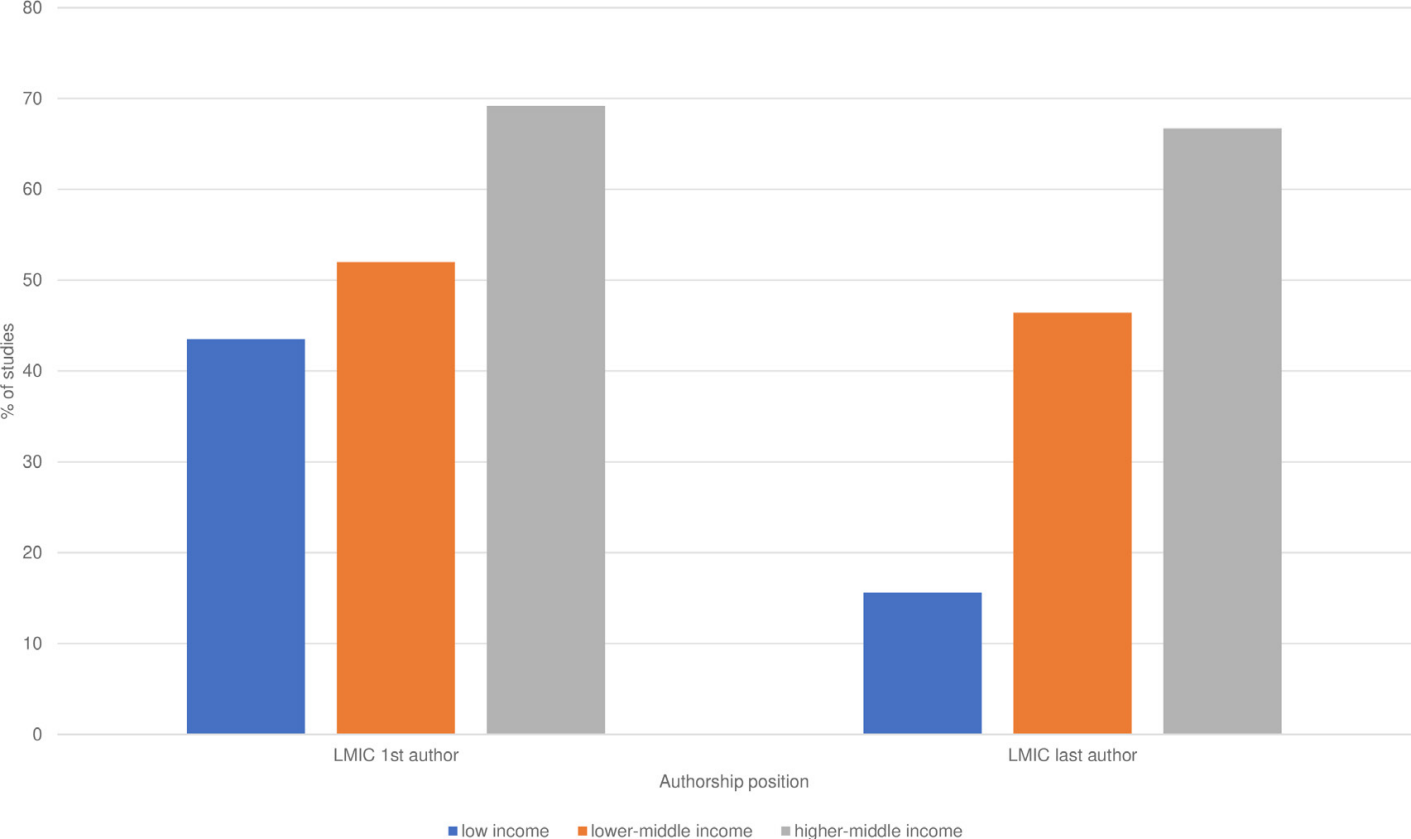
Bar chart showing proportion of studies with first and last authors from LMIC in three study country income groups

Among LMICs, study countries with higher income were more likely to produce studies with first (Pearson’s χ^2^=9.97, p<0.05) and last (Pearson’s χ^2^=31.62, p<0.05) authors from LMIC. The relationship was most noticeable when studying last authors (see figure 3).

### Authorship over time

**Figure 4:**
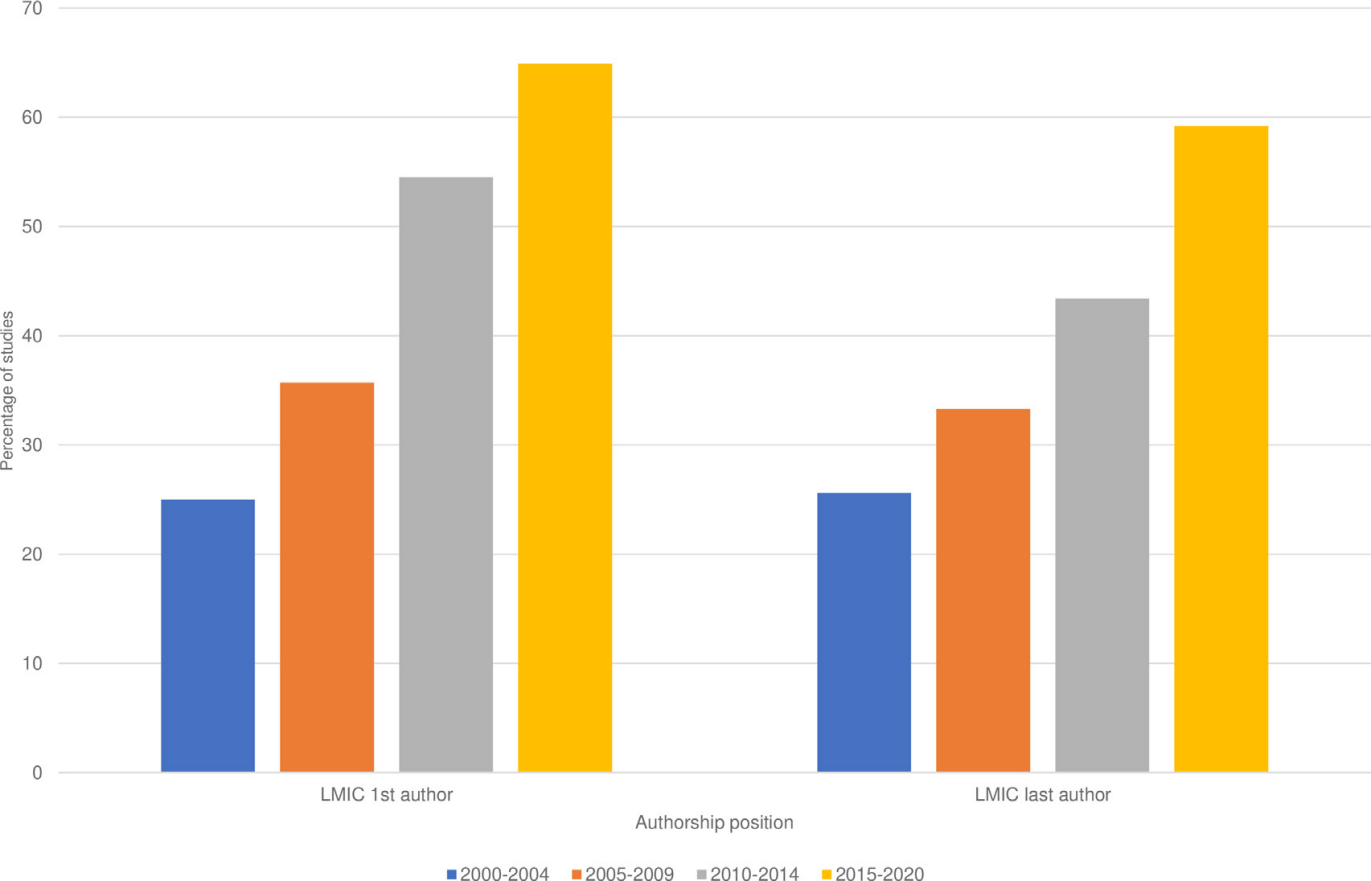
Bar chart showing percentage of studies with first and last authors from LMIC in four publication time periods

The proportion of studies with LMIC first (Pearson’s χ^2^=23.69, p<0.05) and last (Pearson’s χ^2^=15.54, p<0.05) authors increased with each 5-year time period from the year 2000 to 2020 (see figure 4). In 2000–2004, 25% of publications had LMIC first authors which increased to 65% by 2015–2020. For last authorship proportions increased from 26% to 59% across the time period.

## Discussion

This study examined the geographical and gender distribution of authors for HSV-2 research in LMICs published between 2000 and 2020 in the areas of epidemiology, control measures, mathematical modelling, diagnosis and interactions between HSV-2 and HIV prioritised by a WHO international workshop in 2001.^14^ To our knowledge, it is the first study to have examined authorship in HSV-2 which continues to be of global health significance. The 20-year time frame and specific focus on prioritised research areas allowed us to examine differences in authorship by subject area, funding source and over time. Few such analyses have been conducted for any specific research area. For example, Ravi *et al* have conducted an analysis of global surgery publications over a 4-year period^11^ and Tuyishime *et al* have conducted an analysis of global oncology publications in Africa over a 5-year period.^24^

### Authorship overall and author positions

Overall, almost one-fifth (18%) of the 297 research publications relating to HSV-2 in LMICs in these prioritised areas did not include any author from an LMIC. This absence of local authors has been noted in other global health research areas. For example, Naidoo *et al* found that one-fifth of papers had no African author in their analysis of COVID-19 literature in Africa.^25^ Research papers written without local researchers may lack adequate interpretation of context or other local factors which are of value in study design and in the interpretation of results.^8 26 27^ The absence of local researchers also contravenes IJCME guidance.^10^ Lack of local researcher insight may be particularly compromising when studying a widely stigmatised sexually transmitted infection such as HSV-2, where participants may be more comfortable interacting with individuals who share their culture and/or fully understand their context. Specific understanding of HSV-2 stigma and related issues in a local or community context could facilitate recruitment, ongoing engagement in the study and allow appropriate support to be provided for study participants.

First and last author positions are generally the most highly valued. Of the 294 publications that included more than one author, first author position was held by an LMIC researcher in only half (49%) and last author in even fewer (41%) of studies. Just over one-third (35%) of publications had both first and last LMIC authors. The inequities in Global Health research are being increasingly recognised and our findings are comparable to studies relating to some research areas.^11 25^ For example, Tuyishime *et al* found 44.8% and 40.7% of first and last authors were from Africa in their analysis of oncology research conducted in Africa.^24^ However, our data suggest that HSV-2 research compares favourably with publications in some other areas, for example, global surgery where (21%) of studies had LMIC authors in first and last positions^11^ and COVID-19 research as discussed above.^25^

Finally, joint first or last authorships were very few (<4%). The practice of joint position authorships to signal equal contributions and consequently share credit is becoming increasingly common. However, implementation has faced challenges from structures that assume research is led by individuals, and hierarchical cultures that have been slow to recognise that different disciplines or individuals can contribute equally, even in leadership.^28^

For HIC academic institutions, equity in authorship should be promoted through induction and in-service training. Assessment of authorship track records at key ‘pinch points’ such as promotion or appraisals should valorise evidence of fair inclusion of LMIC partners as authors, not just HIC researcher first or senior authorships. HIC institutional rewards for evidence of collaborative and fair practice rather than author position could play an important role in changing cultures.^29^ Increased use of joint authorship positions may be a valuable tool in aiding this development.

### Distribution by study type

LMIC first/last authorship also varied between HSV-2 research areas. LMIC authors were most frequently represented in these key authorship positions among epidemiological studies, with 75% of studies having either a first or last author from an LMIC, and 67% and 59% having first and last authors from an LMIC, respectively. The areas demonstrating the poorest representation of LMIC authors in first/last author position were mathematical modelling studies with only 18% of studies having either a first or last author from an LMIC. Mbaye *et al* also found better LMIC author representation among epidemiological studies compared with clinical studies in their analysis of infectious disease research conducted in Africa.^9^ The reasons for this are unclear; however, it may relate to the availability of research infrastructure or skills in high technology areas such as clinical sciences or modelling.^30^ Social, political and economic inequities that face LMICs restrict research capacity building, which is an important factor that helps empower LMIC authors to lead research studies.^31^

### Funding

LMIC first/last authorship varied depending on the source of funding. When HSV-2 research was funded by an LMIC organisation, it was 2.36 times more likely to credit the first or last authorship position to an LMIC researcher. This is in line with the findings of Hedt-Gauthier *et al* who found that clinical trials in Africa funded by US institutions were less likely to have LMIC authors in first or last authorship positions.^32^ This highlights the need for HIC funders in particular to adopt criteria that specify local author involvement.

### Gender

Overall, first authorship attribution for men (48%) and women (46%) was similar. This differs markedly from the pattern that has been observed across many areas of health research^33^ including global health.^34 35^ However, only one-third (36%) of last authors were women. This lack of senior authorship among women has been attributed to prevailing research cultures and practices that disadvantage women across the research continuum from recruitment to research institutions,^36 37^ securing grant funding^38^ and negotiations relating to author position order to bias against female authors in the paper review.^39^

Gender inequalities varied by geography. The proportion of female researchers in first author positions was higher among publications where the first author is from an HIC (55%) compared with an LMIC (41%). Similarly, HIC last authors were more likely to be female (41%) than LMIC last authors (35%). Ravi *et al* found a lower proportion of LMIC female authors at every career grade^11^ which suggests additional or more severe factors adversely affecting women in LMIC research compared with those in HICs.

### Changes over time

Overall, our analysis of how first/last authorship of HSV-2 research has changed over time found that, the majority of papers had an LMIC first or last authorship by the most recent 5-year period. This compares favourably with recent analyses of global health research in many areas.^9 24 25^ The increase in the proportion of LMIC first or last authorship over time may be a result of strategies used over the last decade to build research capacity and promote academic justice in LMICs in attempts to meet global health targets.^40^ HSV-2 is also unusual in global health research in that women appear to have achieved overall parity in first authorships across the time period. The gender parity observed may be related to the fact that the prevalence of HSV-2 is higher among women, therefore, female researchers may be more likely to be passionate about researching the subject.

### Limitations

While several studies have reviewed LMIC versus HIC authorship, this is the first study to examine gender, funding source and disease subspecialty for HSV-2 over time and on multiple continents. Despite these strengths, the study has several limitations. First, authors were assigned an LMIC origin when they had associations with both HIC and LMIC institutions. This may have resulted in an overrepresentation of first and last LMIC authorship if author affiliation or residence was primarily to the HIC institution. Additionally, this study only used the male–female gender binary and so was unable to recognise non-binary or transgender individuals. The subjectivity of the researcher-designated gender classification of authors is also a potential source of error. Unfortunately, pronouns were rarely shared in author profiles, and we did not have the resources to contact each corresponding author to allow self-identification. Consequently, 5%/6% of first and last authors were classified as unknown which may have resulted in an underestimate of gender differences in authorship; however, the numbers involved are small. Further, corresponding authors were not considered as a separate category because the importance of this authorship position has developed largely in very recent years as was recognised in the last UK Research Excellence Framework^41^ but has not been of consistent significance throughout 2000–2020.^18 19^ We understand that the inclusion of only one or two LMIC authors within a list of many authors can be a way for HIC senior authors to avoid criticism.^42^ These small numbers of LMIC authors may mask ongoing inequities. Our data on the proportion of studies that include at least one LMIC author may therefore mask true levels of LMIC author exclusion. Also, for logistical reasons, there was no secondary verification of the data extracted from the selected studies by individual researchers. Finally, because of the limitations of the source review studies, only research in English language could be included. Significant bodies of research in languages such as French and Spanish from Francophone Africa and Latin America, respectively, may have been excluded from this study. However, the vast majority of global health research continues to be published in English. For example, Mbaye *et al* found that first and last authors from African LMICs are predominately from Anglophone countries.^9^

### Reflections on positionality

The focus of this research made it particularly important for us to reflect on our own positionality. The majority of the authorship team of the current paper is based in an HIC although two of the authors bring local perspectives through country of birth, nationality (MJ) and prolonged local residence (MJ and AO). One joint senior author (AO) participated in the 2001 WHO HSV-2 workshop as an early career fellow. The reflexivity statement examining our positionality is included as online supplemental file 2.

10.1136/bmjgh-2023-012719.supp2 Supplementary data

### Research authorship and the global health research ecosystem

Our findings are in line with the now substantial decolonisation literature which recognises that under-representation of local authors in high-value authorship positions is the product of a legacy of colonial structures that continue to influence the allocation of funding, who leads research and who is involved in the publication of research findings.^13^

Further, the value that key authorship positions have in terms of impact on academic progression and ability to secure research funding means that author under-representation further exacerbates existing inequities between LMICs and HICs in global health research.^2 32^

However, the inclusion of local authors is not merely a matter of social justice but is critical to research quality and ensuring that research is of value to the communities in which it is conducted.

## Conclusions

Our study found that authors from LMICs were under-represented as first and last authors in HSV-2 research between 2000 and 2020. This varied by research area with disparities being greatest in modelling and control studies. Also, while LMIC author representation in first or last author positions is higher in LMIC-funded research, research remains predominantly funded by HIC organisations. Over time, the proportion of LMIC first and last authorship increased with LMIC first authors in a narrow majority by the final 5-year period. Unusually, women researchers overall approach parity in first authorship, although women from HIC were more likely to be first and senior authors than those from LMIC.

Our research, therefore, supports the finding of others highlighting factors such as gender inequity and funding sources, that are interlinked with HIC/LMIC authorship inequities in key authorship positions. However, the favourable changes over time in LMIC representation in HSV-2 research authorship highlight the fact that change is possible and may point to initiatives and practices that may warrant further exploration. Our data also highlight important deficiencies in key research areas such as HSV-2 control and modelling that still need to be addressed. Elimination of current inequities will require reflection and action from all actors within the ecosystem such as has been recommended by a recent consensus statement on equity in research publication.^2^ Our results provide data that can inform such reflection and possible interventions for health research capacity building.

## Data Availability

All data relevant to the study are included in the article or uploaded as online supplemental information.
