# Supplementary material for: A review of authorship in herpes simplex virus type-2 (HSV-2) research conducted in low-income and middle-income countries between 2000 and 2020
Source: BMJ Glob Health. 2024 Jul 4;9(7):e012719. doi: 10.1136/bmjgh-2023-012719 (PMC11227752; doi:10.1136/bmjgh-2023-012719)
Supplement: Supplementary data [file bmjgh-2023-012719supp001.pdf]

Appendix - articles included and characteristics

HSV 2 Epidemiology

Authors	Year	Title	Geographical area of study population (according to UN classification)	Main findings/Prevalence	Journal
Abraham, C. D , Conde Glez, C. J , Cruz Valdez, A , Sánchez Zamorano, L , Hernández Márquez, C ,Lazcano Ponce, E.	2003	Sexual and demographic risk factors for herpes simplex virus type 2 according to schooling level among Mexican youths	Central America	HSV 2 seropositivity was 5.7% , and the ratio of females to males was 2:1	Sexually Transmitted Diseases
Agabi, Y. A , Banwat, E. B , Mawak, J. D , Lar, P. M , Dashe, N , Dashen, M. M , Adoga, M. P , Agabi, F. Y , Zakari, H.	2010	Seroprevalence of herpes simplex virus type 2 among patients attending the Sexually Transmitted Infections Clinic in Jos, Nigeria	West Africa	HSV 2 seropositivity was 141 (87.0%)	Journal of Infection in Developing Countries
Ahmed, H. J , Mbwana, J , Gunnarsson, E , Ahlman, K , Guerino, C , Svensson, L. A , Mhalu, F , Lagergard, T.	2003	Etiology of genital ulcer disease and association with human immunodeficiency virus infection in two Tanzanian cities	East Africa	In Dar es Salaam, HSV 2 prevalnce 63%, in Mbeya, HSV 2 prevalnce 34%	Sexually Transmitted Diseases
Aho, J , Koushik, A , Coutlée, F , Diakité, S. L , Rashed, S.	2014	Prevalence of HIV, human papillomavirus type 16 and herpes simplex virus type 2 among female sex workers in Guinea and associated factors	West Africa	Prevalences of HSV 2 was 84.1%	International Journal of STD & AIDS

Nahal B, *et al. BMJ Glob Health* 2024; 9:e012719. doi: 10.1136/bmjgh-2023-012719

Akinyi, B, Odhiambo, C , Otieno, F , Inzaule, S , Oswago, S , Kerubo, E , Ndivo, R , Zeh, C.	2017	Prevalence, incidence and correlates of HSV 2 infection in an HIV incidence adolescent and adult cohort study in western Kenya	East Africa	HSV 2 prevalence was 26.6% [95% confidence interval (CI): 23.9 29.4]. Overall HSV 2 incidence was 4.0 per 100 person years (/100PY) 95% CI: 2.7 6.1(MEN 58 (11.8), Female214 (40.2))	Public Library of Sciences (PLoS)
Anjulo, A. A , Tamrat, Abebe , Feleke, Hailemichael , Adane, Mihret	2016	Seroprevalence and risk factors of herpes simplex virus 2 among pregnant women attending antenatal care at health facilities in Wolaita zone, Ethiopia	East Africa	Seroprevalence of HSV 2 infection was 32.1 %	Virology Journal
Anvikara, A. R , Rao, V. G , Savargaonkara, D. D , Yadav, Rajiva , Bhondeleya, M. K , Balkrishna, Tiwaria , Atul, Karkarea , Lukea, C , Vijay, Gadgea , Mahendra, Ukeya , Purushottam, PatelaRao, G. R. R , Ramani, T. V. , Jyothi, Padmaja	2009	Seroprevalence of sexually transmitted viruses in the tribal population of Central India	South Asia	The prevalence of HSV 2 was 20.8% in STI patients compared to 12.4% in the general population.	International Journal of Infectious Diseases
Béhanzin, L , Diabaté, S , Minani, I , Lowndes, C. M , Boily, M. C , Labbé, A. C , Anagonou, S , Zannou, D. M , Buvé, A , Alary, M.Suresh, Malhotra , Tejinder, Kaur , Ahmed, K , Deese, J , Damme, L. van Crucitti, T.	2012	Decline in HIV prevalence among young men in the general population of Cotonou, Benin, 1998 2008	West Africa	In 1998, prevalence of HSV 2 was 21.1 % and In 2008, 26.4 %	PLoS ONE

Benzaken, A , Sabidó, M , Galban, E , Dutra, D. L. R , Leturiondo, A. L , Mayaud, P.	2012	HIV and sexually transmitted infections at the borderlands: situational analysis of sexual health in the Brazilian Amazon	South America	prevalence of HSV 2 was 72.1% femle, male 51.1%	Sexually Transmitted Infections
Brown, J. M , Wald, A , Hubbard, A , Rungruengthanakit, K , Chipato, T , Rugpao, S , Mmiro, F , Celentano, D. D , Salata, R. S , Morrison, C. S , Richardson, B. A , Padian, N. S.Rahman, M , Nahar, S , Ranst, M. van , Verhaegen, J.	2007	Incident and prevalent herpes simplex virus type 2 infection increases risk of HIV acquisition among women in Uganda and Zimbabwe	Multiple regions	HSV 2 seroprevalence was 52% in Uganda and 53% in Zimbabwe. The Seroincidence during follow up was 9.6 and 8.8/100 person years in Uganda and Zimbabwe, respectively.	AIDS
Buvé, A , Caraël, M , Hayes, R. J , Auvert, B , Ferry, B , Robinson, N. J , Anagonou, S , Kanhonou, L , Laourou, M , Abega, S ,Akam, E , Zekeng, L , Chege, J , Kahindo, M , Rutenberg, N , Kaona, F , Musonda, R.<br>Sukwa, T , Morison, L , Weiss, H. A , Laga, M.	2001	The multicentre study on factors determining the differential spread of HIV in four African cities: summary and conclusions	Multiple regions	Cotonous (29%), Yaounde (50%), Kisumu (67.7 %), Ndola (55.%) HIGHER IN WOMEN	AIDS
Caldeira, T. D. M , Gonçalves, C. V , Oliveira, G. R. de , Fonseca, T. V. da , Gonçalves, R , Amaral, C. T. do , Hora, V. P. da , Martinez, A. M. B. de	2013	Prevalence of herpes simplex virus type 2 and risk factors associated with this infection in women in Southern Brazil	South America	The prevalence of HSV 2 was 15.6%.	Revista do Instituto de Medicina Tropical de São Paulo
Cárcamo, C. P ,Campos, P. E ,García, P. J ,Hughes, J. P ,Garnett, G. P ,Holmes, K. K.	2012	Prevalences of sexually transmitted infections in young adults and female sex workers in	South America	Overall prevalence of HSV2 was 13·5% in men, 13·6% in women, and 60·6% in FSWs	Lancet Infectious Diseases

		Peru: a national population based survey			
Celentano, DavidD , Mayer, KennethH , Pequegnat, Willo , Abdala, Nadia , Green, AnnetteM , Handsfield, H. Hunter Hartwell, TylerD , National Institute of Mental Health Collaborative	2010	Prevalence of Sexually Transmitted Diseases and Risk Behaviors from the NIMH Collaborative HIV/STD Prevention Trial	South America	China (M (6.1 %) F (9.6%)), India (M (8.6%) F(56.8%)), Peru (M (24.4%),F (43.5%)), Russia (M (1.4%) F(4%)), Zimbabwe (M (13.5%) F(41.2%))	International Journal of Sexual Health
Charpentier, C , Koyalta, D ,Ndinaromtan, M , Tchobkréo, B , Jenabian, M. A , Day, N , Si Mohamed, A , Weiss, H , Bélec, L.	2011	Distribution of HIV 1 and HSV 2 epidemics in Chad revealing HSV 2 hot spot in regions of high risk HIV spread	Central Africa	HSV 2 seroprevalences was 15.7% (CI95 = 2.9% 24.6%)	Journal of Infection in Developing Countries
Chawla, R , Bhalla, P , Bhalla, K , Singh, M. M ,Garg, S.	2008	Community based study on seroprevalence of herpes simplex virus type 2 infection in New Delhi	South Asia	The prevalence of HSV 2 was found to be 7 and 8.6% in men and women, respectively.	Indian Journal of Medical Microbiology
Chen, XiangSheng , Yin, YuePing , Chen, LeiPing , Yu, YanHua , Wei, WanHui , Nguyen Thi Thanh, Thuy , Smith, J. S.	2007	Herpes simplex virus 2 infection in women attending an antenatal clinic in Fuzhou, China	East Asia	HSV 2 seroprevalence was 10.8% (95% CI: 8.3% to 13.8%)	Sexually Transmitted Infections
Chen, XiangSheng, Yin, YuePing , Gong, XiangDong , Liang, GuoJun , Zhang, WenYing , Poumerol, G , Shi, MeiQin , Wu, ShouQiang , Zhang, GuoCheng	2006	Prevalence of sexually transmitted infections among long distance truck drivers in Tongling, China	East Asia	HSV 2 in 4.4%	International Journal of STD & AIDS

Chow, E. P. F ,Tucker, J. D , Wong, F. Y , Nehl, E. J , Wang, YanJie , Zhuang, Xun , Zhang, Lei	2014	Disparities and risks of sexually transmissible infections among men who have sex with men in China: a meta analysis and data synthesis	East Asia	The prevalence of HSV 2 was 10.6% (CL 6.2–17.6%)	PLoS ONE
Clark, J. L , Konda, K. A , Munayco, C. V , Pún, M , Lescano, A. G , Leon, S. R , Pajuelo, J , Suarez Ognio, L , Klausner, J. D , Coates, T. J ,Cáceres, C. F.	2008	Prevalence of HIV, herpes simplex virus 2, and syphilis in male sex partners of pregnant women in Peru	South America	Prevalence HSV 2 was 16.0% (95%CI 14.3%–17.8%)	BMC Public Health
Conde Glez, C , Lazcano Ponce, E , Rojas, R , DeAntonio, R , Romano Mazzotti, L , Cervantes, Y , Ortega Barria, E.	2013	Seroprevalences of varicella zoster virus, herpes simplex virus and cytomegalovirus in a cross sectional study in Mexico	Central America	HSV 2 seroprevalences was 9.9% (95% CI: 7.9−12.0). Female higher in men	Vaccine
Couture, M. C , Soto, J. C , Akom, E , Labbé, A. C , Joseph, G , Zunzunegui, M. V.	2008	Clients of female sex workers in Gonaives and St Marc, Haiti characteristics, sexually transmitted infection prevalence and risk factors	Caribbean	The prevalence of HSV 2 was 22%, P value = 0.001	Sexually Transmitted Diseases
Cowan, F. M , French, R. S , Mayaud, P , Gopal, R , Robinson, N. J , Oliveira, S. A. de , Faillace, T , Uusküla, A , Nygård Kibur, M , Ramalingam, S , Sridharan, G ,El Aouad, R , Alami, K , Rbai, M , Sunil Chandra, N. P , Brown, D. W.	2003	Seroepidemiological study of herpes simplex virus types 1 and 2 in Brazil, Estonia, India, Morocco, and Sri Lanka	Multiple regions	Barazil, ANC attenders (29.3%), Estonia (23.8%), India (7.9%), M arocco (12.9%%), Sri Lanka (8.3%) higher in women	Sexually Transmitted Infections

Daniels, B , Wand, H , Ramjee, G.	2016	Prevalence of Herpes Simplex Virus 2 (HSV 2) infection and associated risk factors in a cohort of HIV negative women in Durban, South Africa	Southren Africa	The HSV 2 prevalence at base line in this cohort of women was 65%. The HSV 2 incidence during study follow up was 22.3 per 100 PY (95% CI 20.4–24.3)	BMC Research Notes
Dave, S. S , Copas, A , Richens, J , White, R. G ,. Kosambiya, J. K , Desai, V. K , Stephenson, J. M.	2012	HIV and STI prevalence and determinants among male migrant workers in India	South Asia	Herpes simplex virus 2 w were 3.3 (2.0–5.4), 3.5 (2.1–5.9) , 3.4 (2.4–4.9) among different	PLoS ONE
Lai,W., Chen,C.Y., Morse,S. A., Fehler, H.G. , Liu, H., Ballard, R.C.	2003	Increasing relative prevalence of HSV 2 infection among men with genital ulcers from a mining community in South Africa	Southren Africa	M PCR detectedT pallidum, HSV, andH ducreyiin 10.3%, 17.2%, and 69.4% of 232 GUDpatients during 1993–4 and in 12.4%, 36.0%, and 50.5% of 186 GUD patients in 1998. The pro portion of patients with more than one agent increased significantly from 7.3% (17/232) in 1993–4to 16.7% (31/186) in 1998 (p <0.01). HSV 2 was detected in a higher proportion of ulcer specimensfrom HIV infected patients than in specimens from HIV uninfected patients during both time periods(1993–4: 26.2%v6.7%, p <0.001;	Sexually Transmitted Infections

Nahal B, *et al. BMJ Glob Health* 2024; 9:e012719. doi: 10.1136/bmjgh-2023-012719

				1998: 42.1%v29.6%, p >0.09).	
Davies, Stephen C, Taylor, Janette A , Sedyaningsih Mamahit, Endang R , Gunawan, Suriadi , Cunningham, Anthony L , Mindel, Adrian	2007	Prevalence and risk factors for herpes simplex virus type 2 antibodies among low and high risk populations in Indonesia	East Asia	HSV 2 antibodies were detected in 153 of 176 FSWs (86.9%; 95% confidence interval [CI], 81.0–91.5); among nonsex worker females, HSV 2antibodies were detected in 78 of 418 (18.7%; 95% CI, 15.0–22.7)	Sexually Transmitted Diseases
Debrah, Oksana , Agyemang Yeboah, Francis , Asmah, Richard Harry , Timmy Donkoh, Emmanuel ,Seini, Mohammed Mustapha , Fondjo, Linda Ahenkorah , Sight, Nilok , Owusu Dabo, Ellis	2018	SERO prevalence of herpes simplex virus type 1 and type 2 among women attending routine Cervicare clinics in Ghana	West Africa	The HSV 2 seroprevalence estimates was 78.4% (95% CI:74.5–81.8%)	BMC Infection disease
Desta, Kassa , Gebremedhin, Gebremichael , Tesfaye, Tilahun , Abenezer, Ayalkebet , Yemane, Abrha , Getnet, Mesfin , Yohannes, Belay , Minilik, Demissie , Atsbeha, Gebrexiabher , Yibeltal, Assefa	2018	Prevalence of sexually transmitted infections (HIV, hepatitis B virus, herpes simplex virus type 2, and syphilis) in pregnant women in Ethiopia: trends over 10 years (2005 2014)	East Africa	The prevalence of HSV 2 range between (47.5% to 28.5%)	International Journal of Infectious Diseases

Dipankar, Biswas , Biswajyoti, Borkakoty , Jagadish, Mahanta , Kamini, Walia , Lahari, Saikia , Akoijam, B. S , Lobsang, Jampa , Alia, Kharkonggor , Eric, Zomawia	2011	Seroprevalence and risk factors of herpes simplex virus type 2 infection among pregnant women in Northeast India	South Asia	seroprevalence of HSV 2 infection was 8.7% (95% CI 7.3 10.0)	BMC Infection disease
Domercant, J. W, Louis, F. J , Hulland, E , Griswold, M , Andre Alboth, J , Ye, Tun , Marston, B. J.	2017	Seroprevalence of Herpes Simplex Virus type 2 (HSV 2) among pregnant women who participated in a national HIV surveillance activity in Haiti	Caribbean	The prevalence of HSV 2 was 31.4% (95% CI: 27.7–35.4)	BMC Infection disease
Drisu, U. I, Oronsaye, F. E , Adejumo, B. I. G ,Uchuno, G. A , Emmanuel, A. M , Dimkpa, U , Omosor, K. I , Adebowale, M. O , Abdulrahman, O. N , Ukatu, E. N.	2018	Seroprevalence, type specific of herpes simplex virus and associated risk factors among women of child bearing age in Kogi state, Nigeria	West Africa	The prevalence of HSV was 76.7%.	Health
Duan, S , Ding, Y , Wu, Z , Rou, K , Yang, Y , Wang, J , Gao, M , Ye, R , Xiang, L , He, N.	2016	The prevalence of HSV 2 infection in HIV 1 discordant couples	East Asia	The prevalence of HSV was 42·4%	Epidemiology and Infection
Eis Hübinger, A. M , Nyankiye, E , Bitoungui, D. M , Ndjomou, J.	2002	Prevalence of herpes simplex virus type 2 antibody in Cameroon	Central Africa	the HSV 2 seroprevalence was 70.0%.	Sexually Transmitted Diseases
Feng, Y , Wu, ZunYou , Detels, R , Qin, GuangMing , Liu, Li , Wang, XiaoDong , Wang, Jun , Zhang, LingLin	2010	HIV/STD prevalence among men who have sex with men in Chengdu, China and associated risk factors for HIV infection	East Asia	HSV 2 prevalence was , 24.7%	JAIDS, Journal of Acquired Immune Deficiency Syndromes

Nahal B, *et al. BMJ Glob Health* 2024; 9:e012719. doi: 10.1136/bmjgh-2023-012719

Finger Jardim, F , Avila, E. C , Hora, V. P. da , Gonçalves, C. V , Martinez, A. M. B. de , Soares, M. A.	2017	Prevalence of herpes simplex virus types 1 and 2 at maternal and fetal sides of the placenta in asymptomatic pregnant women	South America	The prevalence of HSV -2 was 12.6% (maternal side) and 8.3% (fetal side).	American Journal of Reproductive Immunology
Francis, Suzanna C , Mthiyane, T. Nondumiso , Baisley, Kathy , McHunu, S. Lerato , Ferguson, Jane B , Smit, Theresa , Crucitti, Tania , Gareta, Dickman , Dlamini, Siphephelo , Mutevedzi, Tinofa , Seeley, Janet , Pillay, Deenan , McGrath, Nuala , Shahmanesh, Maryam	2018	Prevalence of sexually transmitted infections among young people in South Africa: A nested survey in a health and demographic surveillance site	Southren Africa	the prevalence of HSV 2 among men and women, respectively, was 16.8% and 28.7%	PLoS ONE
Gabster, A , Pascale, J. M , Cislaghi, B , Francis, S. C , Weiss, H. A , Martinez, A , Ortiz, A , Herrera, M , Herrera, G , Gantes, C. , Quiel, Y , Ríos, A , Campbell, E , Mayaud, P.	2019	High prevalence of sexually transmitted infections, and high risk sexual behaviors among indigenous adolescents of the Comarca Ngäbe Buglé, Panama	Central America	in HSV 2 seroprevalence (females, 16.1%; males, 16.1%;P= 0.99).	Sexually Transmitted Diseases
Gibney, L , Saquib, N ,Macaluso, M , Hasan, K. N , Aziz, M. M , Khan, A. Y. M. H ,Choudhury, P.	2002	STD in Bangladesh's trucking industry: prevalence and risk factors	South Asia	The prevalence of HSV 2 was (25.8%)	Sexually Transmitted Infections
Gutierrez, J. P , Bertozzi, S. M , Conde Glez, C. J , Sanchez Aleman, M. A.	2006	Risk behaviors of 15 21 year olds in Mexico lead to a high prevalence of sexually transmitted infections: results of a survey in disadvantaged urban areas	Central America	The prevalence of HSV 2 was 12%	BMC Public Health

Gutierrez, J. P , Conde González, C. J , Walker, D. M , Bertozzi, S. M.	2007	Herpes simplex virus type 2 among Mexican high school adolescents: prevalence and association with community characteristics	Central America	The seropositivity of HSV 2 was 21%	Elsevier
Halton, K , Ratcliffe, A. A , Morison, L , West, B , Shaw, M , Bailey, R , Walraven, G.	2003	Herpes simplex 2 risk among women in a polygynous setting in rural West Africa	West Africa	HSV2 prevalence was 16% amongst never married women and 36% amongst ever married women	AIDS
Hazel, A , Foxman, B , Low, B. S.	2015	Herpes simplex virus type 2 among mobile pastoralists in northwestern Namibia	Southren Africa	The overall prevalence of HSV 2 was 35%	Annals of Human Biology
Hoa Van, Le , Schoenbach, V. J , Herrero, R , Anh Thi Hoang, Pham , Hieu Trong, Nguyen , Thuy Thi, Nguyen , Muñoz, N , Franceschi, S , Vaccarella, S , Parkin, M. D , Snijders, P. J. F , Morrow, R. A. , Smith, J. S.	2009	Herpes simplex virus type 2 seropositivity among ever married women in South and North Vietnam: a population based study	East Asia	HSV 2 seroprevalence was (30.8%, 95% CI: 28.1–33.4)	Sexually Transmitted Diseases
Holtz, T. H , Thienkrua, W , McNicholl, J. M , Wimonsate, W , Chaikummao, S , Chonwattana, W , Wasinrapee, P , Varangrat, A , Mock, P. A , Sirivongrangson, P , Griensven, F. van	2012	Prevalence of Treponema pallidum seropositivity and herpes simplex virus type 2 infection in a cohort of men who have sex with men, Bangkok, Thailand, 2006 2010	East Asia	HSV 2 seropositive rates was 20.7%	International Journal of STD & AIDS

Hu, QingHai , Xu, JunJie , Chu, ZhenXing , Zhang, Jing , Yu, YanQiu , Yu, Huan , Ding, HaiBo , Jiang, YongJun , Geng, WenQing , Wang, Ning , Shang, Hong	2017	Prevalence and determinants of herpes simplex virus type 2 (HSV 2)/syphilis co infection and HSV 2 mono infection among human immunodeficiency virus positive men who have sex with men: a cross sectional study in northeast China	East Asia	The prevalence of HSV 2 was 48.6% (95% CL 44.4–52.8%)	Japanese Journal of Infectious Diseases
Huai, PengCheng , Li, FuRong , Li, Zhen , Sun, LeLe , Fu, Xi'an , Pan, Qing , Yu, GongQi , Chai, ZeMin , Chu, TongSheng , Mi, ZiHao , Bao, FangFang , Wang, HongLei , Zhou, BingNi , Wang, Chuan , Sun, YongHu , Niu, GuiYe , Zhang, Yuan , Fu, FangHui , Lang, XiaoQiao , Wang, XiaoLing , Zhao, Hui , Liu, DaiNa , Liu, Hong , Liu, DianChang , Liu, Jian , Xu, AiQiang	2019	Seroprevalence and associated factors of HSV 2 infection among general population in Shandong Province, China	East Asia	The seroprevalence of HSV 2 infection was 4.2% (95% confidence interval [CI], 3.2–5.3) in females, and in males 2.7% (95% CI, 1.1–4.2)	BMC Infection disease
Ibrahim, A , Adamu, I. A , Haruna, A.	2015	Seroepidemiology of Herpes Simplex Virus Type 2 (HSV 2) among incarcerated population of Potiskum medium security prison Potiskum Yobe State: study of prevalence and associated risk factors	West Africa	The seropositive for HSV 2 was 14.5%	International Journal of Current Microbiology and Applied Sciences

Janbakhash, A , Mansouri, F , Vaziri, S , Sayad, B , Afsharian, M , Abedanpor, A.	2012	Seroepidemiology of herpes simplex virus type 2 (HSV2) in HIV infected patients in Kermanshah Iran	South Asia	Out of 170 cases, 11 were seropositive for HSV2 (6.5%) in case group and 2 of 165 (1.21%) in control group (p=0.015).	Caspian Journal of Internal Medicine (CJIM)
Kapiga, S. H , Sam, N. E , Shao, J. F , Masenga, E. J , Renjifo, B , Kiwelu, I. E , Manongi, R , Fawzi, W. W ,Essex, M.	2003	Herpes simplex virus type 2 infection among bar and hotel workers in Northern Tanzania: prevalence and risk factors	East Africa	The seroprevalence of HSV 2 was 43.5%.	Sexually Transmitted Diseases
Karad, A. B , Khade, S. L.	2013	Seroepidemiological study of herpes simplex virus type 2 infection in HIV positive patients	South Asia	Seroprevalence of HSV 2 was 48.4%	International Journal of Medicine and Public Health
Karim, Q. A , Kharsany, A. B. M , Leask, K , Ntombela, F , Humphries, H , Frohlich, J. A , Samsunder, N , Grobler, A , Dellar, R , Karim, S. S. A.	2014	Prevalence of HIV, HSV 2 and pregnancy among high school students in rural KwaZulu Natal, South Africa: a bio behavioural cross sectional survey	Southren Africa	HSV 2 prevalence was 2.6% (95% CI 1.6 to 3.7) in males and 10.7% (95% CI 8.8 to 12.6) in females (p<0.001).	Sexually Transmitted Infections
Kirakoya Samadoulougou, F , Nagot, N , Defer, M. C ,Yaro, S , Fao, P , Ilboudo, F , Langani, Y , Meda, N , Robert, A.	2011	Epidemiology of herpes simplex virus type 2 infection in rural and urban Burkina Faso	West Africa	The prevalence of HSV 2 among pregnant women was 18.0% (95% (CI) 16.3% 19.7%), versus semiurban areas (21.4%; CI: (17.2% 26.2%) (P = 0.08), whereas the prevalence of HSV 2 was 23.7% (CI: 20.9% 26.6%).	Sexually Transmitted Diseases

Konda, Kelika A , Klausner, Jeffrey D , Lescano, Andres G , Leon, Segundo , Jones, Franca R , Pajuelo, Jose , Caceres, Carlos F , Coates, Thomas J.	2005	The epidemiology of herpes simplex virus type 2 infection in low income urban populations in coastal Peru	South America	In the socially marginalized population, HSV 2 prevalencewas 72.3% . In the general population, HSV 2 preva lence was 20.5% in women and 7.1% in men.	Sexually Transmitted Diseases
Kularatne, R. S , Muller, E. E , Maseko, D. V , Kufa Chakezha, T ,Lewis, D. A.	2018	Trends in the relative prevalence of genital ulcer disease pathogens and association with HIV infection in Johannesburg, South Africa, 2007 2015	Southren Africa	Seroprevalence of HSV 2 was 65.2%	PLoS ONE
Li, JiaMin , Chen, YongRu , Li, XiTao , Xu, WenCan	2011	Screening of Herpes simplex virus 2 infection among pregnant women in southern China	East Asia	The prevalence of HSV 2 was 23.56% (95% [CI] = 21.53–26.00)	Journal of Dermatology
Looker, Katharine J , Magaret, Amalia S , Turner, Katherine M. E , Vickerman, Peter , Gottlieb, Sami L , Newman, Lori M.	2015	Global estimates of prevalent and incident herpes simplex virus type 2 infections in 2012	Multiple regions	The prevalence of HSV 2 world wide was 11.3%	PLoS ONE
Lowe, S , Mudzviti, T , Mandiriri, A , Shamu, T , Mudhokwani, P , Chimbetete, C , Luethy, R , Pascoe, M.	2019	Sexually transmitted infections, the silent partner in HIV infected women in Zimbabwe	Southren Africa	HSV 2 prevalence 52.5%	Southern African Journal of HIV Medicine
Luther, K. M. M , Rodrigue, D. N. R , Annie, N. N. R , Jean, A. A , Michel, W , Elisée, E , Henri, A. Z. P.	2015	Seroprevalence and factors associated with herpes simplex virus type 2 and Chlamydia trachomatis among women	Central Africa	.The prevalence of HSV 2 n HIV positive patients was 73.7% versus 81.8% in HIV negative individuals.	British Journal of Medicine and Medical Research

Nahal B, *et al. BMJ Glob Health* 2024; 9:e012719. doi: 10.1136/bmjgh-2023-012719

		attending AIDS care unit at the Bonassama district hospital			
Madebe, R , Kiwelu, I , Ndaro, A , Francis, F , Baraka, V , Theilgaard, Z ,Katzenstein, T.	2020	Herpes Simplex virus type 2 seroprevalence and risk factors among adolescents and youth with HIV 1 in Northern, Tanzania	East Africa	The HSV 2 seroprevalence was 18%	Journal of Infection in Developing Countries
Malary, M , Abedi, G , Hamzehgardeshi, Z , Afshari, M , Moosazadeh, M.	2016	The prevalence of herpes simplex virus type 1 and 2 infection in Iran: a meta analysis	South Asia	The total prevalences of HSV 2 was 6.5% (4.7 8.2)	International Journal of Reproductive BioMedicine
Meque, I , Dubé, K , Feldblum, P. J , Clements, A. C. A , Zango, A , Cumbe, F , Chen, P. L , Ferro, J. J , Wijgert, J. H. van de	2014	Prevalence, incidence and determinants of herpes simplex virus type 2 infection among HIV seronegative women at high risk of HIV infection: a prospective study in Beira, Mozambique	Southren Africa	The HSV 2 prevalence at baseline was 60.6% (95% CI: 55.7% –65.4%).	PLoS ONE
Mmbaga, E. J , Moen, K , Makyao, N , Mpembeni, R , Leshabari, M. T.	2017	HIV and STI s among men who have sex with men in Dodoma municipality, Tanzania: a cross sectional study	East Africa	The seroprevalence of HSV 2 was 38.5%,	Sexually Transmitted Diseases
Mohammed, Qutub , Javed, Akhter	2003	Epidemiology of genital herpes (HSV 2) among brothel based female sex workers in Bangladesh	South Asia	a 33 (7.1%) samples were seropositive for HSV	European Journal of Epidemiology

Nahal B, *et al. BMJ Glob Health* 2024; 9:e012719. doi: 10.1136/bmjgh-2023-012719

Msuya, S. E , Mbizvo, E , Akhtar, Hussain , Sam, N. E , Jeansson, S , Stray Pedersen, B.	2003	Seroprevalence and correlates of herpes simplex virus type 2 among urban Tanzanian women	East Africa	The seroprevalence of HSV 2 was 39%	Sexually Transmitted Diseases
Mugo, N , Dadabhai, S. S , Bunnell, R , Williamson, J , Bennett, E , Baya, I , Akinyi, N , Mohamed, I , Kaiser, R.	2011	Prevalence of herpes simplex virus type 2 infection, human immunodeficiency virus/herpes simplex virus type 2 coinfection, and associated risk factors in a national, population based survey in Kenya	East Africa	HSV 2seroprevalence was 35.1%	Sexually Transmitted Diseases
Nakku Joloba, E , Kambugu, F , Wasubire, J , Kimeze, J , Salata, R , Albert, J. M , Rimm, A , Whalen, C.	2014	Sero prevalence of herpes simplex type 2 virus (HSV 2) and HIV infection in Kampala, Uganda	East Africa	The HSV 2 prevalence was 58% (95% CI: 55, 60)	African Health Sciences
Nakubulwa, S , Kaye, D. K , Bwanga, F , Tumwesigye, N. M , Nakku Joloba, E , Mirembe, F. M.	2016	Incidence and risk factors for herpes simplex virus type 2 seroconversion among pregnant women in Uganda: a prospective study	East Africa	Of 191 women, 15 (7.9%) seroconverted during pregnancy.	Journal of Infection in Developing Countries
Navadeh, S , Mirzazadeh, A , Mousavi, L , Haghdoost, A. A , Fahimfar, N , Sedaghat, A.	2012	HIV, HSV2 and syphilis prevalence in female sex workers in Kerman, South East Iran; using respondent driven sampling	South Asia	The prevalence of HSV2 was 18.0%	Iranian Journal of Public Health
Ngo, T. D , Laeyendecker, O , Li, C , Tai, H , Cui, M , Lai, S , Quinn, T. C.	2008	Herpes simplex virus type 2 infection among commercial sex workers in Kunming, Yunnan Province, China	East Asia	the HSV 2 prevalence was 33.0%	International Journal of STD & AIDS

Paz Bailey, G , Morales Miranda, S , Jacobson, J. O , Gupta, S. K , Sabin, K , Mendoza, S , Paredes, M , Alvarez, B ,Monterroso, E.	2009	High rates of STD and sexual risk behaviors among Garifunas in Honduras	Central America	The prevalences of HSV 2 was 51.1% (95% CI: 46.7 to 55.6)	JAIDS, Journal of Acquired Immune Deficiency Syndromes
O'Farrell, N , Nguyen Vu, Thuong , Khuu Van, Nghia , Luong Thu, Tram , Nguyen Thanh, Long	2006	HSV2 antibodies in female sex workers in Vietnam	East Asia	The prevalence of HSV 2 antibodies was 27.7% (95% confidence interval [CI]: 24.8–30.7%).	International Journal of STD & AIDS
Panchanadeswaran, S , Johnson, S. C , Mayer, K. H , Srikrishnan, A. K , Sivaran, S , Zelaya, C. E , Go, V. F , Solomon, S , Bentley, M. E , Celentano, D. D.	2006	Gender differences in the prevalence of sexually transmitted infections and genital symptoms in an urban setting in southern India	South Asia	The most commonly detected STI was HSV2; 13.2%	Sexually Transmitted Infections
Paz Bailey, G , Shah, N , Creswell, J , Guardado, M. E , Nieto, A. I , Estrada, M. C , Cedillos, R , Pascale, J. M , Monterroso, E.	2012	Risk behaviors and STI prevalence among people with HIV in El Salvador	Central America	The prevalence of HSV2 was 84.5 %	Open AIDS Journal
Reward, E. E , Muo, S. O , Orabueze, I. N. A , Ike, A. C.	2019	Seroprevalence of herpes simplex virus types 1 and 2 in Nigeria	West Africa	The seroprevalences for HSV 2 was 63.4% (56.1–70.4%).	Pathogens and global health
Rodrigues, J , Grinsztejn, B , Bastos, F. I , Velasque, L , Luz, P. M , Souza, C. T. V. de , Georg, I , Pilotto, J. H , Veloso, V. G.	2009	Seroprevalence and factors associated with herpes simplex virus type 2 among HIV negative high risk men who have sex with men from Rio de Janeiro, Brazil: a cross sectional study	South America	Seroprevalence of HSV 2 was of 45.7%	BMC Infection disease

Rodríguez, A. C , Castle, P. E , Smith, J. S , Bratti, C , Hildesheim, A , Schiffman, M Viscidi, R , Burk, R. D , Ashley, R. L , Castellsagué, X , Herrero, R.	2003	A population based study of herpes simplex virus 2 seroprevalence in rural Costa Rica	Central America	Overall age adjusted HSV 2 seroprevalence was 38.5% (95% CI, 37.5 to 39.5)	Sexually Transmitted Infections
Sanchez Aleman, M. A , Villar Tapia, Y. G. del , Gutierrez, J. P , Garcia Cisneros, S , Olamendi Portugal, M. L , Herrera Ortiz, A , Velazquez Meza, M , Conde Glez, C. J.	2018	Heterogeneity of herpes simplex virus type 2 seroprevalence from a national probability survey in Mexico, 2012	Central America	National HSV 2 seroprevalence was 9.9%, 2.4% among adolescents and 11.7% among adults with HSV 2 seroprevalence heterogeneous across the country.	Sexually Transmitted Diseases
Saramma, Jacob , Thatchinamoorthy, Gopa , Sivasangeetha, Kanagasaba , Anitha, Durairaj , Kamala, Sushi , Ganesan, Arumugam	2015	Herpes simplex virus 2 infection in HIV seropositive individuals in Tamil Nadu, India	South Asia	The overall prevalence of HSV 2 was 50%	International Journal of Medicine and Public Health
Sgaier, S. K , Mony, P , Jayakumar, S , McLaughlin, C , Arora, P , Kumar, R , Bhatia, P , Jha, P.	2011	Prevalence and correlates of Herpes Simplex Virus 2 and syphilis infections in the general population in India	South Asia	The prevalence of HSV 2 was 10.1%.	Sexually Transmitted Diseases
Smith, Jennifer S , Robinson, N. Jamie	2002	Age specific prevalence of infection with herpes simplex virus types 2 and 1: a global review	Multiple regions	HSV 2 prevalnce was in the late 1970s (16.0%) and early 1990s (20.8%)	The Journal of infectious diseases

Nahal B, *et al. BMJ Glob Health* 2024; 9:e012719. doi: 10.1136/bmjgh-2023-012719

Sudfeld, C. R , Hewett, P. C , Abuelezam, N. N , Chalasani, S , Soler Hampejsek, E , Kelly, C. A , Mensch, B. S.	2013	Herpes simplex virus type 2 cross sectional seroprevalence and the estimated rate of neonatal infections among a cohort of rural Malawian female adolescents	Southren Africa	11.2% (95% CI 7.4% to 15.9%) for 17 yr old, 15.7% (95% CI 11.9% to 20.2%) for 18 yr old, 17.8% (95% CI 13.4% to 22.9%) for 19 yr old, 18.6% (95% CI 11.9% to 27.0%) for 20 yr old	Sexually Transmitted Infections
Sutcliffe, S , Taha, T. E , Kumwenda, N. I , Taylor, E , Liomba, G. N.	2002	HIV 1 prevalence and herpes simplex virus 2, hepatitis C virus, and hepatitis B virus infections among male workers at a sugar estate in Malawi	Southren Africa	overall HSV 2 prevalence was 69.3 % where the prevalnce was 88.1 % among HIV positive and 64.3 % among negative subject	JAIDS, Journal of Acquired Immune Deficiency Syndromes
Tayyebi, D , Sharifi, S.	2010	Seroepidemiology of infection with herpes simplex virus types 1 and 2 (HSV1 and HSV2) among asymptomatic university students attending Islamic Azad University of Kazeroun, southwest of Iran	South Asia	HSV 2 prevalnce was 23.3%	Iranian Journal of Clinical Infectious Diseases
Theng, C. T. S , Sen, P. R , Chio, T. W. M , Tan, H. H , Wong, M. L , Chan, R. K. W.	2006	Seroprevalence of herpes simplex virus 1 and 2 in attendees of a sexually transmitted infection clinic in Singapore	East Asia	HSV 2 seroprevalnce (28.5%)	sexuall health
Theng, T. S. C , Sen, P. R , Tan, H. H , Wong, M. L , Chan, K. W. R.	2006	Seroprevalence of HSV 1 and 2 among sex workers attending a sexually transmitted infection clinic in Singapore	East Asia	HSV 2 seroprevalnce (79.0%)	International Journal of STD & AIDS

Torrone, E. A , Morrison, C. S , Chen, PaiLien , Kwok, C , Francis, S. C , Hayes, R. J , Looker, K. J , McCormack, S , McGrath, N , Wijgert, J. H. H. M. van d , Watson Jones, D , Low, N , Gottlieb, S. L.	2018	Prevalence of sexually transmitted infections and bacterial vaginosis among women in sub Saharan Africa: an individual participant data meta analysis of 18 HIV prevention studies	Multiple regions	1 HSV 2 prevalence was high among 15–24 year olds, in the South African clinic/community based populations (summary estimate: 39.3% [95%CI:34.3%,44.6%] ;I2= 88.8%), in Southern/Eastern African clinic/community based populations 46.8%[95%CI:38.2%,55.6%] , in the higher risk populations in Eastern Africa 56.3%[95%CI:49.2%,63.1%] . 2 HSV 2 was even higher among women aged 25–49 years, in the South African clinic/community based populations 77.8%(95%CI:75.6%,79.9%) , in Southern/Eastern African clinic/community based populations (70.0%[95%CI:61.8%,77.1%), in the higher risk populations in Eastern Africa (83.3%[95%CI:79.3%,86.6%).	PLoS Medicine

Vandenhoudt, H. M , Langat, L , Menten, J , Odongo, F , Oswago, S , Luttah, G , Zeh, C , Crucitti, T , Laserson, K , Vulule, J , Buve, A.	2007	HIV and other sexually transmitted infections among female sex workers in Kinshasa, Democratic Republic of Congo, in 2002	East Africa	The HSV 2 seroprevalence was 58.5%.	PLoS Medicine
Wang, HaiBo , Wang, Ning , Chen, R. Y , Sharp, G. B , Ma, YanLin , Wang, GuiXian , Ding, GuoWe , Wu, ZhengLai	2008	Prevalence and predictors of herpes simplex virus type 2 infection among female sex workers in Yunnan Province, China	East Asia	HSV 2 was the most common STI (68%)	International Journal of STD & AIDS
Wang, JunJie , Zhu, ZhiBin , Yang, Xi , Wu, Jing , Wang, HaiBo , Feng, Lin , Ding, GuoWei , Norris, J. L , Wang, Ning	2012	Herpes simplex virus type 2 risks in female sex workers in the China Vietnam border county of Hekou	East Asia	HSV 2 prevalence rates were 58.3%	Biomedical and Environmental Sciences
Yahya Malima, K. I , Evjen Olsen, B , Matee, M. I , Fylkesnes, K , Haarr, L.	2008	HIV 1, HSV 2 and syphilis among pregnant women in a rural area of Tanzania: prevalence and risk factors	East Africa	HSV 2 prevalence 20.7% (95% CI: 18.53–23.00)	BMC Infectious Diseases
Yang, Ying , Yao, JinJian , Gao, MeiYang , Su, HuaLin , Zhang, TieJun , He, Na	2011	Herpes simplex virus type 2 infection among female sex workers in Shanghai, China	East Asia	The overall HSV 2 seroprevalence was 47.3% (375/793)	AIDS Care
Yin, YuePing , Chen, ShaoChun , Wang, HongChun , Wei, WanHui , Wang, QianQiu , Liang, GuoJun , Jiang, Ning , Han, Yan , Chen, XiangSheng , Wang, BaoXi	2012	Prevalence and risk factors of HSV 2 infection and HSV 2/HIV coinfection in men who have sex with men in China: a multisite cross sectional study	East Asia	16.0% (95% confidence interval [CI], 14.2%–18.0%)	Sexually Transmitted Diseases

Vu, Lung , Misra, Kavita	2018	High Burden of HIV, Syphilis and HSV 2 and Factors Associated with HIV Infection Among Female Sex Workers in Tanzania: Implications for Early Treatment of HIV and Pre exposure Prophylaxis (PrEP)	East Africa	The prevalence of HSV 2 was 57.5 %	Journal of Infection in Developing Countries
Looker, Katharine J , Welton, Nicky J , Sabin, Keith M , Dalal, Shona , Vickerman, Peter , Turner, Katherine M. E , Boily, Marie Claude , Gottlieb, Sami L.	2020	Global and regional estimates of the contribution of herpes simplex virus type 2 infection to HIV incidence: a population attributable fraction analysis using published epidemiological data	Multiple regions	The prevalence of HSV 2 was 26·8% [19·7–34·5]	The Lancet Infectious Diseases
Paz Bailey, G , Ramaswamy, M , Hawkes, S. J , Geretti, A. M.	2006	Herpes simplex virus type 2: epidemiology and management options in developing countries	Multiple regions	HSV2 have been observed in sub-Saharan Africa, with age-adjusted prevalences in adults ranging from 30% to 80% in women and 10% to 50% in men. In South America, available data are mainly for women, in whom HSV2 prevalence ranges from 20% to 40%. Prevalence in the general population in Asian countries shows lower values, from 10% to 30%.6	Postgraduate medical journal

Nahal B, *et al. BMJ Glob Health* 2024; 9:e012719. doi: 10.1136/bmjgh-2023-012719

Madhivanan, Purnima , Krupp, Karl , Chandrasekaran, Varalakshmi , Karat, Chitra , Arun, Anjali , Klausner, Jeffrey D , Reingold, Arthur L.	2007	The Epidemiology of Herpes Simplex Virus Type 2 Infection Among Married Women in Mysore, India	South Asia	HSV 2 seropositive was 11.3% (95%CI: 9.4%–13.6%).	Sexually Transmitted Diseases
Patzi Churqui, Marianela , Terrazas Aranda, Katty , Liljeqvist, Jan Åke , Lindh, Magnus , Eriksson, Kristina	2020	Prevalence of viral sexually transmittedinfections and HPV high risk genotypes in women in rural communities in theDepartment of La Paz, Bolivia	South America	The seroprevalence of HSV 2 was 53%	BMC Infectious Diseases
Kalu, E. I , Ojide, C. K , Fowotade, A , Nwadike, V. U.	2014	Sexual behavioral correlates with HSV 2 seroprevalence among pregnant women in Nigeria	West Africa	Seroprevalence of HSV 2 antibody was 47.3%	Journal of Infection in Developing Countries
Gomes Naveca, Felipe , Sabidó, Meritxell , Amaral Pires De Almeida, Tatiana , Araújo Veras, Elaine , Contreras Mejía , Matilde Del Carmen , Galban, Enrique , Benzaken, Adele Schwartz	2013	Etiology of Genital Ulcer Disease in a Sexually Transmitted Infection Reference Center in Manaus,Brazilian Amazon	South America	HSV 2 was detected in 55.3% of GUD samples,	PLoS ONE
Noda, Angel A , Blanco, Orestes , Correa, Consuelo , Pérez, Lissette , Kourí, Vivian , Rodríguez, Islay	2016	Etiology of Genital Ulcer Disease in Male Patients Attending a Sexually Transmitted Diseases Clinic: First Assessment in Cuba	Caribbean	The prevalence of HSV 2 was 51.3%	Sexually Transmitted Diseases
Miranda, C. A. N , Lima, É G , Lima, D. B. S. de , Cobucci, R. N. O , Cornetta, M. da C. de M , Fernandes, T. A. A. de M , Azevedo, P. R. M. de , Azevedo, J.	2014	Genital Infection with Herpes Simplex Virus Types 1 and 2 in Women from Natal, Brazil	South America	For HSV 2, these rates were 12.3% for pregnant women and 15.5% non pregnant women	ISRN Obstetrics and Gynecology

C. V. de , Araújo, J. M. G. de , Fernandes, J. V.					

HSV 2 Diagnostics

Authors	Year	Title	Geographical area of study population (according to UN classification)	Main findings/Prevalence	Journal
Gopal, R. , Gibbs, T., Slomka, M. J. , Whitworth, J. , Carpenter, L. M. , Vyse, A. , Brown, D. W. G.	2000	A monoclonal blocking EIA for herpes simplex virus type 2 antibody: validation for seroepidemiological studies in Africa	east africa	suitable test for epidemiological studies and type specific management, comparable sensetivity and specificity to western blot	Journal of Virological Methods
Haripriya, V. , Rajendran, P. , Thyagarajan, S. P. , Sripathy, M. S. , Parthiban, R. , Ram, C. C. M. , Gopalakrishnan, P. B. , Chandraseskhar, G. S. , Melani, Rajendran	2002	A study on the comparison between clinical and microbiological diagnoses of sexually transmitted diseases	South Asia	laboratory/microbiological shows better accuracy	Journal of the Indian Medical Association
Hogrefe, W., Su, X. , Song, J. , Ashley, R. , Kong, L.	2002	Detection of herpes simplex virus type 2 specific immunoglobulin G antibodies in African sera by using recombinant gG2, Western blotting, and gG2 inhibition	east africa and southern africa	comparable sensetivity and specificity	Journal of Clinical Microbiology
Liu, H., Detels, R., Yin, Y., Li, X., Visscher, B.	2003	Do STD clinics correctly diagnose STDs? An assessment of STD management in Hefei, China	east asia	poor accuracy of diagnostics HSV overdignosed	International Journal of STD & AIDS
Ndjoyi Mbiguino, A., Ozouaki, F. , Legoff, J. , Mbopi Kéou, F. X.	2003	Comparison of washing and swabbing procedures for	central africa	cervicovaginal lavage/washing detects	Journal of Clinical Microbiology

Nahal B, *et al. BMJ Glob Health* 2024; 9:e012719. doi: 10.1136/bmjgh-2023-012719

, Si Mohamed, A. , Onas, I. N. , Avoune, E. , Bélec, L.		collecting genital fluids to assess cervicovaginal shedding of herpes simplex virus type 2 DNA		more than endocervicovaginal swabbing	
Wang, QianQiu , Yang, Ping , Zhong, MingYing , Wang, GuangJu	2003	Validation of diagnostic algorithms for syndromic management of sexually transmitted diseases	east asia	syndromic approach not as specific and sensitive for diagnosing GUD (inc HSV 2) as for discharge STDs	Chinese Medical Journal (Beijing)
Dyck, E. van, Buvé, A. , Weiss, H. A. , Glynn, J. R. , Brown, D. W. G. , Deken, B. de , Parry, J. , Hayes, R. J.	2004	Performance of commercially available enzyme immunoassays for detection of antibodies against herpes simplex virus type 2 in African populations	east, central and west africa (sub saharan africa)	best 3 kits for african (sensitivity and specificity) sera were Gull Premier, Biokit and kalon biological. Herpeselect good in industrialsed countries but not africa, cannot assume results will be comparabl in different areas ofo the world.	Journal of Clinical Microbiology
Kara, P., Meric, B., Zeytinoglu, A., Ozsoz, M.	2004	Electrochemical DNA biosensor for the detection and discrimination of herpes simplex type I and type II viruses from PCR amplified real samples	western asia (turkey)	successful discrimination and detection, rapid and low cost, safer (no ethidium bromide)	Analytica Chimica Acta
Laeyendecker, O., Henson, C. , Gray, R. H. , Nguyen, R. H. N. , Horne, B. J. , Wawer, M. J. , Serwadda, D. , Kiwanuka, N. , Morrow, R. A. , Hogrefe, W. , Quinn, T. C.	2004	Performance of a commercial, type specific enzyme linked immunosorbent assay for detection of herpes simplex virus type 2 specific antibodies in Ugandans	east africa	HIV status did not effect performance of herpselect and does not explain different performance in different geo areas , difference may be due to different strains and gG2 epitope variations	Journal of Clinical Microbiology
Hoyo, C. , Hoffman, I. , Moser, B. K. , Hobbs, M. M. , Kazembe, P. , Krysiak, R. G. , Cohen, M. S.	2005	Improving the accuracy of syndromic diagnosis of genital ulcer disease in Malawi	southern africa	syndromic algorythm does not need to be weighted	Sexually Transmitted Diseases

				no longer therefore easier to use	
Nagot, N. , Foulongne, V. , Becquart, P. , Mayaud, P. , Konate, I. , Ouedraogo, A. , Defer, M. C. , Weiss, H. , Perre, P. van de , Segondy, M.	2005	Longitudinal assessment of HIV 1 and HSV 2 shedding in the genital tract of West African women	west africa	eCVL is reliable method for HSV 2 detection, could be useful in future trials with hiv and hsv positivity as an outcome	JAIDS, Journal of Acquired Immune Deficiency Syndromes
Görander, Staffan , Mbwana, Judica , Lyamuya, Eligius , Lagergård, Teresa , Liljeqvist, Jan Ake	2006	Mature glycoprotein g presents high performance in diagnosing herpes simplex virus type 2 infection in sera of different tanzanian cohorts	east africa	all similar performance on high prevalence group but mgG2 ELISA performed best overall (accuracy) and most suitable for future studies and clinical practice. Higher sensetivity of hereselect in GUD pts that blood donors	Clinical and vaccine immunology : CVI
Legoff, Jérôme , Bouhlal, Hicham , Grésenguet, Gérard , Weiss, Helen , Khonde, Nzambi , Hocini, Hakim , Désiré, Nathalie , Si Mohamed, Ali , de Dieu Longo, Jean , Chemin, Cécile , Frost, Eric , Pépin, Jacques , Malkin, Jean Elie , Mayaud, Philippe , Bélec, Laurent	2006	Real time PCR quantification of genital shedding of herpes simplex virus (HSV) and human immunodeficiency virus (HIV) in women coinfected with HSV and HIV	west africa and central africa	real time PCR is useful and accurate for hsv 2 and is an affordable option for detecting shedding in future intervention studies	Journal of clinical microbiology
Li, Ding , Yang, Hao , Zhang, Wen Hong , Pan, Hao , Wen, Dong Qing , Han, Feng Chan , Guo, Hui Fang , Wang, Xiao Ming , Yan, Xiao Jun	2006	A simple parallel analytical method of prenatal screening	east asia	no sig difference between microarray and ELISA , microarray cheaper and easier therefore more suitable for mass prenatal screening, however early	Gynecologic and obstetric investigation

Nahal B, *et al. BMJ Glob Health* 2024; 9:e012719. doi: 10.1136/bmjgh-2023-012719

				days and many problems need resolving	
Nascimento, M. C. , Ferreira, S. , Sabino, E. , Hamilton, I. , Parry, J. , Pannuti, C. S. , Mayaud, P.	2007	Performance of the HerpeSelect (Focus) and Kalon enzyme linked immunosorbent assays for detection of antibodies against herpes simplex virus type 2 by use of monoclonal antibody blocking enzyme immunoassay and clinicovirological reference standards in Brazil	South America	Kalon performed better than herpeselect in Brazil	Journal of Clinical Microbiology
Gamiel, J. L. , Tobian, A. A. R. , Laeyendecker, O. B. , Reynolds, S. J. , Morrow, R. A. , Serwadda, D. , Gray, R. H. , Quinn, T. C.	2008	Improved performance of enzyme linked immunosorbent assays and the effect of human immunodeficiency virus coinfection on the serologic detection of herpes simplex virus type 2 in Rakai, Uganda	east africa	kalon perfomed best in uganda, higher than standard cutoffs were requird to achieve optimum sensetivity and specificity though. Biokit point of care test had low specificity (56.1%). None of the assays affected by HIV status	Clinical and Vaccine Immunology
LeGoff, J. , Mayaud, P. , Gresenguet, G. , Weiss, H. A. , Nzambi, K. , Frost, E. , Pepin, J. , Belec, L.	2008	Performance of HerpeSelect and Kalon assays in detection of antibodies to herpes simplex virus type 2	west africa and central africa	Hereselect detects seroconversion more frequently and earlier than kalon. Hereselect more sensetive at detecting nonprimary first episodes than kalon. Early infection more difficult to detect with both assays and this may affect the true sensetivities of herepeselect on studies. HIV status did not affect	Journal of Clinical Microbiology

				performanceof either assay	
Ngo, T. D. , Laeyendecker, O. , La, H. , Hogrefe, W. , Morrow, R. A. , Quinn, T. C.	2008	Use of commercial enzyme immunoassays to detect antibodies to the herpes simplex virus type 2 glycoprotein G in a low risk population in Hanoi, Vietnam	east asia	retesting herpeselect with biokit or kalon can significantly reduce % of false positive results	Clinical and Vaccine Immunology
Ngo, T. D. , Laeyendecker, O. , Morrow, R. A. , Lai, S. H. , Quinn, T. C.	2008	Comparison of three commercial immunoassays for detection of herpes simplex virus type 2 antibodies in commercial sex workers in Yunnan Province, China	east asia	herpeselect perfrmed the best very close to kalon, improved understanding of the performance of these assays in chinese sex workers	Clinical and Vaccine Immunology
Sen, P. , Sun, Y. J. , Tan, H. H. , Tan, S. H. , Chan, R.	2008	Comparison of nested polymerase chain reaction and virus culture for the diagnosis of genital herpes simplex virus infection	east asia	nested PCR much more accurate than culture. Can detect and type hsv th sufficient accuracy	
Yin, YuePing , Wu, ZunYou , Lin, C. Q. , Guan, JiHui , Wen, Y. , Li, L. , Detels, R. , Rotheram Borus, M. J.	2008	Syndromic and laboratory diagnosis of sexually transmitted infection: a comparative study in China	east asia	syndromic approach sensitivity was approximately 10%, syndromic approach not good for asymptomatic	International Journal of STD & AIDS
Shilpee, Choudhry , Ramachandran, V. G. , Shukla, Das , Bhattacharya, S. N. , Mogha, N. S.	2009	Serological profile of HSV 2 in patients attending STI clinic: evaluation of diagnostic utility of HSV 2 IgM detection	South Asia	serum IgM not valuable as a single test for genital herpes diagnosis, would only be used as a supportive method. It isnt useful in determining whether an infection was recently acquired	Indian Journal of Pathology & Microbiology
Smith, J. S. , Bailey, R. C. , Westreich, D. J. , Maclean, I. , Agot, K. , Ndinya Achola, J. O. ,	2009	Herpes simplex virus type 2 antibody detection performance in Kisumu, Kenya, using the	east africa	kalon performed better than herpeselect in young men in kenya	Sexually Transmitted Infections

Hogrefe, W. , Morrow, R. A. , Moses, S.		Herpeselect ELISA, Kalon ELISA, Western blot and inhibition testing			
Suntoke, T. R. , Hardick, A. , Tobian, A. A. R. , Mpoza, B. , Laeyendecker, O. , Serwadda, D. , Opendi, P. , Gaydos, C. A. , Gray, R. H. , Wawer, M. J. , Quinn, T. C. , Reynolds, S. J.	2009	Evaluation of multiplex real time PCR for detection of Haemophilus ducreyi, Treponema pallidum, herpes simplex virus type 1 and 2 in the diagnosis of genital ulcer disease in the Rakai District, Uganda	east africa	thisnmultiplex real time PCR is a sensetive and specific, rapid and reproducible way of detecting aetiology of GUD in resource poor areas, better performance than previous PCR studies in uganda	Sexually Transmitted Infections
Delany Moretlwe, S. , Jentsch, U. , Weiss, H. , Moyes, J. , Ashley Morrow, R. , Stevens, W. , Mayaud, P.	2010	Comparison of focus HerpesSelect and Kalon HSV 2 gG2 ELISA serological assays to detect herpes simplex virus type 2 antibodies in a South African population	southern africa	both these ELISA tests had low specificity in this south african population. Increasing inidec value of herpeselect fro 1.1 to 3.5 improved specificity. HIV seropositivity reduced herpeselect specificity by 30%. Alternative algorythm with improved sec and sensi is 2 tests (hepeselect then confirm with kalon.	Sexually Transmitted Infections
Lingappa, J. , Nakku Joloba, E. , Magaret, A. , Friedrich, D. , Dragavon, J. , Kambugu, F. , Joloba, M. , Whalen, C. , Coombs, R. , Celum, C. , Morrow, R. A.	2010	Sensitivity and specificity of herpes simplex virus 2 serological assays among HIV infected and uninfected urban Ugandans	east africa	best performance is by kalon alone or hereselect followed by biokit confirmatory testing. Had to increase cutoff index of herpeselect to achieve better specificity. HIV positivity decreased herepeselect specificity.	International Journal of STD & AIDS

Nahal B, et al. BMJ Glob Health 2024; 9:e012719. doi: 10.1136/bmjgh-2023-012719

				Need more robust assays for HSV 2 and HIV public health activities in africa in point of care style	
Ng'ayo, M. O. , Friedrich, D. , Holmes, K. K. , Bukusi, E. , Morrow, R. A.	2010	Performance of HSV 2 type specific serological tests in men in Kenya	east africa	best performance was kalon using higher 1.5 cuoff. None of them perfect and need caution in interpretation. None of the rapid test suitable allone or as confirmatory test. Gen 2 no better than gen 1.	Journal of Virological Methods
Shilpee, Choudhry , Ramachandran, V. G. , Shukla, Das , Bhattacharya, S. N. , Mogha, N. S.	2010	Pattern of sexually transmitted infections and performance of syndromic management against etiological diagnosis in patients attending the sexually transmitted infection clinic of a tertiary care hospital	south asia	syndromic diagnosis 83% sensetive for HSV2 . Syndromic has its disadvantages and needs tobe regukarlyy reviewed and improved	Indian Journal of Sexually Transmitted Diseases and AIDS
LeGoff, Jérôme , Grésenguet, Gérard , Gody, Chrysostome , Longo, Jean De Dieu , Khonde, Nzambi , Weiss, Helen A. , Mayaud, Philippe , Bélec, Laurent	2011	Performance of the BioPlex 2200 multiplexing immunoassay platform for the detection of herpes simplex virus type 2 specific antibodies in African settings	west africa and central africa (CAR and Ghana)	excellent agreement between bioplex and hereselect at all cutoffs. Bioples has advantage of detecting hsv 1 and 2 so should be considered for future cliical and epidemiological uses	Clinical and vaccine immunology : CVI
Muvunyi, C. M. , Dhont, N. , Verhelst, R. , Crucitti, T. , Reijans, M. , Mulders, B. , Simons, G. , Temmerman, M. , Claeys, G. , Padalko, E.	2011	Evaluation of a new multiplex polymerase chain reaction assay STDFinder for the simultaneous detection of 7 sexually transmitted disease pathogens	east africa	STD Finder multples PCR performs as well as single/mono PCR assays. Multiplexing reduced cost	Diagnostic Microbiology and Infectious Disease

Nahal B, *et al. BMJ Glob Health* 2024; 9:e012719. doi: 10.1136/bmjgh-2023-012719

Neal, J. D. , Tobian, A. A. , Laeyendecker, O. , Ngo, T. D. , Redd, A. D. , Reynolds, S. J. , Morrow, R. A. , Manucci, J. L. , Serwadda, D. , Gray, R. H. , Quinn, T. C. , Neal, J. D. , Tobian, A. A. R. , Laeyendecker, O. , Ngo, T. D. , Redd, A. D. , Reynolds, S. J. , Ashley Morrow, R. , Manucci, J. L. , Serwadda, D.	2011	Performance of the Euroline Western blot assay in the detection of herpes simplex virus type 2 antibody in Uganda, China and the USA	east africa and east asia	consistently high sensetivity of euroline in china and uganda but specificity a lot lower (and also lower than kalon and hereselect) in uganda. Euroline is commercially available and easy to perform	International Journal of STD & AIDS
Ranu, H. , Lee, J. , Chio, M. , Sen, P.	2011	Tumour like presentations of anogenital herpes simplex in HIV positive patients	east asia	HSV 2 should be considered in nodular angogenital erosions in HIV positive people , PCR should be carried out as culture may be negative. Highlight urgent need for intervwntions to targt this syndemic relationship	International Journal of STD & AIDS
CanchéPech, J. R. , González Losa, M. del R. , Ayora Talavera, G. , Carrillo Martinez, J. , Sáenz Carbonell, L. A. , Córdova Lara, I. , Conde Ferráez, L.	2012	Herpes simplex virus infection in obstetric patients: comparing serology vs. real time PCR	South America	IgM did not correlate to genital tract shedding. So presence genital viral DNA (PCR) is better for identifying mothers at risk	
Tada, D. G. , Neeta, Khandelwal	2012	Serum HSV 1 and 2 IgM in patients of sexually transmitted diseases	South asia	hsv 2 igm could be used as a supportive diagnostic test for genital herpes and for survellance activities however, overall not hat helpful in knowing when the infection was acquired	Annals of Tropical Medicine and Public Health

Kapil, Goyal , Ratho, R. K. , Kanwar, A. J. , Baijayantimala, Mishra , Singh, M. P.	2013	Role of polymerase chain reaction in detection of genital herpes	south asia	PCR performed much better than tzanck smears, antigen detection and culture. 7 additional cases detected with PCR than with all other direct methods. Authors suggest that larger prospective studies should be carried outin order o replace viral culture with PCR as gold standard. advantages ove rculture in genital herpes: can detect subclinical shedding and undoagnosed symptomatic ulcers	Indian Journal of Dermatology, Venereology and Leprology
Rao, G. , Das, A. , Prabhakar, P. , Nema, V. , Risbud, A. R.	2013	Alteration in sample preparation to increase the yield of multiplex Polymerase Chain Reaction assay for diagnosis of genital ulcer disease	south asia	phenol chloroform extraction and ethanol precipitation helped eliminate PCR inhibitors and increase detected cases, but it is a long process and therefore not suitable for routine work, maybe for negative samples only	Indian Journal of Medical Microbiology
Rodrigues, D. , de Paris, F. , Paiva, R. M.	2013	Minimum detection limit of an in house nested PCR assay for herpes simplex virus and varicella zoster virus	South America	similar detection limit to previous studies	Revista da Sociedade Brasileira de Medicina Tropical
Tankhiwale, S. S. , Chavan, S. P.	2013	Comparative study of syndromic and etiological diagnosis of sexually transmitted infection	South Asia	confirmed that syndromic ha disadvantages and highlights the need to	International Journal of

		except human immunodeficiency virus in sexually transmitted infection and reproductive tract infection clinic attendees in central India		review its relevance regularly depending on geographical area and population and STI trends in that area	Medicine and Public Health
Amirjannati, N. , Yaghmaei, F. , Akhondi, M. M. , Nasiri, M. , Heidari Vala, H. , Sehhat, Z.	2014	Molecular and serologic diagnostic approaches; the prevalence of herpes simplex in idiopathic men infertile	south asia	PCR is prefered over serology	Iranian Journal of Reproductive Medicine
Banihashemi, M. , Pezeshkpoor, F. , Yousefzadeh, H. , Livani, F. , Ghandeharian, G.	2014	Tzanck smears in herpes simplex virus infections	South Asia	tzanck not very specific ad has limitations but is very cost effective therefore may be used when other methods are unavailable in resource poor settings	Iranian Journal of Dermatology
Gimenes, F. , Medina, F. S. , Abreu, A. L. P. de , Irie, M. M. T. , Esquiçati, I. B. , Malagutti, N. , Vasconcellos, V. R. B. , Discacciati, M. G. , Bonini, M. G. , Maria Engler, S. S. , Consolaro, M. E. L.	2014	Sensitive simultaneous detection of seven sexually transmitted agents in semen by multiplex PCR and of HPV by single PCR	South America	M PCR is an accurate and useful tool for detecting HSV 2 nd other STDs in sperm	PLoS ONE
Hong, YunJi , Lim, MiSuk , Hwang, SangMee , Kim, TaekSoo , Park, KyoungUn , Song, JungHan , Kim, EuiChong	2014	Detection of herpes simplex and varicella zoster virus in clinical specimens by multiplex real time PCR and melting curve analysis	east asia	accurate and suitable method for detecting and typing HSV infection, could replace culture	BioMed Research International
Otieno, F. O. , Ndivo, R. , Oswago, S. , Ondiek, J. , Pals, S. , McLellan Lemal, E. , Chen, R. T. , Chege, W. , Gray, K. M.	2014	Evaluation of syndromic management of sexually transmitted infections within the Kisumu Incidence Cohort Study	east africa	syndromic diagnosis of sti does not perform sufficiently well in Kismu, Kenya. Need more development of affordable cost effective laboratory methods	International Journal of STD & AIDS

Ryan, C. E. , Simbiken, C. S. , Agius, P. A. , Allen, J. , Sauk, J. , Kaima, P. , Kombati, Z. , Siba, P. , Kaldor, J. M. , Vallely, A.	2014	Comparative performance of the Kalon and HerpeSelect enzyme linked immunosorbant assays to determine the prevalence of herpes simplex virus type 2 in Papua New Guinea	east asia	herpeselect lacked specificity (many false positives likely) and terefore recommended kalon for future studies in PNG	Sexual Health
Shah, N. S. , Kim, E. , Ayala, F. de M. H. , Guardado Escobar, M. E. , Nieto, A. I. , Kim, A. A. , Paz Bailey, G.	2014	Performance and comparison of self reported STI symptoms among high risk populations MSM, sex workers, persons living with HIV/AIDS in El Salvador	central america	self reeported symptoms not a good metho for high risk people in el salvadoor, however due to poor lab resources and high prevalence of genital herpes perhaps overdiagnosis through synromic management would be acceptable, hoever, nt good for viral resistance. Need some sort of screening for high risk people in E.Sal	International Journal of STD & AIDS
Wu, DongSheng , Wu, YuanJian , Wang, LiuHong , Xu, WeiDong , Zhong, Qiao	2014	Evaluation of a novel array based toxoplasma, rubella, cytomegalovirus, and herpes simplex virus IgG enzyme linked immunosorbent assay and its comparison with virion/serion enzyme linked immunosorbent assays	east asia	BGI ELISA showed near perfect agreement with conventional ELISA and is a suitable mthod for clinical practice	Annals of Laboratory Medicine
Baetselier, I. de , Menten, J. , Cuylaerts, V. , Ahmed, K. , Deese, J. , Damme, L. van , Crucitti, T.	2015	Prevalence and incidence estimation of HSV 2 by two IgG ELISA methods among South African women at high risk of HIV	southern africa	kalon and hereselect similar performance (concordant). Kalon index reference djusted to 0.66 to catch early infections without losing specificity	PLoS ONE

Kalu, E. I. , Ojide, C. K. , Nwadike, V. U. , Ogbaini Emovon, E. , Okafor, G. O. C.	2015	Validity of genito urinary discharges, genital ulcers and genital rashes as indicators of seroincident HSV 2 infection	west africa	genito urinary discharges, genital ulcers and genital rashes are valis as a screening tool in porr resourced settings to suggest incident genital herpes	Asian Pacific Journal of Tropical Disease
Lamers, Susanna L. , Newman, Ruchi M. , Laeyendecker, Oliver , Tobian, Aaron A. R. , Colgrove, Robert C. , Ray, Stuart C. , Koelle, David M. , Cohen, Jeffrey , Knipe, David M. , Quinn, Thomas C.	2015	Global Diversity within and between Human Herpesvirus 1 and 2 Glycoproteins	west africa, southern africa and asia	glycoprotein g showed variation across all the different locations, this will help refine performance of diagnostic test. Hsv2 gG less diverse than 1	Journal of virology
Patel, E. U. , Manucci, J. , Kahle, E. M. , Lingappa, J. R. , Morrow, R. A. , Piwowar Manning, E. , James, A. , Maluzi, K. F. , Cheeba, M. M. , Gray, G. , Delany Moretlwe, S. , Inambao, M. , Vwalika, B. , Quinn, T. C. , Laeyendecker, O.	2015	Precision of the Kalon Herpes Simplex Virus type 2 IgG ELISA: an international inter laboratory assessment	Southern africa	high precision and reproducibility in different labs in southern african countries	BMC Infectious Diseases
Li, ZhiYan , Yan, Rong , Yan, CunLing , Liu, Ping , Feng, ZhenRu	2016	Evaluation of an automated chemiluminescent immunoassay in typing detection of IgG antibodies against herpes simplex virus	east asia	CLIA is plausible method of detecting hsv 2 gG	Journal of Clinical Laboratory Analysis
Nsobya, S. L. , Hewett, P. C. , Kalibala, S. , Mensch, B. S.	2016	Performance of Kalon herpes simplex virus 2 assay using dried blood spots among young women in Uganda	east africa	DBS with kalon had poor accuracy performance	African Journal of Laboratory Medicine
Vrushali, Patwardhan , Preena, Bhalla	2016	Role of type specific herpes simplex virus 1 and 2 serology as a diagnostic modality in patients	south asia	ELISA had better sensetivity and specificity in recurrent genital erpes	Indian Journal of Pathology & Microbiology

		with clinically suspected genital herpes: a comparative study in Indian population from a tertiary care hospital		episodes than in primary episodes. Could be suitable for use in supporting a diagnosis of recurrent genital ulcers	
Yang, JinFang , Zhao, ChangZhen , Lu, KeXin	2016	Development and application of a rapid detection system for human papillomavirus and Herpes simplex virus 2 by loop mediated isothermal amplification assay	east asia	accurate enough to be used as part of extensive screening of hsv 2 since it is ver cost efefctive	Microbial Pathogenesis
Hobbs, M. M. , Mwanyumba, S. W. , Luseno, W. K. , Hartman, S. , Halpern, C. T. , Hallfors, D. D. , Cho, HyunSan	2017	Evaluation of herpes simplex virus type 2 serological tests for use with dried blood spots in Kenya	east africa	kalon performance was poor, herpeselect has sen and specs of 98.8 and 98.9 . Suitable and valuable in low resource settings dur to convenience and utility of DBS compared to storing sera	Sexually Transmitted Diseases
Lima, L. R. P. , Silva, A. P. da , Schmidt Chanasit, J. , Paula, V. S. de	2017	Diagnosis of human herpes virus 1 and 2 (HHV 1 and HHV 2): use of a synthetic standard curve for absolute quantification by real time polymerase chain reaction	south america	" This study demonstrated that synthetic curves could be used as alternative standard curves for HHV diagnosis, since they showed similar re sults when compared to viral DNA curves."	Memórias do Instituto Oswaldo Cruz
Fettahlıoğlu Karaman, Bilge	2018	The role of Tzanck smear in the diagnosis of herpetic lesions older than three days	western asia (turkey)	tzanck smears still useful after 3 days especially if there are still vesicles	Cukurova University, 2018.
Finger Jardim, F. , Avila, E. C. , Hora, V. P. da , Santos, P. C. dos , Gonçalves, C. V. , Mor, G. , Martinez, A. M. B. de , Soares, M. A.	2018	Herpes simplex virus type 2 IgG antibodies in sera of umbilical cord as a proxy for placental infection in asymptomatic pregnant women	South America	circulating IgG can be indicative of placental infection in asymptomati women	American Journal of Reproductive Immunology

Kaida, A. , Dietrich, J. J. , Laher, F. , Beksinska, M. , Jaggernath, M. , Bardsley, M. , Smith, P. , Cotton, L. , Chitneni, P. , Closson, K. , Lewis, D. A. , Smit, J. A. , Ndung'u, T. , Brockman, M. , Gray, G.	2018	A high baurden of asymptomatic genital tract infections undermines the syndromic management approach among adolescents and young adults in South Africa: implications for HIV prevention efforts	Southern africa	syndromic diagnosis not sensetive enough in youths in SA and leaves many symptomatic patients at risk of HIV acquisition	BMC Infectious Diseases
Lee, ChunKiat , Chai, CheanNee , Capinpin, S. M. , Ang, A. , Ng, SauYoke , Lee, PeakLing , Ng, WaiSiong , Yan, G. , Lee, HongKai , Chiu, L. L. , Jureen, R. , Yan, B. , Loh, TzePing	2018	Evaluation of the Luminex ARIES HSV 1&2 assay and comparison with the FTD Neuro 9 and in house real time PCR assays for detecting herpes simplex viruses	east asia	ARIES is a valuable alternative for detecting HSV 2 in genital infection, particularly rapid method. Low mainenance and calibration requirements	Annals of Laboratory Medicine
Nasrallah, G. K., Dargham, S. R., Sahara, A. S. , Elsidiq, M. S. , Abu Raddad, L. J.	2019	Performance of four diagnostic assays for detecting herpes simplex virus type 2 antibodies in the Middle East and North Africa	north africa and western asia/middle east	good overall concordance ofall assays, however caution should be used using any of these alone in a epi study and a 2 test algorythm is suggested to improve sensetivity and specificity	Journal of Clinical Virology
Rodrigues, L. L. S. , Pilotto, J. H. , Lima, L. R. P. , Gaydos, C. A. , Hardick, J. , Morgado, M. G. , Martinelli, K. G. , Paula, V. S. de , Nicol, A. F.	2019	Self collected versus clinician collected samples for HSV 2 and HSV 2/HPV screening in HIV infected and uninfected women in the Tapajós region, Amazon, Brazil	South America	slef collection as good as clinician and could save time and resources	International Journal of STD & AIDS
Chakraborty, Payal , Norris, Alison H. , Huber Krum, Sarah , Garver, Sarah , Hood, Robert B. , Banda, Venson , Esber, Allahna , Patricia, Carr Reese , Krysiak, Robert , Turner, Abigail Norris	2020	An Assessment of Risk Factors for Herpes Simplex Virus Type 2 Infection in Malawian Women Using 2 Classifications for the HerpeSelect 2 Test	southeren africa (malawi)	diffferent risk facors associated with the diffrerent cutpoints need to be aware of which cutpount when recruiting 'high risk' people since the	Sexually Transmitted Diseases

				characteristics will be different	

HSV-2 Control measures

Authors	Year	Title	Geo_Area of study population	Main findings/Prevalence	Journal
Kamali, A f Quigley, M ; Nakiyingi, J ; Kinsman, J ; Kengeya-Kayondo, J ; Gopal, R ; Ojwiya, A ; Hughes, P ; Carpenter, L ; Whitworth, J.	2003	Syndromic management of sexually-transmitted infections and behaviour change interventions on transmission of HIV-1 in rural Uganda: a community randomised trial	East Africa	Incidence of HSV2 was lower in group A than in group C (incidence rate ratio 0·65, 0·53–0·80) however, more effective STI and behavioural interventions need to be developed for HIV control in mature epidemics	The Lancet
Hearst, Norman ; Chen, Sanny	2004	Condom Promotion for AIDS Prevention in the Developing World: Is It Working?	Multiple regions	Condoms are about 90 percent effective for preventing HIV transmission and HSV-2, and their use has grown rapidly in many countries. Condoms have produced substantial benefit in countries like Thailand, where both transmission and condom promotion are concentrated in the area of	Studies in Family Planning

Nahal B, *et al. BMJ Glob Health* 2024; 9:e012719. doi: 10.1136/bmjgh-2023-012719

				commercial sex. The public health benefit of condom promotion in settings with widespread heterosexual transmission, however, remains unestablished.In countries like Uganda that have curbed generalized epidemics, reducing the number of individuals' sex partners appears to have been more important than promoting the use of condoms	
Feldblum, Paul J ; Kuyoh, Maureen A ; Bwayo, Job J ; Omari, Mohamed ; Wong, Emelita L ; Tweedy, Kathryn G ;Welsh, Michael J.	2001	Female condom introduction and sexually transmitted infection prevalence: results of a community intervention trial in Kenya	East Africa	female condom introduction did not enhance STI prevention at the sesites. It is unclear which aspects of the intervention	Health
Van Damme, Lut ; Ramjee, Gita ; Alary, Michel ; Vuylsteke, Bea ; Chandeying, Verapol ; Rees, Helen ; Sirivongrangson, Pachara ; Tshibaka, Léonard Mukenge ; Ettiègne-Traoré, Virginie ; Uaheowitchai, Charn ; Karim, Salim S. Abdool ; Mâsse,	2002	Effectiveness of COL-1492, a nonoxynol-9 vaginal gel, on HIV-1 transmission in female sex workers: a randomised controlled trial	Multiple regions	No significant effect of nonoxynol-9 on HSV-2	The Lancet

Benoît ; Perriëns, Jos ; Laga, Marie					
Baeten, J. M ; Strick, L. B ; Lucchetti, A ; Whittington, W. L. H ; Sanchez, J ; Coombs, R. W ; Magaret, A ; Wald, A ; Corey, L ; Celum, C.	2008	Herpes simplex virus (HSV)-suppressive therapy decreases plasma and genital HIV-1 levels in HSV-2/HIV-1 coinfected women: a randomized, placebo-controlled, cross-over trial	South America	HIV-1 level was significantly lower during the valacyclovir arm, compared with the placebo arm (45% decrease [P<.001]), and cervical HIV-1 level (55% decrease [P<.001]),	Journal of Infectious Diseases
Fife KH, Warren TJ, Justus SE, Heitman CK	2008	An international, randomized, double-blind, placebo-controlled, study of valacyclovir for the suppression of herpes simplex virus type 2 genital herpes in newly diagnosed patients	Multiple regions		Sexually Transmitted Diseases
Nagot N, Ouédraogo A, Foulongne V, Konaté I, Weiss HA, Vergne L	2007	Reduction of HIV-1 RNA levels with therapy to suppress herpes simplex virus	West Africa		New England Journal of Medicine
Cowan, F. M ; Pascoe, S. J ; Barlow, K. L ; Langhaug, L. F ; Jaffar, S ; Hargrove, J. W ; Robinson, N. J ; Bassett, M. T	2008	A randomised placebo-controlled trial to explore the effect of suppressive therapy with acyclovir on genital shedding of HIV-1 and	Southren Africa	suppressive acyclovir therapy had no effect on the rate of HIV genital shedding despite a	Sexually Transmitted Infections

;Wilson, D ; Brown, D. W. G ; Hayes, R. J.		herpes simplex virus type 2 among Zimbabwean sex workers		reduction in genital HSV-2 of 24%	
Mayaud, P ; LeGoff, J ; Weiss, H. A ; Grésenguet, G ; Nzambi, K ; Bouhlal, H ; Frost, E ; Pépin, J ; Malkin, J. E ; Hayes, R. J ; Mabey, D. C. W ; Bélec, L.	2009	Impact of acyclovir on genital and plasma HIV-1 RNA, genital herpes simplex virus type 2 DNA, and ulcer healing among HIV-1-infected African women with herpes ulcers: a randomized placebo-controlled trial	Multiple (Ghana, Central African Republic)	cervicovaginal HSV-2 DNA (RR, 0.69; 95% CI, 0.4-1.3), had a lower quantity of HSV-2 DNA (-0.99 log(10) copies/mL; 95% CI, -1.8 to -0.2 log(10) copies/mL), and were more likely to have a healed ulcer (RR, 1.26; 95% CI, 0.9-1.9).	Journal of Infectious Diseases
Dunne, E. F ; Whitehead, S ;. Sternberg, M ; Thepamnuay, S ; Leelawiwat, W ; McNicholl, J. M ; Sumanapun, S. Tappero, J. W ; Siriprapasiri, T ; Markowitz, L.	2008	Suppressive acyclovir therapy reduces HIV cervicovaginal shedding in HIV- and HSV-2-infected women, Chiang Rai, Thailand	East Asia	Acyclovir reduced HIV genital shedding and plasma viral load among HIV-1- and herpes simplex virus type 2-coinfected women	JAIDS, Journal of Acquired Immune Deficiency Syndromes
Tobian, A. A. R ; Serwadda, D ; Quinn, T. C ; Kigozi, G ; Gravitt, P. E ; Laeyendecker, O ;Charvat, B ;Ssempijja, V ; Riedesel, M ; Oliver, A. E ;Nowak, R. G ;Moulton, L. H ; Chen, M. Z ; Reynolds, S. J ; Wawer, M. J. ; Gray, R. H.	2009	Male Circumcision for the Prevention of HSV-2 and HPV Infections and Syphilis	east Africa	At 24 months, the cumulative probability of HSV-2 seroconversion was 7.8% in the intervention group and 10.3% in the control group (adjusted hazard ratio in the intervention group, 0.72; 95% confidence interval [CI], 0.56 to 0.92; P=0.008)	New England Journal of Medicine

Sobngwi-Tambekou, J ; Taljaard, D ; Lissouba, P ; Zarca, K ; Puren, A ; Lagarde, E ; Auvert, B.	2009	Effect of HSV-2 serostatus on acquisition of HIV by young men: results of a longitudinal study in Orange Farm, South Africa	Southren Africa	the protective effect of male circumcision on HIV acquisition appears independent of HSV-2 serostatus.	Journal of Infectious Diseases
Martin, Emily T ; Krantz, Elizabeth ; Gottlieb, Sami L ; Magaret, Amalia S ; Langenberg, Andria ; Stanberry, Lawrence ; Kamb, Mary ;Wald, Anna	2009	A Pooled Analysis of the Effect of Condoms in Preventing HSV-2 Acquisition	Multiple regions	Consistent condom users (used 100% of the time) had a 30% lower risk of HSV-2 acquisition compared with those who never used condoms (hazard ratio [HR], 0.70; 95% confidence interval [CI], 0.40-0.94) (P = .01). Risk for HSV-2 acquisition increased steadily and significantly with each unprotected sex act (HR, 1.16; 95% CI, 1.08-1.25) (P < .001). Condom effectiveness did not vary by gender.	Archives of Internal Medicine
Gottlieb SL, Giersing BK, Hickling J, Jones R, Deal C, Kaslow DC	2019	Meeting report: Initial World Health Organization consultation on herpes simplex virus (HSV) vaccine preferred product characteristics	Multiple regions		Vaccine

Nahal B, et al. BMJ Glob Health 2024; 9:e012719. doi: 10.1136/bmjgh-2023-012719

Connie, ; Noah, ; Myron,	2005	Potential Effect of HIV Type 1 Antiretroviral and Herpes Simplex Virus Type 2 Antiviral Therapy on Transmission and Acquisition of HIV Type 1 Infection	Multiple regions	trials of ART or HSV-2 antiviral therapy are successful, prevention benefits may be difficult to translate to com-munity benefits; in general, trials of ART and HSV-2 antiviral therapy must be viewed as proof-of-concept studies. Favourable results can lead to the development of agents more appropriate to prevention and to operational strategies required for optimal implementation.	Oxford University Press (OUP)
Gregson, Simon ; Adamson, Saina ; Papaya, Spiwe ; Mundondo, Jephias ; Nyamukapa, Constance A ; Mason, Peter R ; Garnett, Geoffrey P ; Chandiwana, Stephen K ; Foster, Geoff ; Anderson, Roy M.	2007	Impact and Process Evaluation of Integrated Community and Clinic-Based HIV-1 Control: A Cluster-Randomised Trial in Eastern Zimbabwe	Southren Africa	Integrated peer education, condom distribution, and syndromic STI management did not reduce population-level HIV-1 and HSV-2 incidence in a declining epidemic, despite reducing HIV-1incidence in the immediate male target group.	PLoS Medicine

Paz-Bailey, G ; Ramaswamy, M ; Hawkes, S. J ; Geretti, A. M.	2006	Herpes simplex virus type 2: epidemiology and management options in developing countries	Multiple regions	multifaceted interventions are required to improve recognition of genital herpes, to prevent its spread and also to prevent its potential to promote HIV transmission in developing countriesare required to improve recognition of genital herpes, to prevent its spread and also to prevent its potential to promote HIV transmission in developing countries	Sexually Transmitted Infections
Jewkes, R ; Nduna, M ; Levin, J ; Jama, N ; Dunkle, K ; Puren, A ; Duvvury, N.	2008	Impact of Stepping Stones on incidence of HIV and HSV-2 and sexual behaviour in rural South Africa: cluster randomised controlled trial	Southren Africa	the programme was associated with a reduction of about 33%i n the incidence of HSV-2 (0.67, 0.46 to 0.97; P=0.036	BMJ
Prichard, Mark N ; Kaiwar, Ravi ; Jackman, Winthrop T ; Quenelle, Debra C ; Collins, Deborah J ; Kern, Earl R ; Kemble, George M ; Spaete, Richard R.	2005	Evaluation of AD472, a live attenuated recombinant herpes simplex virus type 2 vaccine in guinea pigs	global	Immunization with AD472 at doses of 1 × 105 PFU generally precluded colonization of the ganglia or establishment of latency by the challenge virus.	Vaccine

Paz-Bailey, G ; Sternberg, M ; Puren, A. J ; Markowitz, L. E ; Ballard, R ; Delany, S ; Hawkes, S ; Nwanyanwu, O ; Ryan, C ; Lewis, D. A.	2009	improvement in healing and reduction in HIV shedding with episodic acyclovir therapy as part of syndromic management among men: a randomized, controlled trial	Southren Africa	Acyclovir improved ulcer healing--61% of those receiving acyclovir healed by day 7, compared with 42% of those receiving placebo (adjusted relative risk, 1.4 [95% confidence interval, 1.1-1.8]; P= .003)	Journal of Infectious Diseases
Baeten, J. M ; Reid, S. E ; Delany-Moretlwe, S ; Hughes, J. P ; Wang, R. S ; Wilcox, E ; Limbada, M ; Akpomiemie, G ; Corey, L ; Wald, A ;Celum, C.	2012	Clinical and virologic response to episodic acyclovir for genital ulcers among HIV-1 seronegative, herpes simplex virus type 2 seropositive African women: a randomized, placebo-controlled trial	Southren Africa	episodic acyclovir therapy resulted in modestly greater likelihood of lesion healing (HR=1.48, p=0.098; mean 5.1 vs. 6.0 days) and cessation of HSV shedding (HR=1.88, p=0.008; mean 3.0 vs. 5.0 days) compared with placebo, similar to results from high-income countries.	Sexually Transmitted Diseases

Nahal B, *et al. BMJ Glob Health* 2024; 9:e012719. doi: 10.1136/bmjgh-2023-012719

Celum, C ; Batten, J. M.	2012	Tenofovir-based pre-exposure prophylaxis for HIV prevention: evolving evidence	Global	Peri-coital use of 1% tenofovir gel in CAPRISA 004 reduced HIV-1acquisition by 39% and HSV-2 acquisition by 51%. Daily oral FTC/TDF demonstrated 44%reduction in HIV-1 acquisition among men who have sex with men (MSM) in iPrEx. Both studiesshowed higher efficacy among those with higher adherence. Efficacy of daily oral TDF and FTC/TDF was 66% and 73%, respectively, among HIV-1 uninfected partners in an HIV-1serodiscordant partnership in the Partners PrEP Study. Efficacy of daily oral FTC/TDF was 66%in young heterosexuals in Botswana in the TDF-2 trial. The FEM-PrEP and VOICE studies inAfrican women found no efficacy with oral FTC/TDF and TDF, respectively. Safety andtolerability were excellent and limited	Current Opinion in Infectious Diseases

				resistance was observed in seroconverter	

Watson-Jones D, Weiss HA, Rusizoka M, Changalucha J, Baisley K, Mugeye K	2008	Effect of herpes simplex suppression on incidence of HIV among women in Tanzania	East Africa	RCT	New England Journal of Medicine
Fuchs, J ; Celum, C ; Wang, J ; Hughes, J ; Sanchez, J ; Cowan, F ; Reid, S ; Delany-Moretlwe, S; Corey, L; Wald, A.	2010	Clinical and virologic efficacy of herpes simplex virus type 2 suppression by acyclovir in a multi-continent clinical trial	Global	Overall, acyclovir was associated with a 47% [RR 0.53 (95% CI 0.46, 0.62)] reduction in GUD	Journal of Infectious Diseases
Moreno, R ; Nababan, H. Y ; Ota, E ; Wariki, W. M. V ; Ezoe, S ; Gilmour, S ; Shibuya, K.	2014	Structural and community-level interventions for increasing condom use to prevent the transmission of HIV and other sexually transmitted infections	Developing countires	no clear evidence that the intervention work	Cochrane Database of Systematic Reviews

Roxby, A. C.<br>Drake, A. L.<br>Ongecha-Owuor, F.<br>Kiarie, J. N.<br>Richardson, B; Matemo, D. N ; Overbaugh, J ; Emery, S ; John-Stewart, G. C ; Wald, A ; Farquhar, C.	2012	Effects of valacyclovir on markers of disease progression in postpartum women co-infected with HIV-1 and herpes simplex virus-2	East Africa	Women randomized to valacyclovir suppressive therapy during pregnancy and postpartum had greater increases in CD4 counts and smaller increases in plasma HIV-1 RNA levels than women in the placebo arm. Valacyclovir suppression during pregnancy and breastfeeding may improve outcomes and delay antiretroviral therapy for HIV-1/HSV-2 co-infected women.	PLoS ONE
Reynolds, S. J ; Makumbi, F ; Newell, K ; Kiwanuka, N ; Ssebbowa, P ; Mondo, G ; Boaz, I ; Wawer, M. J ; Gray, R. H ; Serwadda, D ; Quinn, T. C.	2012	Effect of daily aciclovir on HIV disease progression in individuals in Rakai, Uganda, co-infected with HIV-1 and herpes simplex virus type 2: a randomised, double-blind placebo-controlled trial	east Africa	Aciclovir reduces the rate of disease progression, with the greatest effect in individuals with a high baseline viral load. Howevver, no significant difference in HIV disease progression existed between participants in the treatment group and those in the placebo group who had baseline HIV viral loads of less than 50,000	Lancet Infectious Diseases

Phiri, S ; Hoffman, I. F ; Weiss, H. A ; Martinson, F ; Nyirenda, N ; Kamwendo, D ; Fiscus, S. A ; Chen, C. Y ; Miller, W. C ; Hoeven, L. van de ; Chilongozi, D ; Cohen, M. S ; Mayaud, P.	2010	Impact of aciclovir on ulcer healing, lesional, genital and plasma HIV-1 RNA among patients with genital ulcer disease in Malawi	Southren Africa	85% of ulcers were healed at day 14 with no difference between treatment arms (risk ratio (RR)=1.02, 95% CI 0.93 to 1.11). Among 244 HIV-1/HSV-2 co-infected individuals, aciclovir reduced lesional HIV-1 RNA (adjusted RR=0.64, 95% CI 0.41 to 0.99) and seminal HIV-1 RNA (adjusted RR=0.59, 95% CI 0.40 to 0.88), but not cervical HIV-1 RNA or plasma HIV-1 RNA.	Sexually Transmitted Infections
Obiero, J ; Mwethera, P. G ;Wiysonge, C. S.	2012	Topical microbicides for prevention of sexually transmitted infections	Multiple regions	Tenofovir reduced the incidence of herpes simplex virus type 2 (HSV-2) infection (one trial, 426 women; RR 0.55; 95% CI 0.37 to 0.83)	Cochrane Database of Systematic Reviews
Lou, Yijun ; Qesmi, Redouane ; Wang, Qian ; Steben, Marc ; Wu, Jianhong ;Heffernan, Jane M.	2012	Epidemiological Impact of a Genital Herpes Type 2<br>Vaccine for Young Females	Global	vaccination of some females reduces infection in men, which then reduces infection in women.	PloS one

Lingappa, Jairam R ; Baeten, Jared M ; Wald, Anna ; Hughes, James P ;Thomas, Katherine K ;. Mujugira, Andrew; Mugo, Nelly ; Bukusi, Elizabeth A.	2010	Daily aciclovir for HIV-1 disease progression in people dually infected with HIV-1 and herpes simplex virus type 2: a randomised placebo-controlled trial	Multiple (East Africa, Southren Africa)	Acyclovir reduced risk of HIV-1 disease progression by 16%; 284 participants assigned acyclovir versus 324 assigned placebo reached the primary endpoint (hazard ratio [HR] 0.84, 95% CI 0.71-0.98, p=0.03).	Elsevier BV
Tanton, Clare ; Weiss, Helen A ; Rusizoka, Mary ; Legoff, Jerome ; Changalucha, John ; Baisley, Kathy ; Mugeye, Kokugonza ; Everett, Dean ; Belec, Laurent ; Clayton, Tim C. ; Ross, David A. ; Hayes, Richard J. ; Watson-Jones, Deborah	2010	Long-term impact of acyclovir suppressive therapy on genital and plasma HIV RNA in Tanzanian women: a randomized controlled trial	East Africa	there was little difference between acyclovir and placebo arms for cervico-vaginal HIV-1 RNA detection, Acyclovir 400mg bid is unlikely to be a useful long-term intervention to reduce HIV transmission.	The Journal of infectious diseases
Cowan, Frances M ; Pascoe, Sophie Js ; Langhaug, Lisa F ; Mavhu, Webster ; Chidiya, Samson ; Jaffar, Shabbar ; Mbizvo, Michael T ; Stephenson, Judith M ; Johnson, Anne M ; Power, Robert M ; Woelk, Godfrey ; Hayes, Richard J.	2010	The Regai Dzive Shiri project: results of a randomized trial of an HIV prevention intervention for youth	Southren Africa	no impact of the intervention on prevalence of HSV-2	AIDS

Skoberne, M ; Cardin, R ; Lee, A ; Kazimirova, A ; Zielinski, V ; Garvie, D ; Lundberg, A ; Larson, S ; Bravo, F. J ; Bernstein, D. I ; Flechtner, J. B ; Long, D.	2013	An Adjuvanted Herpes Simplex Virus 2 Subunit Vaccine Elicits a T Cell Response in Mice and Is an Effective Therapeutic Vaccine in Guinea Pigs	Multiple regions	the frequency of recurrent viral shedding wasconsiderably reduced in GEN-003/MM-2-vaccinated animals but not in GEN-003- or MM-2-vaccinated animals	Journal of Virology
Morello, Christopher S ; Kraynyak, Kimberly A ; Levinson, Michael S ; hen, Zhijiang ; Lee, Kuo-Fen ; Spector, Deborah H.	2012	Inactivated HSV-2 in MPL/alum adjuvant provides nearly complete protection against genital infection and shedding following long term challenge and rechallenge	Multiple regions	optimized vaccine for-mulation of FI-HSV2 MPL/Alhydrogel given i.m. completely protected against detectable vaginal HSV-2 shedding in the majority of animal sand HSV-2 latent DNA in the DRG of all animals.	Elsevier BV
Free, C.; Roberts, I. G ; Abramsky, T ; Fitzgerald, M ; Wensley, F.	2011	A systematic review of randomised controlled trials of interventions promoting effective condom use	Multiple regions	The results are generally consistent withmodest benefits but there is considerable potential forbias due to poor trial quality. Because of the lowproportion of trials using the same outcome thepotential for bias from selective reporting of outcomesis considerable. Despite the public health importanceof increasing condom use there is little reliableevidence on the	Journal of Epidemiology & Community Health

				effectiveness of condom promotioninterventions	
Qesmi, Redouane ; Wang, Qian ; Steben, Marc ; Wu, Jianhong ; Heffernan, Jane M.	2012	Epidemiological Impact of a Genital Herpes Type 2 Vaccine for Young Females	developing countires	Computer simulation shows that an adolescent-only vaccination program may be effective in eliminating HSV-2 disease, however, the success of extinction greatly depends on the level of vaccine uptake, the vaccine efficacy, the age of sexual maturity and safe sex practices. However, the time course of eradication would take many years	PloS one

Nahal B, et al. BMJ Glob Health 2024; 9:e012719. doi: 10.1136/bmjgh-2023-012719

Dropulic, Lesia K ; Cohen, Jeffrey I.	2012	The challenge of developing a herpes simplex virus 2 vaccine	developing countires	HSV-2 vaccines that reduce primary disease and shedding are an important goal for an HSV-2 vaccine. While sterilizing immunity would be ideal, the observation that persons can become infected with more than one strain of HSV-2 suggests that this might not be achievable.	Expert review of vaccines
Halford, William P ; Püschel, Ringo ; Gershburg, Edward ; Wilber, Andrew ; Gershburg, Svetlana ; Rakowski, Brandon	2011	A Live-Attenuated HSV-2 ICP0– Virus Elicits 10 to 100 Times Greater Protection against Genital Herpes than a Glycoprotein D Subunit Vaccine	Multiple regions	In vivoimaging demonstrated that a luciferase-expressing HSV-2 challenge virus failed toestablish a detectable infection in 0DNLS-immunized mice, whereas the same virus readily infected naı¨ve and gD-2-immunized mice. Collectively, these results suggest that a HSV-2 vaccine might be more likely to prevent genital herpes if itcontained a live-attenuated HSV-2 virus rather than a single HSV-2 protein.	Plos one

Mujugira, Andrew ; Magaret, Amalia S ; Celum, Connie ; Baeten, Jared M ; Lingappa, Jairam R ; Morrow, Rhoda Ashley ; Fife, Kenneth H ; Delany-Moretlwe, Sinead ; de Bruyn, Guy ; Bukusi, Elizabeth A. ; Karita, Etienne ; Kapiga, Saidi ; Corey, Lawrence ; Wald, Anna; Partners in Prevention, H. S. V. H. I. V. Transmission Study Team	2013	Daily acyclovir to decrease herpes simplex virus type 2 (HSV-2) transmission from HSV-2/HIV-1 coinfected persons: a randomized controlled trial	East Africa, Southren Africa	Treatment of African HSV-2/HIV-1–infected persons with daily suppressive acyclovir did not decrease risk of HSV-2 transmission to susceptible partners	The Journal of infectious diseases
Doyle, Aoife M ; Ross, David A ; Maganja, Kaballa ; Baisley, Kathy ; Masesa, Clemen ;Andreasen, Aura ; Plummer, Mary L ; Obasi, Angela I. N ; Weiss, Helen A ; Kapiga, Said ;Watson-Jones, Debora ; Changalucha, John ; Hayes, Richard J.	2010	Long-Term Biological and Behavioural Impact of an Adolescent Sexual Health Intervention in Tanzania: Follow-up Survey of the Community-Based MEMA kwa Vijana Trial	East Africa	Prevalences ofthe primary outcomes HIV and HSV-2 were 1.8% and 25.9% in males and 4.0% and 41.4% in females, respectively. Theintervention did not significantly reduce risk of HIV (males adjusted prevalence ratio [aPR] 0.91, 95%CI 0.50–1.65; femalesaPR 1.07, 95%CI 0.68–1.67) or HSV-2 (males aPR 0.94, 95%CI 0.77–1.15; females aPR 0.96, 95%CI 0.87–1.06	PLoS Medicine

Soares AR, Robaina MCS, Mendes GS, Silva TSL, Gestinari LMS, Pamplona OS	2012	Antiviral activity of extracts from Brazilian seaweeds against herpes simplex virus	Brazil		Revista Brasileira de Farmacognosia
Alsallaq RA, Schiffer JT, Longini Jr IM, Wald A, Corey L, Abu-Raddad LJ	2010	Population level impact of an imperfect prophylactic HSV-2 vaccine	Developing countries	population and individual effects of HSV-2 vaccine	Sexually transmitted diseases
Johnston, Christine ; Koelle, David M ; Wald, Anna	2014	Current status and prospects for development of an HSV vaccine	global	the importance of the mucosal immune response, availability of novel adjuvants and delivery mechanisms, identification of T cell epitopes via proteomic screening and advancement in replication competent and replication-incompetent candidates. In	Vaccine

				addition, we have learned from past vaccine studies; we need to depend on objective evidence of seroconversion rather than the variable phenotype of clinical disease in preventative vaccine studies. Similarly, viral shedding should be measured rather than recurrence rate as the most sensitive tool to evaluate both prophylactic and therapeutic vaccines, to provide data on potential impact of vaccination on HSV transmission	
Moussa HS, Ibrahem ABB, El-Sayed AFM, Mohammed FA	2015	In vitro evaluation of anti-microbial activities of marine Streptomyces against viral models, bacterial and fungal strains.	In vitro study aimed at developing countries		International Journal of Virology

Roberts, Lindi; Liebenberg, Lenine; Barnabas, Shaun ; Passmore, Jo-Ann	2012	Vaginal microbicides to prevent human immunodeficiency virus infection in women: Perspectives on the female genital tract, sexual maturity and mucosal inflammation	Multiple regions	vaginal microbicides offer an important female-controlled strategy to prevent sexual transmission ofHIV. Use of antiretroviral drugs such as TFV 1% in coitally linked topical microbicide formulations hasrecently proven to be a moderately effective tool at preventing new infections in women 18 years and older	Best Practice & Research Clinical Obstetrics & Gynaecology
García, Patricia J ; Holmes, King K ; Cárcamo, César P ; Garnett, Geoff P ; Hughes, James P ; Campos, Pablo E ; Whittington, William L. H ; Peru, Preven Study Team	2012	Prevention of sexually transmitted infections in urban communities (Peru PREVEN): a multicomponent community-randomised controlled tria	South America	We report a non-significant reduction in prevalence of STIs in young adults, adjusted for baseline prevalence, in intervention cities compared with control cities (relative risk 0·84, 95% CI 0·69–1·02; p=0·096)	Lancet (London, England)
Hallfors, D. D ; Cho, H. S ; Rusakaniko, S ; Mapfumo, J ; Iritani, B ; Zhang, L ; Luseno, W ; Miller, T.	2015	The impact of school subsidies on HIV-related outcomes among adolescent female orphans	Southren Africa	The two groups did not differ on HIV or HSV-2 biomarkers	Journal of Adolescent Health

Low, A. J ; Nagot, N ; Weiss, H. A ; Konate, I ; Kania, D ; Segondy, M ; Meda, N ; Perre, P. van de ; Mayauda, P.	2016	Herpes Simplex Virus Type-2 Cervicovaginal Shedding Among Women Living With HIV-1 and Receiving Antiretroviral Therapy in Burkina Faso: An 8-Year Longitudinal Study	West Africa	Cervicovaginal HSV-2 DNA was detected in 42% of women (99 of 236) in 8.2% of visits . ART was associated with a reduction in the odds of HSV-2 shedding, which declined for each year of ART use (odds ratio [OR], 0.74; 95% confidence interval [CI], .59–.92).	Journal of Infectious Diseases
Yuan, Tanwei ; Fitzpatrick, Thomas ; Ko, Nai-Ying ; Cai, Yong ; Chen, Yingqing ; Zhao, Jin ; Li, Linghua ; Xu, Junjie ; Gu, Jing ; Li, Jinghua ; Hao, Chun ; Yang, Zhengrong ; Cai, Weiping ; Cheng, Chien-Yu ;Luo,;Zhenzhou<br>Zhang, Kechun<br>Wu, Guohui<br>Meng, Xiaojun<br>Grulich, Andrew E.<br>Hao, Yuantao<br>Zou, Huachun<br>Majiwa, M.<br>Onyango, C. O.<br>Kuile, F. O. ter<br>Phillips-Howard, P. A.	2019	Circumcision to prevent HIV and other sexually transmitted infections in men who have sex with men: a systematic review and meta-analysis of global data	Developing countires	Circumcision was associated with reduced odds of herpes simplex virus (HSV) infection among MSM overall (0·84, 0·75–0·95; k=5; I^2=0%)	The Lancet. Global health

Nahal B, *et al. BMJ Glob Health* 2024; 9:e012719. doi: 10.1136/bmjgh-2023-012719

Stoner MCD, Neilands TB, Kahn K, Hughes JP, Gómez-Olivé FX, Twine R	2019	Multilevel measures of education and pathways to incident Herpes simplex virus type 2 in adolescent girls and young women in South Africa	Southern Africa		Journal of Adolescent Health
Farr Zuend, Christina , Nomellini, John F , Smit, John, Horwitz, Marc S , Townsend, J.	2018	Generation of a Dual-Target, Safe, Inexpensive Microbicide that Protects Against HIV-1 and HSV-2 Disease	Southren Africa	Protection from HSV-2 disease was strongly associated with early cytokine production in the vaginal tract. Our data support the potential for a dual-target microbicide that can protect against both HIV-1 and HSV-2, which could have an enormous impact on public health	Scientific Reports
Johnston, Christine ; Gottlieb, Sami L ; Wald, Anna	2016	Status of vaccine research and development of vaccines for herpes simplex virus	Multiple regions	provide an overview of efforts to develop HSV-2 vaccines, including a discussion of the clinical need for an HSV vaccine, and status of research and development with an emphasis on recent insights from trials of vaccine candidates in clinical testing. In addition, we will touch upon aspects of HSV vaccine development relevant to	Vaccine

				low and middle income countries	
Jiang, Yu-Chen ; Feng, Hui ; Lin, Yu-Chun ; Guo, Xiu-Rong	2016	New strategies against drug resistance to herpes simplex virus	Multiple regions	new antiviral drugs with different antiviral actions, includ- ing new antiviral targets, new antiviral mechanisms, and new antiviral molecules, are required. Janus-type nucleosides have two different faces (mimicking the natural purine and pyrimidine systems) in one molecule, and these drugs may pair with diverse natural bases via rotation around the glycosyl bond, which further induces viral lethal mutation. Therefore, unique Janus-type nucleoside analogues possess great potential in the exploitation of new lethal mutagenesis drugs as	International journal of oral science

				novel strategies for antiviral chemotherapy.	
Shahzad, Naveed ; Farooq, Roman ; Aslam, Bilal ; Umer, Muhammad	2017	Microbicides for the Prevention of HPV, HIV-1, and HSV-2: Sexually Transmitted Viral Infections	South Asia	More trials can guide for more development of more rationally, accurately, behaviorally, and socially accepted and single solution maximum potential STIs.	Fundamentals of Sexually Transmitted Infections (Book)

Egan, Kevin P ; Hook, Lauren M ; Naughton, Alexis ; Pardi, Norbert ; Awasthi, Sita ; Cohen, Gary H ; Weissman, Drew ; Friedman, Harvey M.	2020	An HSV-2 nucleoside-modified mRNA genital herpes vaccine containing glycoproteins gC, gD, and gE protects mice against HSV-1 genital lesions and latent infection	Global	In the two experiments, them RNA vaccine prevented death and genital disease in 54/54(100%) mice infected with HSV-1 and 20/20 (100%) with HSV-2. They prevented HSV DNA from reaching the doral root ganglia,the site of virusl atency, in 29/30(97%) mice infected with HSV-1 and 10/10 (100%) with HSV-2.	PLOS Pathogens
Magaret, Amalia S ; Mujugira, Andrew ; Hughes, James P ; Lingappa, Jairam ; Bukusi, Elizabeth A ; DeBruyn, Guy ;Delany-Moretlwe, Sinead ; Fife, Kenneth H ; Gray, Glenda E ; Kapiga, Saidi ; Karita, Etienne ; Mugo, Nelly R ; Rees, Helen ; Ronald, Allan ; Vwalika, Bellington ; Were, Edwin ; Celum, Connie ; Wald, Anna ; Partners in Prevention, H. S. V. H. I. V. Transmission Study Team	2016	Effect of Condom Use on Per-act HSV-2 Transmission Risk in HIV-1, HSV-2-discordant Couples	Multiple regions	Transmission of HSV-2 occurred in 68 couples, including 17 with susceptible women and 51 with susceptible men. The highest rate of transmission was from men to women: 28.5 transmissions per 1000 unprotected sex acts. We found that condoms were differentially protective against HSV-2 transmission by sex; condom use reduced per-act risk of transmission from men to women by 96% (P < .001) and marginally from	Clinical infectious diseases : an official publication of the Infectious Diseases Society of America

				women to men by 65% (P = .060).	
Ceña-Diez, Rafael; Vacas-Córdoba, Enrique ; García-Broncano, Pilar ; de la Mata, F. J. ; Gómez, Rafael ; Maly, Marek ; Muñoz-Fernández, M. Ángeles	2016	Prevention of vaginal and rectal herpes simplex virus type 2 transmission in mice: mechanism of antiviral action	Multiple regions	Topical vaginal or rectal administration of G1-S4 or G2-S16 prevents HSV-2 transmission in BALB/c mice in values close to 100%	International journal of nanomedicine

HSV-2 and HIV interactions

Authors	Year	Title	Geographical area of study population	Study design	sample size	Journal	Study topic	Main findings

Mbopi-Kéou, F. X.,Grésenguet, G.,Mayaud, P.,Weiss, H. A.,Gopal, R.,Matta, M.,Paul, J. L.,Brown, D. W. G.,Hayes, R. J.,Mabey, D. C. W.,Bélec, L.	2000	Interactions between herpes simplex virus type 2 and human immunodeficiency virus type 1 infection in African women: opportunities for intervention	central Africa (CAR)	cross sectional study	300	Journal of Infectious Diseases	biological relationship: comparing serology, genital DNA and shedding in HIV infected and non infected	HSV shedding, antibody presence and HSV DNA presence were all more prevalent in HIV positive persons. Positive significant correlation between HIV RNA and HSV-2 DNA levels in genitals in dually infected women. They suggested more RCT research with HIV shedding or incidence as outcomes
Weiss, H. A.,Buvé, A.,Robinson, N. J.,Dyck, E. van,Kahindo, M.,Anagonou, S.,Musonda, R.,Zekeng, L.,Morison, L.,Caraël, M.,Laga, M.,Hayes, R. J.	2001	The epidemiology of HSV-2 infection and its association with HIV infection in four urban African populations	West, East and Southern Africa	cross sectional study	2000	AIDS	epidemiologic al/prevalence relationship	strong association between HSV-2 and Hiv prevalence (aOR between 4 and 8 in different groups)

Auvert, B.,Ballard, R.,Campbell, C.,Caraël, M.,Carton, M.,Fehler, G.,Gouws, E.,MacPhail, C.,Taljaard, D.,Dam, J. van,Williams, B.	2001	HIV infection among youth in a South African mining town is associated with herpes simplex virus-2 seropositivity and sexual behaviour	Southern Africa	cross sectional study	1507	AIDS	epidemiological/prevalence relationship	HSV-2 a strong independent RF for HIV (OR 5.3 for men and 8.4 for women). Hsv plays major role in spread of HIV. Suggest prompt treatment of STIs and encourage condom use and school based sexual education
Gray RH, Wawer MJ, Brookmeyer R, Sewankambo NK, Serwadda D, Wabwire-Mangen F	2001	Probability of HIV-1 transmission per coital act in monogamous, heterosexual, HIV-1-discordant couples in Rakai, Uganda	East Africa			The Lancet		

McClelland, R. S.,Wang, C. C.,Overbaugh, J.,Richardson, B. A.,Corey, L.,Ashley, R. L.,Mandaliya, K.,Ndinya-Achola, J.,Bwayo, J. J.,Kreiss, J. K.	2002	Association between cervical shedding of herpes simplex virus and HIV-1	East Africa	cross sectional study	200	AIDS	Biological: relationship between cervical shedding	significant association between HIV RNA and HSV DNA cervical shedding (r=0.24 Pearson). Researchers suggest more prospective studies to investigate relationship between non ulcerative HSV reactivation and hIV shedding
Mbizvo, E. M.,Sia, E. M.,Stray-Pedersen, B.,Chirenje, M. Z.,Munjoma, M.,Hussain, A.	2002	Association of herpes simplex virus type 2 with the human immunodeficiency virus among urban women in Zimbabwe	Southern Africa	cross sectional study	393	International Journal of STD & AIDS	epidemiological/prevalence relationship	HSV-2 positive women were twice as likely to be HIV positive (OR 2.05). Hsv-1 thought to contribute more to HIV than syphilis. Authors communicate urgent need for HSV 2 vaccine

Rodríguez, M. del M. P.,Obasi, A.,Mosha, F.,Todd, J.,Brown, D.,Changalucha, J.,Mabey, D.,Ross, D.,Grosskurth, H.,Hayes, R.	2002	Herpes simplex virus type 2 infection increases HIV incidence: a prospective study in rural Tanzania	East Africa	unmatched case control study nested within a prospective RCT	763	AIDS	epidemiological: prevalent HSV and incident HIV	significant association between HSV and HIV incidence. Stronger in men (OR 6.2) than women(OR 1.32). Stronger association in those who seroconverted during study (new HSV infections). Population attributable fraction of incident HIV due to HSV was 74% in en and 22% in women. HSv plays big role in HIV transmission in Tanzania and urgent need for HSV control measures to control HIV in Africa.

Ahmed, H. J.,Mbwana, J.,Gunnarsson, E.,Ahlman, K.,Guerino, C.,Svensson, L. A.,Mhalu, F.,Lagergard, T.	2003	Etiology of genital ulcer disease and association with human immunodeficiency virus infection in two Tanzanian cities	East Africa	cross sectional study	195	Sexually Transmitted Diseases	epidemiological/prevalence relationship	HIV prevalence significant higher in HSV positive people than negative in all areas. Women with HSV more likely to be HIV positive than men with HSV
Mbopi-Kéou, F. X.,Legoff, J.,Grésenguet, G.,Ali, Si-Mohamed,Matta, M.,Mayaud, P.,Andreoletti, L.,Malkin, J. E.,Weiss, H.,Brown, D.,Bélec, L.	2003	Genital shedding of herpes simplex virus-2 DNA and HIV-1 RNA and proviral DNA in HIV-1- and herpes simplex virus-2-coinfected African women	central Africa (CAR)	cross sectional study	300	JAIDS, Journal of Acquired Immune Deficiency Syndromes	biological: HIV pro virla DNA genital load in dually infected persons (effect of HSV on genital shedding)/transmission	those infected with serum HSV and HIV were twice as likely to be shedding HSV than those HSV positive and HIV negative. No association between genital proviral HIV DNA and genital HSV DNA levels, or between genital HIV RNA and genital HSV DNA shedding. There was a significant correlation between genital HIV RNA and genital HSV dNA amongst dually infected women, but only in HSV shedders.

Serwadda, D.,Gray, R. H.,Sewankambo, N. K.,Wabwire-Mangen, F.,Chen, M. Z.,Quinn, T. C.,Lutalo, T.,Kiwanuka, N.,Kigozi, G.,Nalugoda, F.,Meehan, M. P.,Morrow, R. A.,Wawer, M. J.	2003	Human immunodeficiency virus acquisition associated with genital ulcer disease and herpes simplex virus type 2 infection: a nested case-control study in Rakai, Uganda	East Africa	case control study	248	Journal of Infectious Diseases	biological: timing of HSV relative to HIV seroconversion	those who seroconverted (HIV) were more likely to be HSV seropositive (OR 1.7), HIV viral load was higher in dually infected
Reynolds, S. J.,Risbud, A. R.,Shepherd, M. E.,Zenilman, J. M.,Brookmeyer, R. S.,Paranjape, R. S.,Divekar, A. D.,Gangakhedkar, R. R.,Ghate, M. V.,Bollinger, R. C.,Mehendale, S. M.	2003	Recent herpes simplex virus type 2 infection and the risk of human immunodeficiency virus type 1 acquisition in India	South Asia	cohort study	2737	Journal of Infectious Diseases	effect of genital herpes (new and old infections) on HIV acquisition	HSV prevalent cases more likely to acquire HIV (hazard ratio 1.67). Stronger relationship between recent incident HSV (<6m) and HIV acquisition (hazard ratio 3.81). Suggest that HSV prevention will help reduce HIV acquisition risk

Baeten, J. M.,McClelland, R. S.,Corey, L.,Overbaugh, J.,Lavreys, L.,Richardson, B. A.,Wald, A.,Mandaliya, K.,Bwayo, J. J.,Kreiss, J. K.	2004	Vitamin A supplementation and genital shedding of herpes simplex virus among HIV-1-infected women: a randomized clinical trial	East Africa	RCT	376	Journal of Infectious Diseases	effect of vitamin A on HSV shedding in dually infected people	vitamin A unlikely to reduce vaginal HSV 2 shedding in dually infected, secondary finding was that those who were HSV shedding had higher HIV shedding
Duffus, W. A.,Mermin, J.,Bunnell, R.,Byers, R. H.,Odongo, G.,Ekwaru, P.,Downing, R.	2005	Chronic herpes simplex virus type-2 infection and HIV viral load	East Africa	cross sectional study	339	Internation al journal of STD & AIDS	natural history of HSV and HIV coinfection: effect of HSV on viral load and cd4	coinfection associated with higher HIV viral load. Chronic HSV may have a negative impact on clinical course of HIV
Nadal, S. R.,Calore, E. E.,Manzione, C. R.,Horta, S. C.,Ferreira, A. F.,Almeida, L. V.	2005	Hypertrophic herpes simplex simulating anal neoplAsia in AIDS patients: report of five cases	South America	case series	5	Diseases of the Colon and Rectum	effect of HSV on HIV clicical course/natura l hx: anal neoplAsia	HSV 2 may cause verrucous lesion that can lead to neolpAsia in AIDS pts on HAART

Gita, Ramjee,Williams, B.,Gouws, E.,Dyck, E. van,Deken, B. de,Karim, S. A.	2005	The impact of incident and prevalent herpes simplex virus-2 infection on the incidence of HIV-1 infection among commercial sex workers in South Africa	Southern Africa	cohort study	187	JAIDS, Journal of Acquired Immune Deficiency Syndromes	effect of incident and prevalent HSV on HIV acquisition	no sig difference in HIV incidence between people who were HSV positive or negative throughout trial. However, those with incident HSV were more likely to acquire HIV than those with prevalent HSV (hazard ratio 6). So, suggests that new HSV infection increases HIV acquisition risk and the risk decreases with time, this effect seems greater in men.

Nahal B, *et al. BMJ Glob Health* 2024; 9:e012719. doi: 10.1136/bmjgh-2023-012719

Chu, K.,Jiamton, S.,Pepin, J.,Cowan, F.,Mahakkanukrauh, B.,Suttent, R.,Robinson, N. J.,Deslandes, S.,Frost, E.,Chaisilwattana, P.,Suthipinittharm, P.,Grosskurth, H.,Brown, D.,Jaffar, S.	2006	Association between HSV-2 and HIV-1 viral load in semen, cervico-vaginal secretions and genital ulcers of Thai men and women	East Asia	cross sectional study	104	International Journal of STD & AIDS	effect of HSV on HIV viral load in different samples	presence of HSV shedding did not affect HIV viral load in semen and vagina. Findings suggest that HSV does not cause increased viral loads in genital secretions.
Cowan, F. F.,Pascoe, S. J. S.,Barlow, K. L.,Langhaug, L. F.,Jaffar, S.,Hargrove, J. W.,Robinson, N. J.,Latif, A. S.,Bassett, M. T.,Wilson, D.,Brown, D. W. G.,Hayes, R. J.	2006	Association of genital shedding of herpes simplex virus type 2 and HIV-1 among sex workers in rural Zimbabwe	Southern Africa	cross sectional study	214	AIDS	Shedding/transmission	almost all HIV positive were co infected (95%). No apparent relationship between HSV shedding and HIV 1 shedding

Lama, J. R.,Lucchetti, A.,Suarez, L.,Laguna-Torres, V. A.,Guanira, J. V.,Pun, M.,Montano, S. M.,Celum, C. L.,Carr, J. K.,Sanchez, J.,Bautista, C. T.,Sanchez, J. L.	2006	Association of herpes simplex virus type 2 infection and syphilis with human immunodeficiency virus infection among men who have sex with men in Peru	South America	cross sectional study	3280	Journal of Infectious Diseases	prevalence	80% of HIV seroositive were HSV2 seropositive. Hsv2 seropositivity a significant predictor of HIV seropositiviy. Hsv control measure should help reduce HIV infection in msmin Peru
Ouedraogo, A.,Nagot, N.,Vergne, L.,Konate, I.,Weiss, H. A.,Defer, M. C.,Foulongne, V.,Sanon, A.,Andonaba, J. B.,Segondy, M.,Mayaud, P.,Perre, P. D. van	2006	Impact of suppressive herpes therapy on genital HIV-1 RNA among women taking antiretroviral therapy: a randomized controlled trial	West Africa	RCT	60	AIDS	effect of HSV on genital HIV rna SHEDDING	overall no association between valaciclovir use and HIV shedding. But amongst the HIV sheddersonly , valacyclovir was associated with reduce genital HIV shedding. Conc: HSV-2 may cause replication of genital HIV in dually infected women on haart

Ao TTH, Sam NE, Masenga EJ, Seage GR, III, Kapiga SH	2006	Human immunodeficiency virus type 1 among bar and hotel workers in northern Tanzania: the role of alcohol, sexual behavior, and herpes simplex virus type 2	East Africa			Sexually Transmitte d Diseases.		

Legoff, Jérôme,Bouhlal, Hicham,Grésen guet, Gérard,Weiss, Helen,Khonde, Nzambi,Hocini, Hakim,Désiré, Nathalie,Si-Mohamed, Ali,de Dieu Longo, Jean,Chemin, Cécile,Frost, Eric,Pépin, Jacques,Malkin, Jean-Elie,Mayaud, Philippe,Bélec, Laurent	2006	Real-time PCR quantification of genital shedding of herpes simplex virus (HSV) and human immunodeficiency virus (HIV) in women coinfected with HSV and HIV	West and central Africa	cross sectional study	269	Journal of clinical microbiolo gy	accuracy of real time pcr in detecting HSV and HIV shedding in dually infected persons	real time PCR is a suitable method of measuring HIV and HSV shedding for future studies
Santos, F. C.,Oliveira, S. A. de,Setúbal, S.,Camacho, L. A. B.,Faillace, T.,Leite, J. P. G.,Velarde, L. G. C.	2006	Seroepidemiological study of herpes simplex virus type 2 in patients with the acquired immunodeficiency syndrome in the City of Niterói, Rio de Janeiro, Brazil	South America	cross sectional study	150	Memórias do Instituto Oswaldo Cruz	associations in prevalence	52% of participant were HSV seropositive. Only 20% of HSV positive were aware of their infection.

LeGoff, J.,Weiss, H. A.,Gresenguet, G.,Nzambi, K.,Frost, E.,Hayes, R. J.,Mabey, D. C. W.,Malkin, J. E.,Mayaud, P.,Belec, L.	2007	Cervicovaginal HIV-1 and herpes simplex virus type 2 shedding during genital ulcer disease episodes	West and central Africa -urban	cross sectional study	441	AIDS	correlates of HSV and HIV SHEDDING	genital herpes infection was associated with increased genital HIV shedding and plasma HIV viral load in co infected women with genital ulcer
Ziyaeyan, M.,Alborzi, A.,Japoni, A.,Kadivar, M.,Davarpanah, M. A.,Pourabbas, B.,Abassian, A.	2007	Frequency of acyclovir-resistant herpes simplex viruses isolated from the general immunocompetent population and patients with acquired immunodeficiency syndrome	South Asia	cross sectional study	133	International Journal of Dermatology	occurance of acyclovir resistance HSV strains in AIDS patients	AIDS patients are much more likely to be infected with acyclovir resistant strains of HSV2. alternative anti virals should be used in AIDS pts

Zuckerman, R. A.,Lucchetti, A.,Whittington, W. L. H.,Sánchez, J.,Coombs, R. W.,Zuñiga, R.,Magaret, A. S.,Wald, A.,Corey, L.,Celum, C.	2007	Herpes simplex virus (HSV) suppression with valacyclovir reduces rectal and blood plasma HIV-1 levels in HIV-1/HSV-2-seropositive men: a randomized, double-blind, placebo-controlled crossover trial	South America	cross over RCT	20	Journal of Infectious Diseases	effect of valcyclovir on genital and serum HIV levels	valacyclovir was associated with a significant reduction in rectal and plasma HIV levels in co-infected msm not on ART. Valacyclovir may be useful in affecting the clinical course of HIV in coinfected men not on ART and also may reduce transmission.
Drake, Alison L.,John-Stewart, Grace C.,Wald, Anna,Mbori-Ngacha, Dorothy A.,Bosire, Rose,Wamalwa, Dalton C.,Lohman-Payne, Barbara L.,Ashley-Morrow, Rhoda,Corey, Lawrence,Farquhar, Carey	2007	Herpes simplex virus type 2 and risk of intrapartum human immunodeficiency virus transmission	East Africa	nested case control study	175	Obstetrics and gynecology	transmission: effect of HSV2 on HIV transmission during birth	87% of the pregnant mothers with HIV were dually had HSV.

Baeten, J. M.,Benki, S.,Chohan, V.,Lavreys, L.,McClelland, R. S.,Mandaliya, K.,Ndinya-Achola, J. O.,Jaoko, W.,Overbaugh, J.	2007	Hormonal contraceptive use, herpes simplex virus infection, and risk of HIV-1 acquisition among Kenyan women	East Africa	cohort study	1206	AIDS	effect of genital herpes infection on the relationship between hormonal contraceptive use and HIV ACQUISITION	both hormonal contraceptives and HSV 2 infection were separately associated with acquisition, however HSV infection did not increase the HIV risk in hormone contraceptive users. They both increased the risk separately.
Brown, J. M.,Wald, A.,Hubbard, A.,Rungruengthanakit, K.,Chipato, T.,Rugpao, S.,Mmiro, F.,Celentano, D. D.,Salata, R. S.,Morrison, C. S.,Richardson, B. A.,Padian, N. S.	2007	Incident and prevalent herpes simplex virus type 2 infection increases risk of HIV acquisition among women in Uganda and Zimbabwe	East and Southern Africa	prospective cohort study	4531	AIDS	effect of incident and prevalent HSV on HIV acquisition	prevalent and incident HSV associated with higher risk of HIV acquisition in both countries. Incident HSV had more of an effect (hazarad ratio 4.6 to 8.6). Hsv had a greater effect in zimbabwe. POPULATION ATTRIBUTABLE RISK for HIV due to incidence and prevalent HSV was 42% in India and 65% in zimbabwe. HSV

								PLAYS BIG ROLE IN HIV ACQUISITION.

Rebbapragada, Anuradha,Wachihi, Charles,Pettengell, Christopher,Sunderji, Sherzana,Huibner, Sanja,Jaoko, Walter,Ball, Blake,Fowke, Keith,Mazzulli, Tony,Plummer, Francis A.,Kaul, Rupert	2007	Negative mucosal synergy between Herpes simplex type 2 and HIV in the female genital tract	East Africa	cross sectional study	102	AIDS (London, England)	mucosal interactions between HSV and HIV in the genital tract and effect on transmission	found 'negative mucosal synergy' where there is a vicious cycle. Genital HSV causes increased HIV target cells- HIV infection affects genital HSV immune regulation- HSV reactivation causes increased transmission of HSV and HIV.
Freeman, E. E.,Orroth, K. K.,White, R. G.,Glynn, J. R.,Bakker, R.,Boily, M. C.,Habbema, D.,Buvé, A.,Hayes, R.	2007	Proportion of new HIV infections attributable to herpes simplex 2 increases over time: simulations of the changing role of sexually transmitted infections in sub-Saharan African HIV epidemics	West, East and Southern Africa	cross sectional modelling study	999	Sexually Transmitted Infections	modelling effect of HSV on HIV incidence (Population attributable fraction)	HSV2 has an increasing effect on HIV incidence as a model epidemic progresses (PAF 0f 35-48% 15 years in to epidemic). Effect of HSV on HIV infectivity (transmission) thought to have more of an effect than effect of HSV on HIV acquisition. Hsv2 programmes may be

Nahal B, *et al. BMJ Glob Health* 2024; 9:e012719. doi: 10.1136/bmjgh-2023-012719

								essential in HIV epidemics.

Nahal B, *et al. BMJ Glob Health* 2024; 9:e012719. doi: 10.1136/bmjgh-2023-012719

Nagot, N.,Ouédraogo, A.,Foulongne, V.,Konaté, I.,Weiss, H. A.,Vergne, L.,Defer, M. C.,Djagbaré, D.,Sanon, A.,Andonaba, J. B.,Becquart, P.,Segondy, M.,Vallo, R.,Sawadogo, A.,Perre, P. van de,Mayaud, P.	2007	Reduction of HIV-1 RNA levels with therapy to suppress herpes simplex virus	West aFrica	RCT	136	New England Journal of Medicine	HIV shedding/transmission: effect of valcyclovir	valcyclovir significantly reduced genital and plasma HIV rna level in dually infected women not on HAART. May be useful in HIV control.

Kapiga, S. H.,Sam, N. E.,Bang, H. J.,Ni, Q. H.,Ao, T. T. H.,Kiwelu, I.,Chiduo, S.,Ndibe, U.,Seage, G., III,Coplan, P.,Shao, J.,Rosenberg, Z. F.,Essex, M.	2007	The role of herpes simplex virus type 2 and other genital infections in the acquisition of HIV-1 among high-risk women in Northern Tanzania	East Africa	cohort study	845		effect of HSV on HIV ACQUISITION	risk of HIV acquisition was increased in those with prevalent genital HSV (hazarad ratio 4.3) and even more elevated in incident HSV (HR .55). Hsv2 is a big part of HIV acquisition
Celum, C.,Wald, A.,Hughes, J.,Sanchez, J.,Reid, S.,Delany-Moretlwe, S.,Cowan, F.,Casapia, M.,Ortiz, A.,Fuchs, J.,Buchbinder, S.,Koblin, B.,Zwerski, S.,Rose, S.,Wang, J.,Corey, L.	2008	Effect of aciclovir on HIV-1 acquisition in herpes simplex virus 2 seropositive women and men who have sex with men: a randomised, double-blind, placebo-controlled trial	South America Southern Africa	RCT	3172		HIV ACQUISITION: effect of acyclovir	Acyclovir did NOT reduce HIV acquisition in HSV seropositive MSM and women

Dunne, E. F ; Whitehead, S ;. Sternberg, M ; Thepamnuay, S ; Leelawiwat, W ; McNicholl, J. M ; Sumanapun, S. Tappero, J. W ; Siriprapasiri, T ; Markowitz, L.	2008	Suppressive acyclovir therapy reduces HIV cervicovaginal shedding in HIV- and HSV-2-infected women, Chiang Rai, Thailand	East Asia			JAIDS, Journal of Acquired Immune Deficiency Syndromes		Acyclovir reduced HIV genital shedding and plasma viral load among HIV-1- and herpes simplex virus type 2-coinfected women

Watson-Jones, D.,Weiss, H. A.,Rusizoka, M.,Changalucha, J.,Baisley, K.,Mugeye, K.,Tanton, C.,Ross, D.,Everett, D.,Clayton, T.,Balira, R.,Knight, L.,Hambleton, I.,Goff, J. le,Belec, L.,Hayes, R.	2008	Effect of herpes simplex suppression on incidence of HIV among women in Tanzania	East Africa	RCT	821	New England Journal of Medicine	effect of HSV suppression on HIV acquisition (acyclovir)	acyclovir had NO effect on hi acquisition in tanzanian women with HSV
Mayaud, P.,Nagot, N.,Konaté, I.,Ouedraogo, A.,Weiss, H. A.,Foulongne, V.,Defer, M. C.,Sawadogo, A.,Segondy, M.,Perre, P. van de	2008	Effect of HIV-1 and antiretroviral therapy on herpes simplex virus type 2: a prospective study in African women	West Africa	cohort study	120	Sexually Transmitted Infections	effect of HIV and haart on HSV natural hx	those with HIV had more frequent HSV recurrence. Haart did not significantly help. Hsv therapy still recommended for those with HSV and on haart.

Abu-Raddad, Laith J.,Magaret, Amalia S.,Celum, Connie,Wald, Anna,Longini, Ira M., Jr.,Self, Steven G.,Corey, Lawrence	2008	Genital herpes has played a more important role than any other sexually transmitted infection in driving HIV prevalence in Africa	East and West Africa	modellin g study	999	PloS one	contribution of HSV to HIV (PAF as an outcome)	HSV2 has had a huge role in HIV epidemics in SSA. PAF up tp 35% in general populations in Africa. Hsv has facilitated spread of HIV through low risk groups. This supports need for a HSV 2 vaccine
Baeten, J. M.,Strick, L. B.,Lucchetti, A.,Whittington, W. L. H.,Sanchez, J.,Coombs, R. W.,Magaret, A.,Wald, A.,Corey, L.,Celum, C.	2008	Herpes simplex virus (HSV)-suppressive therapy decreases plasma and genital HIV-1 levels in HSV-2/HIV-1 coinfected women: a randomized, placebo-controlled, cross-over trial	South America	cross over RCT	20	Journal of Infectious Diseases	effect of valcyclovir on plasma and genital HIV (transmission)	valcyclovir was associated with reduced plasma and genital HIV. May be useful in reducing HIV transmission in dually infected
Anuradha, K.,Singh, H. M.,Gopal, K. V. T.,Rao, G. R. R.,Ramani, T. V.,Jyothi, Padmaja	2008	Herpes simplex virus 2 infection: a risk factor for HIV infection in heterosexuals	South Asia	case control study	100	Indian Journal of Dermatolo gy, Venereolog y & Leprology	comparing prevalence of HSV in HIV pos and neg	prevalence of HSV significantly higher in HIV pos

Glynn, J. R.,Crampin, A. C.,Ngwira, B. M. M.,Ndhlovu, R.,Mwanyongo, O.,Fine, P. E. M.	2008	Herpes simplex virus type 2 trends in relation to the HIV epidemic in northern Malawi	Southern Africa	cross sectional study	999	Sexually Transmitte d Infections	long term trends of HSV-2 compared to HIV /natural history/preval ence	HSV2 was likely to already be highly prevalent before HIV epidemic. High HSV prevalence not likely to be a consequence of HIV
Benjamin, R. J.,Busch, M. P.,Fang, C. T.,Notari, E. P.,Puren, A.,Schoub, B. D.,Tobler, L. H.,Hogrefe, W.,Heyns, A. P. du,Stramer, S. L.,Crookes, R. L.	2008	Human immunodeficiency virus-1 infection correlates strongly with herpes simplex virus-2 (genital herpes) seropositivity in South African and United States blood donations	Southern Africa	cross sectional study	625	Transfusio n	prevalence /epi	HSV 2 genital infection strongly correlated to HIV

Bollen, L. J. M.,Whitehead, S. J.,Mock, P. A.,Leelawiwat, W.,Asavapiriyanont, S.,Chalermchockchareonkit, A.,Vanprapar, N.,Chotpitayasunondh, T.,McNicholl, J. M.,Tappero, J. W.,Shaffer, N.,Chuachoowong, R.	2008	Maternal herpes simplex virus type 2 coinfection increases the risk of perinatal HIV transmission: possibility to further decrease transmission?	East Asia	longitudinal survey	307	AIDS	effect of maternal genital herpes on perinatal HIV transmission/shedding in dually infected people	maternal HSV infection associated with increased risk of perinatal HIV transmission. Increased risk of intrapartum HIV transmission in those who were shedding.
Moira A. McMahon‡, Janet D. Siliciano§, Jun Lai¶, Jun O. Liu‡,James T. Stivers‡, Robert F. Siliciano§¶, and Rahul M. Kohli§	2008	The Antiherpetic Drug Acyclovir Inhibits HIV Replication and Selects the V75I Reverse Transcriptase Multidrug Resistance Mutation*	sub saharan Africa	lab study	999	journal f biological chemistry	effect of acyclovir on ART resistance in HIV patients (V5721 gene)	episodic therapy with acyclovir may be associated with mutation in HIV reverse transcriptase genre leading to ART resistance. Clinicians may need to be cautious when starting dually infected people no t on haart on acyclovir

Cowan, F. M.,Pascoe, S. J.,Barlow, K. L.,Langhaug, L. F.,Jaffar, S.,Hargrove, J. W.,Robinson, N. J.,Bassett, M. T.,Wilson, D.,Brown, D. W. G.,Hayes, R. J.	2008	A randomised placebo-controlled trial to explore the effect of suppressive therapy with acyclovir on genital shedding of HIV-1 and herpes simplex virus type 2 among Zimbabwean sex workers	Southern Africa	RCT	125	Sexually Transmitte d Infections	effect of acyclovir on HIV and HSV genital shedding	acyclovir reduced HSV shedding but did not reduce HIV shedding. May be explained by low adherence or small sample size, larger clinical trials needed.
Nakubulwa, S.,Mirembe, F. M.,Kaye, D. K.,Kaddu-Mulindwa, D.	2009	Association between HSV-2 and HIV serostatus in pregnant women of known HIV serostatus attending Mulago Hospital antenatal clinic, Kampala, Uganda	East Africa	case control study	250	Journal of Infection in Developing Countries	compare HSV prevalence in HIV neg and pos pregnant women	odds of HSV positivity was almost 4 times higher in HIV pos (OR 3.7)

Sobngwi-Tambekou, J.,Taljaard, D.,Lissouba, P.,Zarca, K.,Puren, A.,Lagarde, E.,Auvert, B.	2009	Effect of HSV-2 serostatus on acquisition of HIV by young men: results of a longitudinal study in orange farm, South Africa	Southern Africa	longutu dinal study nested within a RCT	2974	Journal of Infectious Diseases	effect of HSV on HIV acquisition in men	prevalent HSV caused a increase in HIV acquisition (incident rate ratio = 3.3). Incident HSV had even greater effect (IRR= 7.0). Population attributable fraction of incident HIV caused by HSV was 27.8%. HSV does not alter the protective effect size of circumcision on HIV acquisition

Gray, R. H.,Serwadda, D.,Tobian, A. A. R.,Chen, M. Z.,Makumbi, F.,Suntoke, T.,Kigozi, G.,Nalugoda, F.,Iga, B.,Quinn, T. C.,Moulton, L. H.,Laeyendecker, O.,Reynolds, S. J.,Kong, X. R.,Wawer, M. J.	2009	Effects of genital ulcer disease and herpes simplex virus type 2 on the efficacy of male circumcision for HIV prevention: analyses from the Rakai trials	East Africa	RCT	2756	PLoS Medicine	effect of HSV on the effectiveness of circumcision in preventing HIV acquisition	HSV di not have a significant effect on the effectiveness of circumcision to prevent HIV acquisition, suggesting that the protective effect mainly comes from removal of HIV target cells in the foreskin tissue.
Johnson, K. E.,Sherman, M. E.,Ssempiija, V.,Tobian, A. A. R.,Zenilman, J. M.,Duggan, M. A.,Kigozi, G.,Serwadda, D.,Wawer, M. J.,Quinn, T. C.,Rabkin, C. S.,Gray, R. H.	2009	Foreskin inflammation is associated with HIV and herpes simplex virus type-2 infections in Rakai, Uganda	East Africa	cross sectional study nested within RCT	233	AIDS	effect of HSV andn HIV on foreskin inflammation	HSV and HIV increased foreskin inflammation separately and together to a greater extent. Foreskin inflammation may be implicated in HIV transmission and acquisition

Zuckerman, R. A.,Lucchetti, A.,Whittington, W. L. H.,Sánchez, J.,Coombs, R. W.,Magaret, A.,Wald, A.,Corey, L.,Celum, C.	2009	HSV suppression reduces seminal HIV-1 levels in HIV-1/HSV-2 co-infected men who have sex with men	South America	RCT	20	AIDS	effect of HSV therapy on seminal HIV shedding in dually infected men	valcyclovir therapy reduced seminal HIV shedding in dually infected msm in Peru. Another trial will be carried out to validate this with HIV transmission as an outcome
Mayaud, P.,LeGoff, J.,Weiss, H. A.,Grésenguet, G.,Nzambi, K.,Bouhlal, H.,Frost, E.,Pépin, J.,Malkin, J. E.,Hayes, R. J.,Mabey, D. C. W.,Bélec, L.	2009	Clinical and virologic response to episodic acyclovir for genital ulAfrican women with herpes ulcers: a randomized placebo-controlled trial	West and central Africa	RCT	441	Journal of Infectious Diseases	effect of episodic acyclovir on HIV transmission parameters in dually infected	episodic acyclovir reduced cervicovaginal HSV shedding but did not reduce genital and plasma HIV rna loads. Trials of longer duration of therapy should be done

Freeman, Esther E.,White, Richard G.,Bakker, Roel,Orroth, Kate K.,Weiss, Helen A.,Buvé, Anne,Hayes, Richard J.,Glynn, Judith R.	2009	Population-level effect of potential HSV2 prophylactic vaccines on HIV incidence in sub-Saharan Africa	East West and Southern African cities	modelling study based on population based data	999	Vaccine	effect of HSV vaccine on HIV incidence	HSV vaccine may have substantial effect on HIV incidence (theoretical). More should be done to make one available sooner
Baggaley, Rebecca F.,Griffin, Jamie T.,Chapman, Ruth,Hollingsworth, T. Déirdre,Nagot, Nicolas,Delany, Sinead,Mayaud, Philippe,de Wolf, Frank,Fraser, Christophe,Ghani, Azra C.,Weiss, Helen A.	2009	Estimating the public health impact of the effect of herpes simplex virus suppressive therapy on plasma HIV-1 viral load	West and Southern Africa	modelling study	999	AIDS (London, England)	effect of HSV therapy on vira load	limited effect of HSV therapy on HIV viral load

Baltzer, H.,Chege, D.,Rebbapragada, A.,Wachihi, C.,Shin, L. Y. Y.,Kimani, J.,Ball, T. B.,Jaoko, W.,Plummer, F. A.,Kaul, R.	2009	Relative HIV resistance in Kenyan sex workers is not due to an altered prevalence or mucosal immune impact of herpes simplex virus type 2 infection	East Africa	cross sectional study	139	Current HIV Research	investigating whether HSV was implicated in relative HIV resistance in FSW in Kenya	relative HIV resistance was not likely to be due to decreased HSV prevalence or mucosal immune impact of genital HSV
Mahiane, S. G.,Legeai, C.,Taljaard, D.,Latouche, A.,Puren, A.,Peillon, A.,Bretagnolle, J.,Lissouba, P.,Nguéma, E. P. N.,Gassiat, E.,Auvert, B.	2009	Transmission probabilities of HIV and herpes simplex virus type 2, effect of male circumcision and interaction: a longitudinal study in a township of South Africa	Southern Africa	modelling study using longitudinal study data	3274	AIDS	the effect of HSV on female to male sexual transmission of HIV (and vice cerca).	HSV in either partner increased female to male risk of transmission of HIV (RR 3.0). HIV in either partner increased female to male risk of transmission of HSV (RR 2.5).

Tobian AAR, Ssempijja V, Kigozi G, Oliver AE, Serwadda D, Makumbi F	2009	Incident HIV and herpes simplex virus type 2 infection among men in Rakai, Uganda.	Southern Africa			AIDS		
Tanton, C.,Weiss, H. A.,Rusizoka, M.,LeGoff, J.,Changalucha, J.,Baisley, K.,Mugeye, K.,Everett, D.,Belec, L.,Clayton, T. C.,Ross, D. A.,Hayes, R. J.,Watson-Jones, D.	2010	Long-term impact of acyclovir suppressive therapy on genital and plasma HIV RNA in Tanzanian women: a randomized controlled trial	East Africa	RCT	484	Journal of Infectious Diseases	long term effect of acyclovir on HIV genital and plasma load in dually infected women	acyclovir did not show significant effect on HIV shedding parameters

Nahal B, et al. BMJ Glob Health 2024; 9:e012719. doi: 10.1136/bmjgh-2023-012719

LeGoff, J.,Tanton, C.,Delaugerre, C.,Weiss, H. A.,Changalucha, J.,Ross, D. A.,Mugeye, K.,Belec, L.,Hayes, R. J.,Watson-Jones, D.	2010	No selection of nucleoside reverse transcriptase inhibitor resistance associated mutations by acyclovir suppressive therapy in herpes simplex virus-2/HIV-1 dually infected persons	East Africa	cross sectional study nested within RCT	21		does acyclovir induce mutations that cause NRTI resistance in dually infected?	acyclovir did not cause the mutations in dually infected women from Tanzania who had received bd acyclovir 400mg for 12 months
C. Celum, A. Wald, J.R. Lingappa, A.S. Magaret, R.S. Wang, N. Mugo, A. Mujugira, J.M. Baeten, J.I. Mullins, J.P. Hughes, E.A. Bukusi, C.R. Cohen	2010	Acyclovir and Transmission of HIV-1 from Persons Infected with HIV-1 and HSV-2	Southern Africa	RCT	3408	new England Journal of Medicine	effect of standard dose acyclovir on forward transmission of HIV in dually infected	although acyclovir reduced plasma HIV rna , it did not reduce HIV transmission in heterosexual HIV discordant couples
Munjoma, M. W.,Mapingure, M. P.,Stray-Pedersen, B.	2010	Risk factors for herpes simplex virus type 2 and its association with HIV among pregnant teenagers in Zimbabwe	Southern Africa		176	Sexual Health	prevalence /epi	prevalence of HSV much higher in HIV infected (OR: 7.9)

Nahal B, et al. BMJ Glob Health 2024; 9:e012719. doi: 10.1136/bmjgh-2023-012719

Vickerman, P.,Devine, A.,Foss, A. M.,Delany-Moretlwe, S.,Mayaud, P.,Meyer-Rath, G.	2011	The cost-effectiveness of herpes simplex virus-2 suppressive therapy with daily aciclovir for delaying HIV disease progression among HIV-1-infected women in South Africa	Southern Africa	modelling study using primary healthcare data, demographic data and participant data from a	999	Sexually Transmitted Diseases	cost effectivness (cost per lie year gained) of acyclovir daily in dually infected	acyclovir daily could be a cost effective strategy to extend women's lives who are dually infected and not started art. This is assuming acyclovir becomes cheaper and widely available at that price.
Charpentier, C.,Koyalta, D.,Ndinaromtan, M.,Tchobkréo, B.,Jenabian, M. A.,Day, N.,Si-Mohamed, A.,Weiss, H.,Bélec, L.	2011	Distribution of HIV-1 and HSV-2 epidemics in Chad revealing HSV-2 hot-spot in regions of high-risk HIV spread	central Africa	population based survey (national)	548	Journal of Infection in Developing Countries	national distribution of HIV and HSV in chad-prevalence	significant association between prevalence of HIV and HSV

Baeten, J. M.,Lingappa, J.,Beck, I.,Frenkel, L. M.,Pepper, G.,Celum, C.,Wald, A.,Fife, K. H.,Were, E.,Mugo, N.,Sanchez, J.,Essex, M.,Makhema, J.,Kiarie, J.,Farquhar, C.,Corey, L.	2011	Herpes simplex virus type 2 suppressive therapy with acyclovir or valacyclovir does not select for specific HIV-1 resistance in HIV-1/HSV-2 dually infected persons	South America, Southern Africa, and East Africa	modellin g study using RCT data (in vivo)	168	Journal of Infectious Diseases	does acyclovir or valcyclovir induce mutations that cause NRTI resistance in dually infected? (V751)	in vivo studies suggest that acyclovir and valcyclovir do NOT cause V751 mutations at normal dose (contrary to what mc mahon found in in vitro studies)
Mugwanya, K.,Baeten, J. M.,Mugo, N. R.,Irungu, E.,Ngure, K.,Celum, C.	2011	High-dose valacyclovir HSV-2 suppression results in greater reduction in plasma HIV-1 levels compared with standard dose acyclovir among HIV-1/HSV-2 coinfected persons: a randomized, crossover trial	East Africa	cross over RCT	32	Journal of Infectious Diseases	high dose valcyclovir vs standard dose acyclovir in reducing plasma HIV rna in dually infected	high dose valcyclovir reduced plasma HIV significantly more than standard dos acyclovir- high dose valcyclovir may be considered in the effort to delay clinical course of HIV and reduce HIV infectivity

Foss, A. M.,Vickerman, P. T.,Mayaud, P.,Weiss, H. A.,Ramesh, B. M.,Reza-Paul, S.,Washington, R.,Blanchard, J.,Moses, S.,Lowndes, C. M.,Alary, M.,Watts, C. H.	2011	Modelling the interactions between herpes simplex virus type 2 and HIV: implications for the HIV epidemic in Southern India	South Asia (India)			Sexually Transmitte d Infections	modelling HSV and HIV interaction: contribution of HSV-2 to HIV and modelled effect of a 'perfect' HSV suppressive treatment. PAF as a outcome	HSV-2 plays big role in transmission and acquisition of HIV in South India. Continuous suppressive HSV therapy may be useful in FSW and clients in South India. 35.5% of HIV incidence in FSW due to HSV-2.
Tanton, Clare,Weiss, Helen A.,LeGoff, Jerome,Changalucha, John,Clayton, Tim C.,Ross, David A.,Belec, Laurent,Hayes, Richard J.,Watson-Jones, Deborah	2011	Patterns of herpes simplex virus shedding over 1 month and the impact of acyclovir and HIV in HSV-2-seropositive women in Tanzania	East Africa	RCT	78	Sexually transmitte d infections	effect of acyclovir on HIV shedding in dually infected	acyclovir did not significantly reduce HIV shedding in dually infected

Nahal B, *et al. BMJ Glob Health* 2024; 9:e012719. doi: 10.1136/bmjgh-2023-012719

Camara, M.,Seydi, M.,Dieye, T. N.,Sow, P. S.,Mboup, S.,Kestens, L.,Jennes, W.	2012	Association between herpes simplex virus type 2 and HIV-1 in a population of married couples from Dakar, Senegal	West Africa	cross sectional study	208	International Journal of STD & AIDS	prevalence of HSV in different HIV couple status	strong association nin prevalence of HIV and HSV (OR 4.6) but no difference in HSV prevalence between HIV discordant and HIV concordant positive suggesting HSV is not a RF for HIV in this population
Ghebremichael, Musie,Habtzgi, Desale,Paintsil, Elijah	2012	Deciphering the epidemic synergy of herpes simplex virus type 2 (HSV-2) on human immunodeficiency virus type 1 (HIV-1) infection among women in sub-Saharan Africa	East arica	cross sectional study random sample	1418	BMC research notes	prevalence of HSV and HIV +association	HSV positive women more likely to be HIV positive (OR: 2.7)
Drake, A. L.; Roxby, A. C.; Ongecha-Owuor, F.; Kiarie, J.; John-Stewart, G.; Wald, A.; Richardson, B. A.; Hitti, J.; Overbaugh, J.;	2012	Valacyclovir suppressive therapy reduces plasma and breast milk HIV-1 RNA levels during pregnancy and postpartum: a randomized trial	East Africa	RCT	148	Journal of Infectious Diseases		

Appendix 1 (paper C)_Included Articles 100 of 120

Emery, S.; Farquhar, C.								
Reynolds, S. J.,Makumbi, F.,Newell, K.,Kiwanuka, N.,Ssebbowa, P.,Mondo, G.,Boaz, I.,Wawer, M. J.,Gray, R. H.,Serwadda, D.,Quinn, T. C.	2012	Effect of daily aciclovir on HIV disease progression in individuals in Rakai, Uganda, co-infected with HIV-1 and herpes simplex virus type 2: a randomised, double-blind placebo-controlled trial	East Africa	RCT	440	Lancet Infectious Diseases	effect of acyclovir of hi disease progression (CD4 count as outcome)	acyclovir standard dose reduced rate of HIV disease progression in those with high baseline viral load
Roxby, A. C.,Drake, A. L.,Ongecha-Owuor, F.,Kiarie, J. N.,Richardson, B.,Matemo, D. N.,Overbaugh, J.,Emery, S.,John-Stewart, G. C.,Wald, A.,Farquhar, C.	2012	Effects of valacyclovir on markers of disease progression in postpartum women co-infected with HIV-1 and herpes simplex virus-2	East Africa	RCT	148	PLoS ONE	effect of valcyclovir on HIV disease progression (CD4 and viral load as outcome measures)	valcyclovir was associated with greater increases in CD4 and smaller increases in viral load. Could be useful for pregnant women and delay need for ART

Forhan, S. E.,Dunne, E. F.,Sternberg, M. R.,Whitehead, S. J.,Leelawiwat, W.,Thepamnuay, S.,Chen, C.,Evans-Strickfaden, T. T.,McNicholl, J. M.,Markowitz, L. E.	2012	HIV-1 and herpes simplex virus type-2 genital shedding among co-infected women using self-collected swabs in Chiang Rai, Thailand	East Asia	longitudinal survey	67	International Journal of STD & AIDS	self collected samples for HIV and HSV shedding in dually infected	SCS useful for future studies in dually infected since it allows more frequent sampling of more people.
Mawak JD, Dashe N, Atseye AB, Agabi YA, Zakeri H	2012	Seroprevalence and co-infection of herpes simplex virus type 2 and human immunodeficiency virus in Nigeria	West Africa	Cross sectional survey		Shiraz E Medical Journal		

Janbakhash, A.,Mansouri, F.,Vaziri, S.,Sayad, B.,Afsharian, M.,Abedanpor, A.	2012	Seroepidemiology of herpes simplex virus type 2 (HSV2) in HIV infected patients in Kermanshah-Iran	South Asia	cross sectional study	335	Caspian Journal of Internal Medicine (CJIM)	prevalence	HSV prevalence in HIV positive people was low (6%) therefore HSV interventions arentnecessary for HIV management in this part of iran
Todd, J.,Riedner, G.,Maboko, L.,Hoelscher, M.,Weiss, H. A.,Lyamuya, E.,Mabey, D.,Rusizoka, M.,Belec, L.,Hayes, R.	2013	Effect of genital herpes on cervicovaginal HIV shedding in women co-infected with HIV and HSV-2 in Tanzania	East Africa	longitudinal survey	131	PLoS ONE	effect of HSV on HIV shedding in co infected	data suggests HSV is associated with increased HIV sheddig and hence transmissability (nor synonymous tho no). Data suggests that quantitiy o HSV genital shedding is a more prominent determinant of gential HIV shedding than presence of herpetic lesions.

Biraro, S.,Kamali, A.,White, R.,Karabarinde, A.,Ssendagala, J. N.,Grosskurth, H.,Weiss, H. A.	2013	Effect of HSV-2 on population-level trends in HIV incidence in Uganda between 1990 and 2007	East Africa	retrospe ctive cohort	13,021	Tropical Medicine and Internation al Health	long term effect of population level HSV on population level HIV incidence: epi	high PAF (60% in males and 70% in females) in this pop. However no association found between trends of HSV and HIV but this may be due to HIV incidence not changing much across the time.
Phiri, S.,Zadrozny, S.,Weiss, H. A.,Martinson, F.,Nyirenda, N.,Chen, ChengYen,Mille r, W. C.,Cohen, M. S.,Mayaud, P.,Hoffman, I. F.	2013	Etiology of genital ulcer disease and association with HIV infection in Malawi	Southern Africa	cross sectional study	422	Sexually Transmitte d Diseases	prevalence /epi	Hiv prevalence strongly associated with HSV . Hiv more prevalent in recurrent HIV rather than first episode

Muiru, Anthony N.,Guthrie, Brandon L.,Bosire, Rose,Merkel, Michele,Liu, Amy Y.,Choi, Robert Y.,Lohman-Payne, Barbara,Gatuguta, Ann,Mackelprang, Romel D.,Kiarie, James N.,Farquhar, Carey	2013	Incident HSV-2 infections are common among HIV-1-discordant couples	East Africa	prospective cohort study	469	The Journal of infectious diseases	effct of HIV on HSV incidence	highi prevalenc and incidence of HSV in HIV discordant couples in Kenya. Suggests that HSV therapy may be useful in reucing HSV and HIV transmission in high risk groups.
Celum, C.,Morrow, R. A.,Donnell, D.,Hong, T.,Hendrix, C. W.,Thomas, K. K.,Fife, K. H.,Nakku-Joloba, E.,Mujugira, A.,Baeten, J. M.	2014	Daily oral tenofovir and emtricitabine-tenofovir preexposure prophylaxis reduces herpes simplex virus type 2 acquisition among heterosexual HIV-1-uninfected men and women: a subgroup analysis of a randomized trial	East Africa	sub analysis of a RCT	1498	Annals of Internal Medicine	effect of daily PrEP on HSV incidence	daily oral tenofovir based PrEP reduced rate of HSV acquisition in iheterosexaul men and women. This is an added benefit of tenofovir based prep on top of HIV prevention

Nahal B, et al. BMJ Glob Health 2024; 9:e012719. doi: 10.1136/bmjgh-2023-012719

Chamoun-Emanuelli, Ana M.,Bobardt, Michael,Moncla , Bernard,Mankowski, Marie K.,Ptak, Roger G.,Gallay, Philippe,Chen, Zhilei	2014	Evaluation of PD 404,182 as an anti-HIV and anti-herpes simplex virus microbicide	conducted in US with view of developing countries	in vitro study	999	Antimicrobial agents and chemotherapy	evaluation of new microbicide PD in treating HIV and HSV	was previously known to kill HIV but study found that is it also effective in destroying HSV. Other tests suggest it is suitable for further investigation to becoming licensed virucidal
Kenyon C, Buyze J, Colebunders R.	2014	Classification of incidence and prevalence of certain sexually transmitted infections by world regions	Multi			International journal of infectious diseases	prevalence /epi	
Kenyon, C. R.,Osbak, K.,Buyze, J.	2014	The prevalence of HIV by ethnic group is correlated with HSV-2 and syphilis prevalence in Kenya, South Africa, the United Kingdom,	East and Southern Africa	cross sectional study	>20,000	Interdisciplinary Perspectives on Infectious Diseases	prevalence /epi	prev of HSV more common in HIV positive

		and the United States						
Meque, I.,Dubé, K.,Feldblum, P. J.,Clements, A. C. A.,Zango, A.,Cumbe, F.,Chen, P. L.,Ferro, J. J.,Wijgert, J. H. van de	2014	Prevalence, incidence and determinants of herpes simplex virus type 2 infection among HIV-seronegative women at high-risk of HIV infection: a prospective study in beira, Mozambique	Southern Africa	prospective cohort study	411	PLoS ONE	risk of HIV in HSV pts	no association between beingHSV positive at baseline and acquiring HIV
Ankamma, A.,Nagaraja, B.,Radha, P.,Reddy, B. K.,Venkatarao, B.	2014	Study of seroprevalence of HSV-2 among HIV seropositive individuals at S.V.R.R.G.G.H Tirupati	South Asia	cross sectional study	182	International Journal of Pharmaceutical Research and Bio-Science	prev	high prevalence in caes compared to control (sig)
Nakku-Joloba E, Kambugu F, Wasubire J, Kimeze J, Salata R, Albert JM	2014	Sero-prevalence of herpes simplex type 2 virus (HSV-2) and HIV infection in Kampala, Uganda	East Africa	cross sectional study		African Health Sciences	prev	

Nahal B, et al. BMJ Glob Health 2024; 9:e012719. doi: 10.1136/bmjgh-2023-012719

Behling, Juliane,Chan, Adrienne K.,Zeh, Clement,Nekesa, Carolyne,Heinzerling, Lucie	2015	Evaluating HIV Prevention Programs: Herpes Simplex Virus Type 2 Antibodies as Biomarker for Sexual Risk Behavior in Young Adults in Resource-Poor Countries	East Africa	cross sectional study	70,000	PloS one	the use of HSV incidence as a proxy marker for sexual risk behaviour and HIV infection (because HIV incidence is much lower and therefore a huge sample size would be required)	the anticipated association between HSV serostatus and sexual risk behaviour was not found. Likely due to social bias from the interviews on sexual behaviour. Still some potential though for HSV serostatus as a marker of HIV
Saramma, Jacob,Thatchinamoorthy, Gopal,Sivasangeetha, Kanagasabai,Anitha, Durairaj,Kamala, Sushi,Ganesan, Arumugam	2015	Herpes Simplex Virus 2 infection in HIV-seropositive individuals in Tamil Nadu, India	South Asia	longitudinal survey	273	International Journal of Medical Science and Public Health	prevalence of HSV in HIV pos pts	HSV significantly more prevalent in HIV positive . More so in women than men.
Okango, E.,Mwambi, H.,Ngesa, O.,Achia, T.	2015	Semi-parametric spatial joint modeling of HIV and HSV-2 among women in Kenya	East Africa	modelling study	999	PLoS ONE	joint spatial modelling the relationship between HIV and HSV	positive significant spatial relationship between HIV and HSV

Bharathi, V. C.,Anaparty, Usharani	2015	Sero evaluation of coinfection of HIV and HSV-2	South Asia	cross sectional study	130	Journal of Pharmaceutical and Scientific Innovation (JPSI)	prevalence of HSV in HIV pos pts	high HSV prevalence in HIV positive (no control group though)
Low, A. J.,Nagot, N.,Weiss, H. A.,Konate, I.,Kania, D.,Segondy, M.,Meda, N.,Perre, P. van de,Mayauda, P.	2016	Herpes simplex virus type-2 cervicovaginal shedding among women living with HIV-1 and receiving antiretroviral therapy in Burkina Faso: an 8-year longitudinal study	West Africa	cohort study	236	Journal of Infectious Diseases	effect of ART on vaginal shedding of HSV in dually infected	ART was associated with reduced genital HSV shedding over the 8 years.
Thienkrua, W.,Todd, C. S.,Chonwattana, W.,Wimonsate, W.,Chaikummao, S.,Varangrat, A.,Chitwarakorn, A.,Griensven, F. van,Holtz, T. H.	2016	Incidence of and temporal relationships between HIV, herpes simplex II virus, and syphilis among men who have sex with men in Bangkok, Thailand: an observational cohort	East Asia	cohort study	960	BMC Infectious Diseases	temporal relationship between HIV and HSV	high incidence of HIV and HSV in this cohort. Timeframe (60m) was not enough to assess the temporal relationships between HSV and HIV

Nahal B, et al. BMJ Glob Health 2024; 9:e012719. doi: 10.1136/bmjgh-2023-012719

Daniels, B.,Wand, H.,Ramjee, G.	2016	Prevalence of Herpes Simplex Virus 2 (HSV-2) infection and associated risk factors in a cohort of HIV negative women in Durban, South Africa	Southern Africa	cohort study withi a RCT	2236	BMC Research Notes	prevalence of HSV and HIV incidence	very high baseline HSV prevalence (65%). Hiv incidence significantly higher in those with prevalent HSV
Nwadike, V. U.,Anaedobe, C. G.,Azeez, R. A.,Jinadu, S. S.,Chigozie, J. O.	2018	Atypical presentation of genital herpes in a retroviral disease patient on highly active anti-retroviral therapy	West Africa	case study	1	African Journal of Clinical and Experiment al Microbiolo gy	is haart associated with increased risk of acyclovir resistant genital herpes and chrionic genita herpes?	HIV positive people on haart may have more severe forms of HSV and clinicalns should consider offering acyclovir
Kenyon, C. R.	2018	HIV prevalence by ethnic group covaries with prevalence of herpes simplex virus-2 and high-risk sex in Uganda: an ecological study	East Africa	cross sectional study populati on based	11454	PLoS ONE	association between HIV prevalence and HSV prevalece	highlyl correlated

Adejumo, B. I. G.,Oronsaye, F. E.,Drisu, U. I.,Adebowale, M. O.,Oke, O. M.,Dimkpa, U.,Omosor, K. I.,Abdulrahman, O. N.,Ukatu, E. N.,Michael, E. A.	2018	The level of CD4+ T cell count among reproductive age women coinfected with human immune virus, hepatitis surface antigen and herpes simplex virus in Kogi state, Nigeria	West Africa	cross sectional study	342	Health	is CD4 count affected by HSV and HIV coinfection	no evidence for this
Mohraz, M.,Aghakhani, A.,Moayedi-Nia, S.,Banifazl, M.,Janbakhsh, A.,Mamishi, S.,Karami, A.,Bavand, A.,Mirzapour, P.,Ramezani, A.	2018	No role of herpes simplex virus type 2 (HSV-2) infection on HIV progression in naïve HIV patients	South Asia	cohort study	201	Iranian Biomedical Journal	effect of HSV in HIV disease progression	no evidence that hcoinfection accelerates HIV progression
Lima, L. R. P.,Almeida, N. A. A. de,Guterres, A.,Pilotto, J. H.,Niel, C.,Paula, V. S. de	2018	Novel variants of human herpesvirus 2 from Brazilian HIV-1 coinfected subjects	South America	cross sectional study	6	Memórias do Instituto Oswaldo Cruz	new variants of HSv-2	new variant- should be further investigated

Bradley, J.,Floyd, S.,Piwowar-Manning, E.,Laeyendecker, O.,Young, A.,Bell-Mandla, N.,Bwalya, J.,Bock, P.,Fidler, S.,Ayles, H.,Hayes, R. J.	2018	Sexually transmitted bedfellows: exquisite association between HIV and herpes simplex virus type 2 in 21 communities in Southern Africa in the HIV Prevention Trials Network 071 (PopART) study	Southern Africa	RCT	38691	Journal of Infectious Diseases	effect of HSV-2 on HIV inciedence	HIV incidence much more common in HSV-2 positive people (Or 6.6). Strong association between the prevalence of both on an individual and community level. Hsv interventions should be incorporated into HIV prevention
Madebe, R.,Kiwelu, I.,Ndaro, A.,Francis, F.,Baraka, V.,Theilgaard, Z.,Katzenstein, T.	2020	Herpes Simplex virus type 2 seroprevalence and risk factors among adolescents and youth with HIV-1 in Northern, Tanzania	East Africa	cross sectional study	180	Journal of Infection in Developing Countries	prevalence of HSV in HIV pos pts	oveall prevalence of HSV amongst the HIV pos youths was 18% but varried a lot by age- those between 20-14 had a six fold higher odds of being HSV pos than for 10-14 yr olds . Age specific interventions are important

Mathematical modelling

Authors	Year	Title	Geographical area of study participants	Study topic	Main findings

Nahal B, *et al. BMJ Glob Health* 2024; 9:e012719. doi: 10.1136/bmjgh-2023-012719

Korenromp, Eline L.,Bakker, Roel,De Vlas, Sake J.,Robinson, N. Jamie,Hayes, Richard,Habbema, J. Dik F.	2002	Can behaviour change explain increases in the proportion of genital ulcers attributable to herpes in sub-Saharan Africa? A simulation modeling study	East Africa (Tanzania)	proportion of GUd attributable to HSV: the effect of HIV and behaviour change on HSV incidence	increased proportion of GUD attributable to HSV likely to be artefact of increased detection etc not absolute (not caused by hv epidemic). Behavioural change in response to HIV epidemic (more condom use, less transactional sex) may have significant effect on increasing relative HSV cases (less of other treatable STIs/better treatment)
Korenromp, E. L.,Bakker, R.,Vlas, S. J. de,Gray, R. H.,Wawer, M. J.,Serwadda, D.,Sewankambo, N. K.,Habbema, J. D. F.	2002	HIV dynamics and behaviour change as determinants of the impact of sexually transmitted disease treatment on HIV transmission in the context of the Rakai trial	East Africa (India)	does HIV dynamics and behaviour change affect the impact of HSV treatment on HIV transmission	impact of HSV treatment on HIV transmission decreases with time in the first decade of an epidemic. in the second decade of a simulated epidemic the behaviour change may be a greater determinant of the impact of HSV therapy on HIV transmission than the stage of the epidemic. Timing and behaviour change are important things to consider before strating a HSV treatment for HIV transmission.
Freeman, E. E.,Orroth, K. K.,White, R. G.,Glynn, J. R.,Bakker, R.,Boily, M. C.,Habbema, D.,Buvé, A.,Hayes, R.	2007	Proportion of new HIV infections attributable to herpes simplex 2 increases over time: simulations of the changing role of sexually transmitted infections in sub-	East Africa, West Africa, Southern Africa (SSA)	impact of HSV on HIV incidence- population attributable fraction (PAF) as a outcome	PAF of HIV caused by HSV incresed with time in the modelled epidemic. Hsv has only small impact on nHIV incidence in early epidemic but a larger impact as the epidemic deelops and in core groups. Hsv interventions may be important later in epidemic of HIV.

		Saharan African HIV epidemics			
Abu-Raddad, L. J.,Magaret, A. S.,Celum, C.,Wald, A.,Longini, I. M., Jr.,Self, S. G.,Corey, L.	2008	Genital herpes has played a more important role than any other sexually transmitted infection in driving HIV prevalence in Africa	East Africa, West Africa, Southern Africa (SSA)	role of HSV in driving HIV epidemic	HSV played a bigger role than any other STI in driving the Africa HIV epidemic. PAF (population attributable fraction) of HIV caused by HSV estimated to be over 25% in Kisumu Kenya. Support need for an HSV vaccine
Vickerman, P.,Ndowa, F.,Mayaud, P.	2008	Modelling the cost per ulcer treated of incorporating episodic treatment for HSV-2 into the syndromic algorithm for genital ulcer disease	sub saharan Africa input	cost effectiveness of incorporating acyclovir into syndromic management of GUD	incorporating acyclovir into syndromic management may be an affordable way of managing HSV correctly in low resource settings,. Prevalence of HSV-2 in the area may help determine whether or not to do this.
White, R. G.,Freeman, E. E.,Orroth, K. K.,Bakker, R.,Weiss, H. A.,O'Farrell, N.,Buvé, A.,Hayes, R. J.,Glynn, J. R.	2008	Population-level effect of HSV-2 therapy on the incidence of HIV in sub-Saharan Africa	West and East Africa	effect of HSV therapeutics on HIV incidence (acquisition and transmission)- epi	HSV therapy could have a positive impact on HIV incidence on population level in more concentrated epidemics, but only with high coverage long duration of therapy. Ideally a vaccine is needed. PAF of HSV on HIV is higher in more concentrated epidemics.

Foss, A. M.,Vickerman, P. T.,Chalabi, Z.,Mayaud, P.,Alary, M.,Watts, C. H.	2009	Dynamic modeling of Herpes simplex virus type-2 (HSV-2) transmission: issues in structural uncertainty	South Asia (India)	transmission modelling: structural issues	simple model is as accurate as complex model in developed HSV epidemics (>60% prev)
Freeman, E. E.,White, R. G.,Bakker, R.,Orroth, K. K.,Weiss, H. A.,Buvé, A.,Hayes, R. J.,Glyn, J. R.	2009	Population-level effect of potential HSV2 prophylactic vaccines on HIV incidence in sub-Saharan Africa	East West and Southern African cities	effect of HSV vaccine on HIV incidence	HSV vaccine may have substantial effect on HIV incidence (theoretial). More should be done to make one available sooner
Mahiane, S. G.,Legeai, C.,Taljaard, D.,Latouche, A.,Puren, A.,Peillon, A.,Bretagnolle, J.,Lissouba, P.,Nguéma, E. P. N.,Gassiat, E.,Auvert, B.	2009	Transmission probabilities of HIV and herpes simplex virus type 2, effect of male circumcision and interaction: a longitudinal study in a township of South Africa	South Africa	what is the rate of female to male sexual transmission of HSV and how is it affected by male circumcision?	HSV 2 increased risak of female to male sexual transmission of HIV. HIV increased risk of sexual transmission of HSV-2. MC reduced risk of HSV-2 transmission. Estimated tansmission rate of HSV-2 : 0.0067 in Africa
Alsallaq, R. A.,Schiffer, J. T.,Longini, I. M., Jr.,Wald, A.,Corey,	2010	Population level impact of an imperfect prophylactic	developing country	population and individual effects of HSV-2 vaccine	pre exposure HSV 2 vaccine would only have a small effect on HSV 2 incidence. They would hav emuch greater public health benefits in developing countries where prevalence is higher.

Nahal B, et al. BMJ Glob Health 2024; 9:e012719. doi: 10.1136/bmjgh-2023-012719

L.,Abu-Raddad, L. J.		vaccine for Herpes simplex virus-2			
Vickerman, P.,Devine, A.,Foss, A. M.,Delany-Moretlwe, S.,Mayaud, P.,Meyer-Rath, G.	2011	The cost-effectiveness of herpes simplex virus-2 suppressive therapy with daily aciclovir for delaying HIV disease progression among HIV-1-infected women in South Africa	Southern Africa	cost effectiveness of HSV suppressive therapy on HIV progression in co infected	HSV suppressive therapy could be a cost effective way to reduce HIV progression before ART initiation , this is based on cheap acyclovir being available
Foss, A. M.,Vickerman, P. T.,Mayaud, P.,Weiss, H. A.,Ramesh, B. M.,Reza-Paul, S.,Washington, R.,Blanchard, J.,Moses, S.,Lowndes, C. M.,Alaryj, M.,Watts, C. H.	2011	Modelling the interactions between herpes simplex virus type 2 and HIV: implications for the HIV epidemic in Southern India	South Asia (India)	modelling HSV and HIV interaction: contribution of HSV-2 to HIV and modelled effect of a 'perfect' HSV suppressive treatment. PAF as a outcome	HSV-2 plays big role in transmission and acquisition of HIV in South India. Continuous suppressive HSV therapy may be useful in FSW and clients in South India. 35.5% of HIV incidence in FSW due to HSV-2.

Nahal B, *et al. BMJ Glob Health* 2024; 9:e012719. doi: 10.1136/bmjgh-2023-012719

Arora P, Nagelkerke N, Sgaier SK, Kumar R, Dhingra N, Jha P	2011	HIV, HSV-2 and syphilis among married couples in India: patterns of discordance and concordance	South Asia	Modelling concordance and discordance in couples in India	
Kacker, S.,Frick, K. D.,Quinn, T. C.,Gray, R. H.,Tobian, A. A. R.	2013	Financial implications of male circumcision scale-up for the prevention of HIV and other sexually transmitted infections in a sub-Saharan African community	East Africa (India)	effect of MC on STIs including HSV-2	STIs other than HIV (including HSV-2) also contribute to cost savings associated with increased MC rate.
Todd J, Riedner G, Maboko L, Hoelscher M, Weiss HA, Lyamuya E	2013	Effect of genital herpes on cervicovaginal HIV shedding in women co-infected with HIV and HSV-2 in Tanzania	East Africa	effect of HSV on HIV shedding in co infected	data suggests HSV is associated with increased HIV sheddig and hence transmissability (nor synonymous tho no). Data suggests that quantitiy o HSV genital shedding is a more prominent determinant of gential HIV shedding than presence of herpetic lesions.
Terris-Prestholt F, Foss AM, Cox AP, Heise L, Meyer-	2014	Cost-effectiveness of tenofovir gel in urban South Africa: model	Southern Africa	Cost effectiveness models	

Nahal B, *et al. BMJ Glob Health* 2024; 9:e012719. doi: 10.1136/bmjgh-2023-012719

Rath G, Delany-Moretlwe S		projections of HIV impact and threshold product prices			
Jewell, B. L.,Cremin, I.,Pickles, M.,Celum, C.,Baeten, J. M.,Delany-Moretlwe, S.,Hallett, T. B.	2015	Estimating the cost-effectiveness of pre-exposure prophylaxis to reduce HIV-1 and HSV-2 incidence in HIV-serodiscordant couples in South Africa	South Africa	cost effectiveness of daily tenofovir PrEP for reducing HSV2 incidence	the protective effects of PrEP against HSV-2 does not significantly improve the cost effectiveness of PrEP. But the modest improvement may be an additional factor that makes this more appealing as a HIV intervention.
Ghebremichael, M.	2015	Joint modelling of correlated binary outcomes: HIV-1 and HSV-2 co-infection	East Africa (Tanzania)	HIV and HSv 2 coinfection usinf joint response model that accounts for the inter-dependence of both infections	joint response model is more accurate ans less biased than modeling both diseases separately
Okango, E.,Mwambi, H.,Ngesa, O.,Achia, T.	2015	Semi-parametric spatial joint modeling of HIV and HSV-2 among women in Kenya	East Africa	joint spatial modelling the relationship between HIV and HSV	positive significant spatial relationship between HIV and HSV
Okango, E.,Mwambi, H.,Ngesa, O.	2016	Spatial modeling of HIV and HSV-2 among women in Kenya with	East Africa	relaxing assumptions in spatial modelling of HSV	spatial variation in determinants of HSV occurred. using models to relax assumptions can result in more accurate results and interpretations.

Nahal B, *et al. BMJ Glob Health* 2024; 9:e012719. doi: 10.1136/bmjgh-2023-012719

		spatially varying coefficients			
Omori, R.,Abu-Raddad, L. J.	2017	Sexual network drivers of HIV and herpes simplex virus type 2 transmission	East Africa (Kenya, India) and many SSA countries	effect of key sexual network characteristics on HSV transmission. Is HSV prevalence a good proxy for HIV prevalence.	HSV-2 prevalence could be used as HIV prevalence proxy. Many sexual network predictors of HSV
Abbai, Nathlee S.,Nyirenda, Makandwe,Naidoo , Sarita,Ramjee, Gita	2018	Prevalent Herpes Simplex Virus-2 Increases the Risk of Incident Bacterial Vaginosis in Women from South Africa	Southern Africa	effect of prevalent HSV on BV incidence	HSV positive women at higher risk of developing BV (AOR: 1,17)
Stoner MCD, Neilands TB, Kahn K, Hughes JP, Gómez-Olivé FX, Twine R	2019	Multilevel measures of education and pathways to incident Herpes simplex virus type 2 in adolescent girls and young women in South Africa	Southern Africa		

Looker, K. J.,Johnston, C.,Welton, N. J.,James, C.,Vickerman, P.,Turner, K. M. E.,Boi, M. C.,Gottlieb, S. L.	2020	The global and regional burden of genital ulcer disease due to herpes simplex virus: a natural history modelling study	global including Africa, Asia, Americas	proportion of GUD attributable to HSV	estimated 178 million people aged 15-49 experienced 1 or more GUD caused by HSV-2 in the world in 2016 (almost 5% of world pop). Highest in Africa and double in women than men.
